# 30-year record of Himalaya mass-wasting reveals landscape perturbations by extreme events

**DOI:** 10.1038/s41467-021-26964-8

**Published:** 2021-11-18

**Authors:** Joshua N. Jones, Sarah J. Boulton, Martin Stokes, Georgina L. Bennett, Michael R. Z. Whitworth

**Affiliations:** 1 School of Environmental Sciences, University of East Anglia, Norwich Research Park, Norwich, NR4 7TU UK; 2 School of Geography, Earth and Environmental Sciences, University of Plymouth, Drakes Circus, Plymouth, PL4 8AA UK; 3 College of Life and Environmental Sciences, Geography, University of Exeter, Amory Building, Rennes Drive, EX4 4RJ Exeter, UK; 4 AECOM, East Wing Plumer House, Tailyour Road, Plymouth, PL6 5DH UK

**Keywords:** Natural hazards, Solid Earth sciences

## Abstract

In mountainous environments, quantifying the drivers of mass-wasting is fundamental for understanding landscape evolution and improving hazard management. Here, we quantify the magnitudes of mass-wasting caused by the Asia Summer Monsoon, extreme rainfall, and earthquakes in the Nepal Himalaya. Using a newly compiled 30-year mass-wasting inventory, we establish empirical relationships between monsoon-triggered mass-wasting and monsoon precipitation, before quantifying how other mass-wasting drivers perturb this relationship. We find that perturbations up to 5 times greater than that expected from the monsoon alone are caused by rainfall events with 5-to-30-year return periods and short-term (< 2 year) earthquake-induced landscape preconditioning. In 2015, the landscape preconditioning is strongly controlled by the topographic signature of the Gorkha earthquake, whereby high Peak Ground Accelerations coincident with high excess topography (rock volume above a landscape threshold angle) amplifies landscape damage. Furthermore, earlier earthquakes in 1934, 1988 and 2011 are not found to influence 2015 mass-wasting.

## Abstract (web summary)

Significant mass-wasting perturbations can be caused by 5–30 year return period extreme rainfall and by earthquake induced landscape preconditioning damage.

## Introduction

In mountainous terrain, mass-wasting processes dominate landscape evolution^1–3^ posing risk to life and socioeconomic development^4,5^. Background rates of mass-wasting are driven by tectonic uplift^1,6^ and climate^7–9^, though their relative contributions over geological timescales are difficult to unravel^10^. At shorter timescales, background mass-wasting rates are perturbed by low-frequency, high-magnitude drivers, including extreme rainfall, earthquakes and floods^11–14^. Quantifying the mass-wasting caused by diverse drivers is thus fundamental in efforts to forecast and mitigate mass-wasting hazards in response to environmental change.

Compilation and comparison of erosion rates measured over different timescales can isolate the roles of different mass-wasting drivers. Such approaches typically utilise proxies, including cosmogenic nuclides or suspended sediment flux, to establish long-term background erosion rates against which shorter term perturbations captured by field sampling or remote sensing can be measured^2,12,13,15^. However, these approaches are inherently uncertain, with different methods over different timescales producing significantly different results^16^. Instead, a growing archive of remote-sensing data enables the compilation of sufficiently long datasets of mass-wasting from which background rates can be established, and perturbations above the background identified^7,17^. However, due to the time-consuming nature of developing such long-term datasets, rarely have studies successfully unravelled the relative impacts of interacting mass-wasting drivers.

A region with a complex set of mass-wasting drivers, and one of the highest rates of mass-wasting on Earth^4^ is the Himalayas. High rates of tectonic uplift and the Asia Summer Monsoon (ASM) drive high background rates of mass-wasting^15,18–20^, which are perturbed by events including floods^21^, extreme rainfall^22,23^ and earthquakes^3,24,25^. However, the relative impacts of these drivers remains unquantified, as most studies focus on the impacts of individual drivers only. Furthermore, whilst relationships between precipitation intensity and short-term suspended fluvial sediment flux in the Himalaya are well described^15,19,26^, an empirical relationship between ASM strength and ASM-triggered mass-wasting in central-eastern Nepal remains elusive^3^. This is problematic, as demonstrated by the 2015 *M*_w_ 7.8 Gorkha earthquake. As well as triggering over 24,000 coseismic landslides^24,27^, the Gorkha earthquake caused elevated rates of new monsoon-triggered mass-wasting in the 2015 monsoon season This is likely linked to surface damage caused by seismically induced ground motion^3^, an effect termed earthquake preconditioning^17,28^. However, the timescale and magnitude of this preconditioning perturbation remains uncertain, as without empirical relationships between ASM precipitation and mass-wasting, it is challenging to distinguish whether mass-wasting from 2016 onwards was perturbed above the rate expected given the ASM strength^3^. Accordingly, until empirical relationships between ASM strength and mass-wasting volume are defined, our ability to quantify mass-wasting perturbations due to extreme events across central-eastern Nepal is limited, thus impeding efforts to account for extreme events in mass-wasting forecasts and time-dependent landslide susceptibility models.

Here, we quantify the mass-wasting impacts of the ASM, extreme rainfall and earthquake preconditioning in the Nepal Himalaya. We use a new 30-year mass-wasting inventory for central-eastern Nepal to establish empirical relationship between metrics of ASM strength and mass-wasting, which are then used to calculate ASM strength-normalised mass-wasting rates between 1988 and 2018. These rates allow us to isolate and quantify the magnitudes and timescales of mass-wasting perturbations above that attributable to the ASM. As well as providing insight into the processes controlling landscape evolution, this permits further investigation into the characteristics and processes of earthquake preconditioning in the Himalaya.

## Results and discussion

**Fig. 1:**
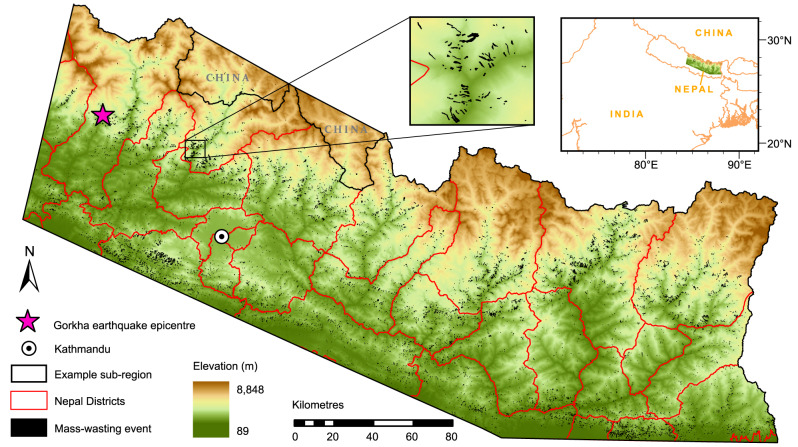
Location of the study region and all 12,290 mapped monsoon-triggered mass-wasting features. This includes a detailed view of a smaller sub-region demonstrating the detail of the mapped polygons. Also shown are the outlines of all Nepal Districts within the study region, including Kathmandu city and the Gorkha earthquake epicentre. Elevation data are derived from the ALOS World 3D (AW3D30) DEM developed by and copyrighted to the Japanese Aerospace Exploration Agency (JAXA).

Using visual inspection of Landsat 4/5/8 imagery, we mapped a 30-year inventory of rainfall-triggered mass-wasting over ~42,000 km^2^ of central-eastern Nepal between 1988 and 2018 (Fig. 1; 'Methods'). We mapped 12,920 moderate to large (>1000 m^2^) mass-wasting events; 10,138 of which were new failures and 2782 reactivations or remobilisations of previous failures. Mapping occurred across 29 time slices, each encompassing a single monsoon season (May–September) plus a varying number of months either side. The inventory does not include new coseismic or anthropogenic mass-wasting, but does include rainfall-induced reactivations/remobilisation of coseismic mass-wasting.

### Empirical relationship between the ASM and mass-wasting

To quantify empirical relationships between the ASM and mass-wasting, two measures of total mass-wasting for each time slice are derived: (1) the volume of all mapped features, including new landslides, reactivations, and remobilisations (‘New + RR’); and (2) the volume of new features only, with reactivations and remobilisations removed (‘New Only’). These measures allow isolation of new post-earthquake mass-wasting related to earthquake damaged bedrock (i.e. earthquake preconditioning) from reactivations and remobilisations of coseismic and pre-existing mass-wasting. For each measure, mass-wasting volumes were calculated using global area–volume scaling relationships^29^, both for estimated scar areas and total areas (combined scar, depositional, and runout zones) ('Methods').

**Fig. 2:**
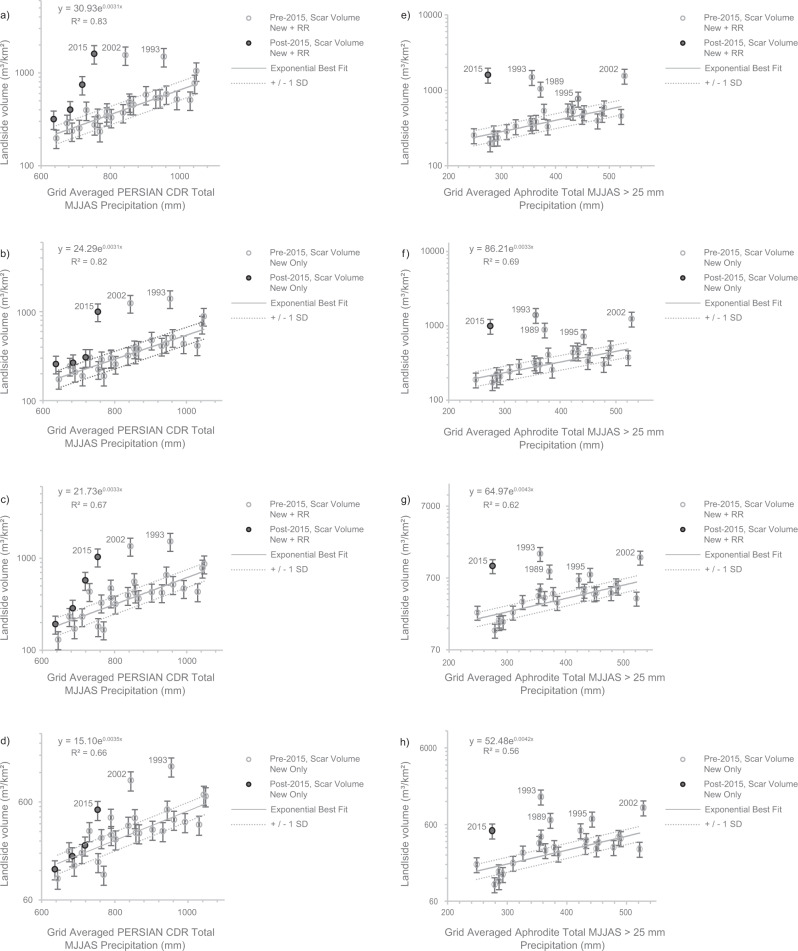
Empirical relationships between measures of mass-wasting volume and monsoon strength across central-eastern Nepal. **a**–**d** Empirical relationships between measures of mass-wasting volume (m^3^/km^2^) and PERSIANN-CDR total MJJAS precipitation for **a** total ‘New + RR’ volume, **b** total ‘New Only’ volume, **c** scar ‘New + RR’ volume and **d** scar ‘New Only’ volume. **e**–**h** Empirical relationships between measures of mass-wasting volume (m^3^/km^2^) and APHRODITE total MJJAS precipitation >25 mm for **e** total ‘New + RR’ volume, **f** total ‘New Only’ volume, **g** scar ‘New + RR’ volume and **h** scar ‘New Only’ volume. Where, in all cases ‘New + RR’ refers to the combined volumes of both new failures and reactivations/remobilisations and ‘New Only’ refers to just the volumes of new failures, with reactivations and remobilisations excluded. The exponential best fits shown on these graphs apply to the non-anomalous pre-2015 points only, with all anomalous points labelled individually. The post-2015 points are also shown for reference, as are the ±1 standard errors on the best-fit equations.

We then correlate all measures of mass-wasting volume for pre-Gorkha earthquake years with proxies for ASM strength derived from two rainfall products: PERSIANN-CDR^30,31^ and APHRODITE^32^ ('Methods'). For both PERSIANN-CDR and APHRODITE, we use several reported proxies for ASM strength^3,33^: total May to end-September (MJJAS) precipitation, total precipitation from 15th July–end-September, total MJJAS precipitation >25 mm (sum of all precipitation days with total rainfall values >25 mm), and total precipitation >25 mm from 15th July–end-September (sum of all precipitation days within this time period with total rainfall values >25 mm). We avoid measures of monsoon strength such as the SASMI^34^ as these are derived over regional scales and do not capture local changes in monsoon conditions. As previously observed in western Nepal^33^, for the PERSIANN-CDR data, total MJJAS precipitation provides the best fit to the mass-wasting data (Fig. 2a–d), while for APHRODITE, it is total MJJAS precipitation >25 mm (Fig. 2e–h; Supplementary Figs. S1–3). Thus, from here onwards, the term ‘ASM strength’ refers to total MJJAS precipitation (mm/grid) for PERSIANN-CDR, and total MJJAS > 25 mm (mm/grid) for APHRODITE. Of the 24 pre-Gorkha earthquake years included in these correlations, we find that mass-wasting volume per unit area increases with total grid-averaged precipitation, with potential anomalies in 1989, 1993, 1995, and 2002 (*R*^2^ = 0.69–0.83 for non-anomalous years using PERSIANN-CDR (Fig. 2a–d) and *R*^2^ = 0.56–0.67 for non-anomalous years using APHRODITE (Fig. 2e–h)).

**Fig. 3:**
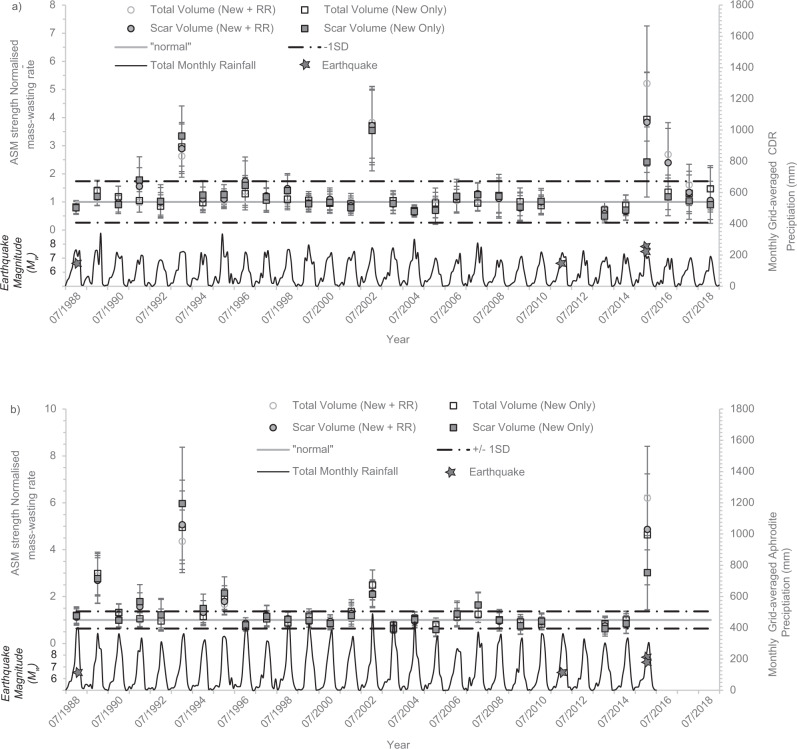
ASM strength-normalised rates of mass-wasting between 1988 and 2018. These rates are shown for **a** the normalisation using the PERSIANN-CDR data and total MJJAS precipitation and **b** the normalisation using the APHRODITE data and total MJJAS precipitation >25 mm. In both cases, most years fall within a narrow window around the normal, with perturbations in 1993, 2002 and 2015 in (**a**), and 1989, 1993, 1995, 2002 and 2015 in (**b**). The occurrences of historical *M*_w_ > 6.0 earthquakes are shown. Also depicted are the monthly grid-averaged PERSIANN-CDR (**a**) and APHRODITE (**b**) precipitation totals across the study region between 1988 and 2018. The errors in the normalised rate include the standard error in the data points used to calculate the prediction equations in Fig. 2, an assumed standard deviation of 20% on the mapped mass-wasting feature areas, and the standard deviations reported in the Larsen et al.^29^ area–volume conversion parameters. Assuming that these errors are uncorrelated, they were combined using standard Gaussian propagation to obtain the final error bar uncertainties.

Following previous methodologies^17^ (see 'Methods'), we then use the best-fit empirical relationships (Fig. 2a–h) to calculate the predicted volumes of mass-wasting expected in each time slice based on that year’s total grid averaged ASM strength. Then, for all measures of volume, by taking the ratio of the actual mapped volumes to the predicted volumes, we derived ASM strength-normalised rates of mass-wasting for each of the 29 time slices between 1988 and 2018. These rates show that, for both rainfall products, most time slices fall within a narrow band of mass-wasting around the expected normalised value of one, with several years perturbed above this. For the PERSIANN-CDR normalisation, there are perturbations above +1 SD of the normal in 1993, 2002 and post-2015 (Fig. 3a). For the post-2015 perturbation, if coseismic reactivations and remobilisations are considered, then 2015 and 2016 are perturbed above the expected monsoon scaling. However, when considering only new failures, only 2015 is perturbed, as reported previously^3^. For the APHRODITE normalisation, the years 1989, 1993, 2002 and 2015 are perturbed above +1 SD of the normal, with another possible perturbation in 1995 (Fig. 3b).

**Fig. 4:**
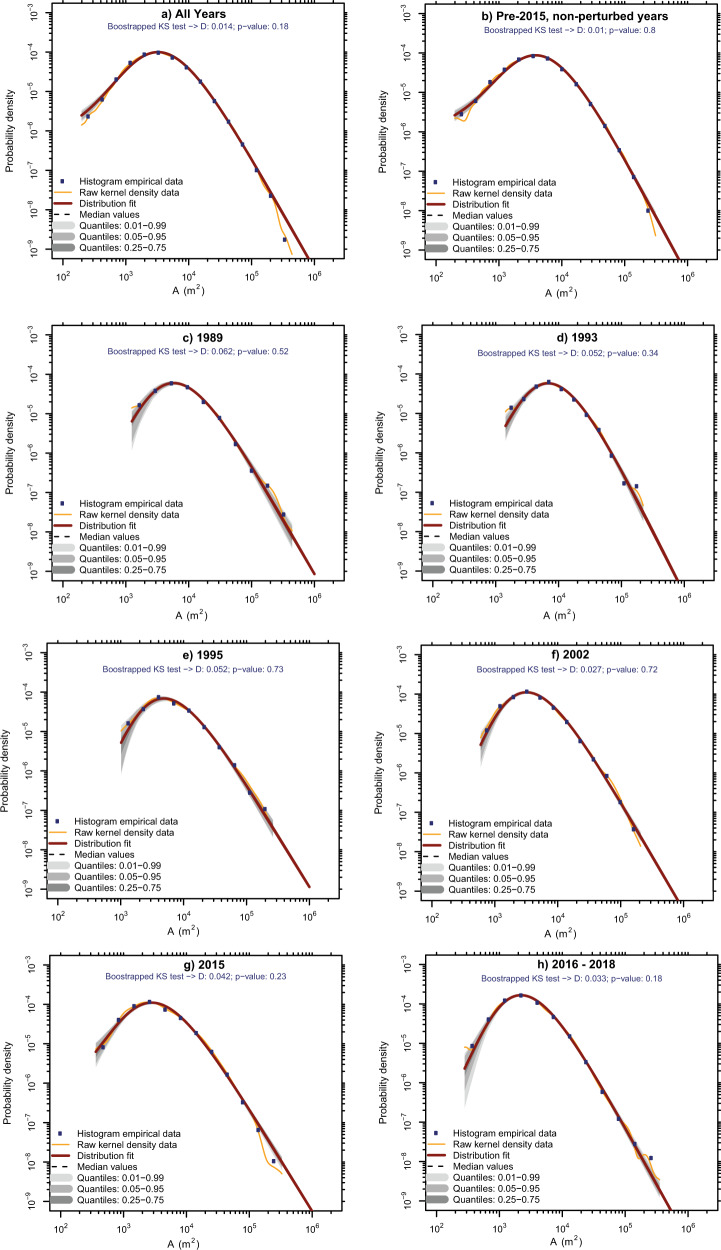
Probability density functions (PDFs) of mass-wasting area fitted with inverse-gamma power law distributions. These are shown for **a** all years, **b** the pre-2015 non-perturbed years, **c** 1989, **d** 1993, **e** 1995, **f** 2002, **g** 2015, **h** post-2015. All PDFs and fitted distributions were calculated and plotted using the Landsat Software (version 10). Note that a *p*-value > 0.01 indicates that the three-parameter inverse-gamma distribution provides a good fit to the actual data, whilst the D-value gives the maximum distance between the actual data and the fitted distribution.

As the ASM strength-normalised mass-wasting rate accounts for variance in ASM precipitation, the perturbations should be attributable to infrequent high-magnitude mass-wasting drivers not accounted for by the metrics of ASM strength. However, before assuming this, it should be confirmed that these perturbations are not actually caused by a small number of anomalously large landslide events. We achieve this using two approaches ('Methods'). (1) Before correlating mass-wasting with ASM strength, we removed the largest landslides of each year if its scar area was greater than twice that of the second largest. This ensures that large landslides influenced by progressive failure across several monsoon seasons are not incorrectly attributed to a single time slice^3^. (2) We fitted three-parameter inverse-gamma distributions to the probability density functions (PDFs) of mass-wasting area for all years combined, all pre-2015 non-perturbed years, 1989, 1993, 1995, 2002, 2015 and all post-2015 years (Fig. 4a–h). If the inverse-gamma distributions fitted to each subset have similar scaling exponents (where a larger exponent indicates that larger mass-wasting events are contributing proportionally less to the overall area of that subset) and rollovers (the size above which power law behaviour applies), then we can rule out that the observed perturbations are caused solely by statistical anomalies in mass-wasting size.

Scaling exponents fall between 1.8 and 2.2 for all subsets except 1995 and 2015, which had lower exponents of 1.6. Similarly, the rollovers of most subsets fall between 2000 and 6000 m^2^, apart from 1989 and 1993, which had rollovers of 6000 and 7000 m^2^. Since the scaling exponents are similar above comparable cut-offs, the area-frequency distributions can be described as scaled versions of one another, though with 2015 and 1995 having slightly higher proportions of relatively larger landslides. This suggests that the observed perturbations are not solely attributable to changes in mass-wasting size, but are due to physical processes increasing the frequency of all sizes of mass-wasting.

### Impacts of extreme rainfall

The ASM strength-normalised rates identify mass-wasting perturbations in 1993, 1995 and 2002 that are not coincident with seismic activity >*M*_w_ 6.0 (Fig. 3a, b). If these perturbations are associated with rainfall, we propose two explanations for their occurrence. One, they are due to years of overall intense monsoon activity that are just poorly predicted by the normalisation method. Or two, they are due to significant rainfall events that occurred within the monsoon seasons, but were too localised to be captured by the monthly precipitation estimates. The time series of monthly precipitation totals (Fig. 3a, b) show that the total monsoon rainfall for these years was not anomalously high. However, the perturbations in 1993 and 2002 were both coincident with ‘cloud-outburst’ extreme rainfall events. On 19–20 July 1993, >540 mm of rainfall in 24 h fell across a 500 km^2^ region of the Kulekhani watershed, 30 km southwest of Kathmandu, causing over 1500 fatalities and triggering over 300 landslides^22^. Similarly, on 23 July 2002, >300 mm of rainfall in 24 h fell across a 14,000 km^2^ region of south-central Nepal, causing over 427 fatalities and triggering 73 debris slides^35,36^.

This suggest that the 1993 and 2002 mass-wasting perturbations were the result of short-lived, localised, extreme rainfall events that were not captured in the measures of ASM strength. This raises several questions. How extreme were the 1993 and 2002 events? Did similarly extreme rainfall events affect the other perturbations? Have similarly extreme rainfall events occurred without triggering significant mass-wasting? And what are their periods?

To answer these questions, it is necessary to define how extreme the 1993 and 2002 cloud-outburst storm events were. To do this, we exploit the daily APHRODITE precipitation record (1951–2015) to calculate *Z*-score anomalies for every monsoon season (MJJAS) day across each of the 84 APHRODITE grids that encompass our study region. For each separate rainfall grid cell, the mean and standard deviations of all monsoon season days were calculated, and individual daily *Z*-scores obtained. A *Z*-score anomaly defines how many standard deviations separate a given observation from the mean of the population containing this observation. *Z*-score anomalies were used as they are a commonly used effective method for semi-quantitatively assessing changes in environmental data^37,38^.

**Fig. 5:**
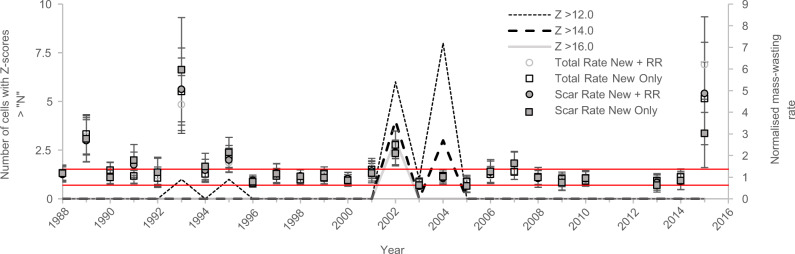
Number of daily cells per monsoon season that had *Z*-score anomalies greater than 12, 14 and 16. For reference, the normalised rates and associated ±1 SD (red lines) from Fig. 3b are also shown.

For the 1993 and 2002 events, peak *Z*-scores were ~12 (Supplementary Fig. S4) and 16–19, respectively. To identify whether any similarly extreme rainfall events had occurred across the mapped time period, we then extracted all days with *Z*-scores exceeding thresholds of 12, 14 and 16, and correlated these with the normalisation results from Fig. 3b (Fig. 5a). Only two other years observed rainfall events with *Z*-scores >12: 1995, which also experienced a minor mass-wasting perturbation, and 2004, which did not. This suggests that a rainfall *Z*-score threshold of ≥12, relative to the 1951–2015 mean, is required to induce a significant mass-wasting perturbation. The perturbations in 2015 and 1989 do not coincide with any anomalously high rainfall, with neither year observing days with *Z*-scores >10, suggesting that extreme rainfall alone cannot explain these perturbations.

Interestingly, the 2004 monsoon season did not observe a mass-wasting perturbation, despite experiencing eight cells (across the entire region) with *Z*-scores >12 and three cells with *Z*-scores >14. With the exception of 2002, this year observed the most extreme rainfall between 1988 and 2015. Given that all eight of the cells experiencing extreme rainfall in 2004 occurred after 15 June, the lack of a landslide response is unlikely to be attributable to incompletely saturated hillslopes^3,39^. However, there are three other potential explanations for why the 2004 rainfall did not induce a mass-wasting response.

**Fig. 6:**
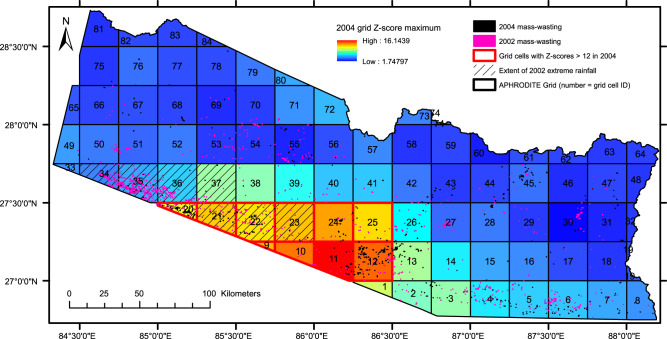
The locations and IDs of the 84 APHRODITE rainfall grid cells across the study region. Maximum *Z*-scores from the 2004 monsoon seasons are depicted alongside the 2004 mass-wasting. Also shown is the extent of the 2002 extreme rainfall, and the mass-wasting from 2002 across the study region.

The first explanation relates to the temporal distribution of the 2004 extreme rainfall. Of the eight cells in 2004 that exhibited *Z*-scores >12, none were in the same cell on consecutive days. This is potentially important, as consecutive high-intensity rainfall days will be more efficient at triggering landslides. However, a lack of consecutive high *Z*-score days in 2004 is considered an unlikely explanation, as of all the other perturbations, only one cell in 2002 (cell 21; Fig. 6) experienced two consecutive days with *Z*-scores >12.

The second potential explanation is that rainfall could have induced less landsliding in 2004 because the landscape had yet to recover from the landslide perturbation in 2002. This concept of landscape recovery^40^ is the idea that a major exhumation event will exhaust a landscape of soil material available to fail, thus transiently limiting future landsliding until the landscape has re-accumulated unstable regolith material. The 2004 rainfall event was partially coincident with the 2002 event (Fig. 6) suggesting that landscape recovery (or lack of) may explain the subdued landslide response in 2004. If this is correct, then the cells coincident with both the 2002 and 2004 events (cells 20, 21, 22 and 23; Fig. 6) would have less mass-wasting in 2004 relative to their *Z*-scores than the cells that were only impacted in 2004 (cells 9, 10, 11, 12, 24). To test this hypothesis, the percentage change in 2004 monsoon-triggered mass-wasting relative to the non-perturbed mean for each cell was calculated ('Methods'). The 2004 event cells coincident with 2002 have percentage mass-wasting changes of 20–435% in response to *Z*-scores of 11–13. In comparison, the cells not coincident with the 2002 event had percentage changes in mass-wasting of 220–700% in response to *Z*-scores of 12–16. As such, while the non-coincident cells did observe more mass-wasting in 2004 than the coincident cells, they also observed higher *Z*-scores. It thus remains inconclusive whether a lack of landscape recovery can explain why 2004 did not observe a mass-wasting perturbation. Furthermore, the concept of landscape recovery is contradicted by the observations shown here and in other studies for post-seismic landsliding^3,14,17,41^, where landsliding actually increases immediately following a major exhumation event.

Third, the lack of landslide response could result from inaccuracy and/or misallocation of the rainfall data. The APRHDOITE grid cells are coarse (~30 km resolution), and the cells for the 2002 and 2004 events cross the boundary of the study region. Consequently, the high 2004 *Z*-scores could be caused by rainfall located just outside of the study region, and thus the observed landsliding may have occurred in response to less extreme local rainfall than is suggested by the larger-scale *Z*-score anomaly. In the absence of higher resolution time series data, it is challenging to quantify whether such inaccuracy exists, but it is an issue that should be considered when interpreting the results.

Overall, while it remains unclear exactly why 2004 did not observe a mass-wasting response, this analysis does show that the 1993, 1995 and 2002 perturbations all coincide with years that experienced rainfall *Z*-scores >12, indicating that this is a threshold for which significant mass-wasting can be induced. As such, it would be useful to know the return periods of such events. From the ASM-normalisations (Fig. 3a, b), two extreme rainfall-induced mass-wasting anomalies occurred over a 30-year period, suggesting that across the entire study region, such perturbations have ~15 year return periods. Furthermore, based on the full 64-year time series of APHRODITE rainfall, the return periods across the entire study region of rainfall events capable of causing these perturbations (*Z*-scores >12 and >16) are 5–30 years (15 and 2 events recorded over 64 years).

Finally, it is worth highlighting that all results presented here pertaining to extreme rainfall should be interpreted cautiously, as while high *Z*-scores generally coincide with a mass-wasting perturbation, the specific relationship between *Z*-score magnitude and mass-wasting perturbation magnitude is inconsistent. For example, the 1993 mass-wasting perturbation is the largest, but has smaller *Z*-scores than 2002 and 2004. This could be due to inaccurate or misallocated rainfall data, as ~500 mm of rainfall reportedly fell in 24 h during the 1993 event^22^, but the APHRODITE data show a combined daily total of only 274 mm across the cells that observed a mass-wasting response. An additional cause of uncertainty is the potential sub-optimal quantification of the extreme. As mentioned above, the daily *Z*-score approach is unsophisticated and does not consider anomalous rainfall over shorter or longer timescales. As such, future work could attempt to refine the relationships between extreme rainfall and mass-wasting by considering *Z*-score anomalies across multiple days or hours using higher temporal resolution rainfall products.

### Impacts of earthquakes

There are two main processes by which large (>*M*_w_ 6.0) earthquakes impact mass-wasting. First, they trigger coseismic landslides that can be remobilised by subsequent rainfall or other exhumation events^41–43^. Second, earthquake ground motion can cause landscape damage that induces enhanced rates of new post-earthquake mass-wasting^3,17^; a process termed earthquake preconditioning^28^. Earthquake preconditioning has been observed following earthquakes in different geomorphic settings. For example, the 1999 *M*_w_ 7.7 ChiChi earthquake, Taiwan, caused a 2–5 year factor of 10 increase in subsequent new typhoon-triggered landsliding^17^. Similarly, the 2015 *M*_w_ 7.8 Gorkha earthquake caused a factor of 4–8 increase in new monsoon-triggered mass-wasting during the 2015 monsoon season^3^. However, the full timescale of 2015 preconditioning remains unconstrained as, until now, it has not been possible to isolate the earthquake preconditioning impacts from the monsoon in 2016–2018.

Here, our normalisation using the PERSIANN-CDR data (Fig. 3a) allows for the impacts of the 2015 earthquake and post-2015 monsoon to be separated, providing new insight into the magnitude and timescales of the 2015 preconditioning. Our normalisation with both PERSIANN-CDR and APHRODITE corroborates previous results^3^, showing that all measures of mass-wasting were perturbed in 2015, with ‘New + RR’ mass-wasting (which comprises all landslides, including rainfall-induced remobilisations of coseismic landslides) perturbed by a factor of 3.8–6.2 and ‘New Only’ mass-wasting (reactivations and remobilisations excluded) perturbed by a factor of 2.4–4.6 (Fig. 3a, b). In 2016, ‘New + RR’ mass-wasting was still perturbed by a factor of 2.4–2.7, but the ‘New Only’ rate was within +1 SD of the normal (Fig. 3a). In 2017 and 2018, both ‘New + RR’ and ‘New Only’ rates were back within ±1 SD of the normal (Fig. 3a).

These results provide insights into remobilisation timescales of coseismic material, and of earthquake preconditioning associated with the Gorkha earthquake. For earthquake preconditioning, enhanced rates of new mass-wasting are only observed in 2015, with new mass-wasting in 2016 within +1 SD of that expected given the monsoon strength. This suggests that Gorkha earthquake preconditioning lasted for 5–14 months, i.e., until the start of the 2016 monsoon season. This timescale is slightly shorter than the 2–5 year preconditioning period observed in Taiwan following the ChiChi earthquake^17^, but similar to other observations in Nepal^3,42^ showing that rainfall induced debris flows and landslides following the Gorkha earthquake were anomalous in 2015 only. For the remobilisation of coseismic material, enhanced rates of mass-wasting when including remobilisations and reactivations continues into 2016, but not 2017, suggesting a recovery time of 17–24 months. This recovery time is shorter than the 6–8 year period over which anomalous fluvial sediment export was observed following the ChiChi earthquake^41^. This timescale difference is likely because our approach only identifies large-scale remobilisations and reactivations, whereas measures of fluvial sediment export are more sensitive to small-scale changes that would not be visible at the mapping resolution used here. The APHRODITE-based normalisation also identifies a perturbation in 1989. This was the first full monsoon season following the 21/08/1988 *M*_w_ 6.9 earthquake. In this case, both the earthquake preconditioning perturbation (‘New Only’ rate) and increase in reactivations and remobilisations (‘New + RR’ rate) are observed in 1989 only, suggesting a recovery period for these processes of no more than 13–20 months, similar to that observed for the Gorkha and ChiChi earthquakes.

**Fig. 7:**
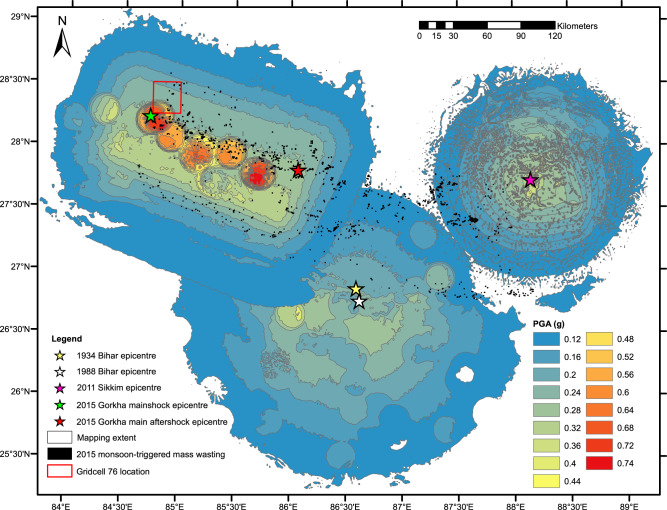
The locations of the epicentres of the 2015 *M*_w_ 78 Gorkha earthquake main shock, the 2015 *M*_w_ 7.3 largest aftershock, the 2011 *M*_w_ 6.9 Sikkim earthquake, the 1988 *M*_w_ 6.9 Bihar earthquake and the 1934 *M*_w_ 8.0 Nepal–Bihar earthquake. Also shown are the USGS estimated PGA distributions for the 2015 Gorkha main shock, and the 1988 and 2011 events^45,84,85^ and the location of possible outlier grid cell 76.

**Fig. 8:**
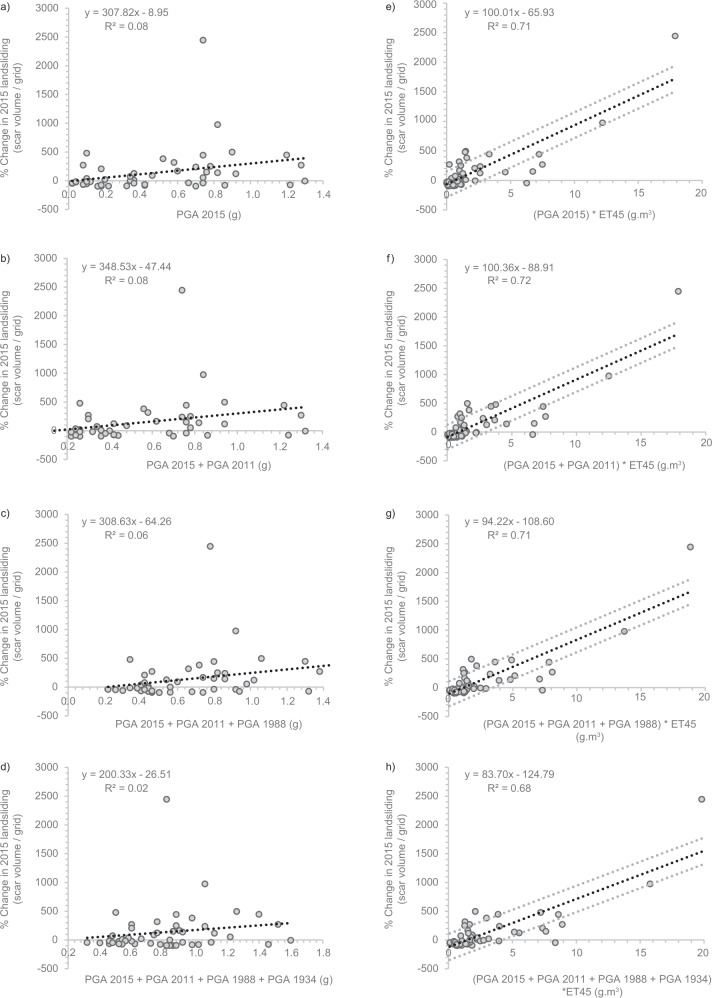
Correlations between maximum summed PGA and percentage change in monsoon-triggered 2015 mass-wasting. This is shown for **a** PGA in the 2015 main shock and largest aftershock. **b** the summed PGA from **a** plus the PGA from 2011. **c** The summed PGA from **b** plus the PGA from 1988. **d** The summed PGA from **c** plus the PGA from 1934. **e**–**h** show the same correlations as **a**–**d**, but with summed PGAs multiplied by excess topography above a threshold angle of 45°. The linear best fits are shown with ±1 standard error in each case.

While this study and others^17^ quantitatively constrain the magnitudes and timescales of short-term earthquake preconditioning, the spatial distributions, processes, and causal mechanisms remain under-investigated. It has been proposed that short-term preconditioning occurs via near-surface earthquake damage that is rapidly exploited by subsequent rainfall as new failures^17^. However, while subsurface seismic velocities have been shown to track coseismic damage and landscape recovery^44^, what controls the spatial distributions of seismically induced landscape damage remains uncertain. Therefore, to investigate the controls on earthquake preconditioning damage, we combine our 2015 monsoon-triggered landslide inventory with Gorkha earthquake USGS ground motion data^45^ to examine how the 2015 excess mass-wasting relates to the Gorkha earthquake PGA and topographic factors. To do this we need to move from regional-scale analysis to localised, grid-scale analyses. Accordingly, we divided our study region into the same 84 grid cells as used for the *Z*-score analysis (Fig. 6). Then, we calculated ('Methods') for each grid cell the percentage change in 2015 monsoon-triggered mass-wasting relative to that grids non-perturbed mean, and the summed maximum PGA from both the *M*_w_ 7.8 Gorkha earthquake main shock and *M*_w_ 6.3 aftershock for each grid cell with <10% snow cover (Fig. 7). Surprisingly, this analysis shows no correlation between 2015 mass-wasting and PGA (Fig. 8a). Therefore, does PGA alone induce earthquake preconditioning? As seismic ground motion undergoes amplification when travelling across topographic excesses^46–48^, earthquake preconditioning may preferentially occur where high PGA is coincident with high excess topography, where excess topography is defined as the volume of rock mass above a landscape’s threshold angle^49^.

To investigate this, we calculate the average excess topography of each grid cell for five landscape threshold angles (25°, 30°, 35°, 40°, 45°; Methods). For each threshold of excess topography, we calculate for every grid cell the product of maximum summed Gorkha earthquake PGAs and average excess topography, and plot weighted ‘PGA-Excess Topography’ values against each grid cells 2015 percentage mass-wasting change. This results in a significant improvement of fit compared to using PGA alone, with *R*^2^ increasing from 0.08 to 0.71 for a 45^°^ threshold (Fig. 8b). This is consistent across all excess topography thresholds (Supplementary Figs. S5 and S6), but with a slight increase in *R*^2^ as the threshold increased from 25° to 45°. This was also consistent when only summing PGAs >0.1 and 0.2 g, though with lower *R*^2^ values (Supplementary Figs. S7 and S8). These PGA values have been identified as possible thresholds which must be exceeded for landslides to be induced^50,51^; however, these new results suggest that lower PGA values can still contribute to preconditioning, even if they do not directly trigger coseismic landslides.

Additionally, topographic slope angle has been shown to be critical in controlling landscape susceptibility to coseismic landsliding^52,53^. Therefore, we repeated the above analysis but with average slope values per grid cell instead of average excess topography. However, the combination of slope and PGA does not exhibit a strong relationship with excess mass-wasting (Supplementary Fig. S9), suggesting that excess topography is a better metric for predicting post-seismic landscape preconditioning. However, it should be caveated here that the *R*^2^ increase gained from using PGA to PGA combined with excess topography is strongly controlled by one outlier in grid-cell 76 (see Figs. 6 and 7). This grid cell had the highest PGA-excess topography values as this was one of the few cells of its latitude (where high excess topography values are widespread) to overlap with the greatest Gorkha earthquake PGAs. This cell also had the highest 2015 mass-wasting increase, as it experienced 3.6 × 10^6^ m^3^ of landsliding (including one landslide >220,000 m^2^), whereas in the preceding 25 years this cell experienced >1.0 × 10^6^ m^3^ of landsliding in only three other years.

Overall, our analyses suggest that short-term earthquake preconditioning damage is concentrated where PGA and high excess topography coincide. This noteworthy result could allow for more accurate predictions of where and how much preconditioning should be expected in a landscape following a given magnitude earthquake. However, a similar relationship was not observed for the 1988 earthquake (Supplementary Fig. S10). Reasons for this could be: (1) the 1988 earthquake had lower PGAs than 2015 (maximum 0.28 g in 1988 compared to >0.74 g in 2015); (2) the region impacted by the 1988 event was to the south of our study region, where excess topography values are low, or (3) the 1989 perturbation was actually caused by rainfall. Despite not having *Z*-scores as high as observed in 1993 or 2002, 1989 did observe higher *Z*-scores than 2015 (scores of 10, compared to 8). However, rainfall has already been discounted as a causative mechanism due to the relatively low *Z*-scores in 1989 compared to 2002 and 1993. Accordingly, we suggest that no single explanation can explain the 1989 perturbation and that it could be caused by a combination of the earthquake and rainfall, or an artefact arising from imperfect rainfall data and normalisation (e.g. Fig. 3a, b).

This analysis provides insight into short-term preconditioning^17^, but does not consider decadal-scale preconditioning. For example, coseismic landslides associated with the 1968 *M*_w_ 7.1 Inangahua (New Zealand) earthquake occurred at higher rates where the landscape was impacted by the earlier 1929 *M*_w_ 7.7 Buller earthquake, suggesting that lasting landscape damage due to the earlier event was compounded by the second event. Here, we investigate whether the 2015 monsoon-triggered perturbation was similarly affected by long-term damage from earlier earthquakes in 1934 (*M*_w_ 8.0), 1988 (*M*_w_ 6.9) and 2011 (*M*_w_ 6.9) (Fig. 7). To test this, we repeat our PGA-excess topography correlations, but this time cumulatively summing the maximum PGA observed per grid cell from the 2015, 2011, 1988 and 1934 earthquakes. These results, with PGA alone and PGA multiplied by excess topography at a threshold of 45°, are shown in Fig. 8c–h (for correlations of PGA with other excess topography thresholds see Supplementary Figs. S5 and S6). If earlier earthquakes had a damage legacy that significantly compounded the Gorkha earthquake damage, the inclusion of their PGA should improve the observed fit between the percentage change in 2015 ASM-triggered mass-wasting and 2015 PGA-excess topography. However, the inclusion of 2011 PGA causes no significant improvement (*R*^2^ increase from 0.71 to 0.72; Fig. 8d), while including the PGA from 1988 and 1934 worsens the fit (Fig. 8f–h). There is thus no evidence that past earthquakes contributed to elevated ASM-triggered mass-wasting in 2015. There are several potential explanations: (1) the time since the events in 1988 and 1934 is too long, and thus the landscape damage caused by them has already been exploited. The 1934 event was 81 years before Gorkha, over twice the time between the two earthquakes observed to induce preconditioning in New Zealand. (2) The magnitudes of the 2011, 1988 and 1934 events were too small to induce widescale damage. This explanation is possible, as while the 1934 event was of comparable magnitude to 2015, the 2011 and 1988 magnitudes were lower. (3) The 1988 and 1934 events were located too far from the region impacted by Gorkha for any significant damage to overlap. This is the most likely explanation, as despite being of magnitudes that should be capable of inducing landscape damage, both the 1934 and 1988 events occurred in southern Nepal, with no PGAs >0.2 g in 1988 overlapping with PGAs >0.1 g in 2015 (Fig. 7). The 2011 event also had no overlap with 2015 at PGAs > 0.1 g, potentially explaining why this event also had no impact on 2015 excess monsoon-triggered mass-wasting.

In conclusion, by quantifying a previously unknown empirical relationship between ASM strength and total mass-wasting we have isolated and investigated mass-wasting perturbations due to extreme rainfall and 2015 Gorkha earthquake landscape preconditioning. We find that: (1) extreme, 5–30-year return period rainfall events can induce mass-wasting perturbations. (2) The 2015 perturbation is controlled by short-term Gorkha earthquake-induced landscape preconditioning. (3) The signature of landscape preconditioning is controlled by the coincidence of PGA and excess topography. (4) Earlier large magnitude earthquakes in 1934, 1988 and 2011 did not compound the 2015 preconditioning, suggesting that longer-term preconditioning damage was not a major driver of landsliding here.

These results have implications for mass-wasting hazard and susceptibility modelling. First large uncertainties remain in predicting how climate change may affect landsliding over the Himalaya^23^. These results assist reducing this uncertainty since the empirical relationships between ASM strength and mass-wasting can provide quantitative assessments of expected changes in ASM-triggered mass-wasting across the Himalaya when combined with possible future ASM strength scenarios^54–58^. Furthermore, if future climate change scenarios suggest an increase in the occurrence of 5–30-year return period rainfall events^59,60^, then mass-wasting perturbations such as those in 1993 and 2002 will become more frequent and pose an increasingly pervasive hazard. Second, existing mass-wasting susceptibility models are typically time-independent, implicitly assuming that the conditions that produced past mass-wasting will remain the same in the future^61,62^. However, our results show that earthquake preconditioning can cause transient, time-dependent mass-wasting perturbations. This suggests that post-earthquake rainfall-triggered landslide susceptibility modelling should account for the transient topographic signature of earthquakes. The finding that preconditioning is controlled by the product of PGA and excess topography is especially useful, since it provides a framework for which preconditioning-induced mass-wasting can be modelled.

## Methods

### Mass-wasting mapping

Mass-wasting events were mapped using Landsat imagery. Landsat products were selected as they provide the longest continuously acquired space-based archive of the Earth’s surface and are the only product to contiguously cover Nepal over the 30-year time period we aimed to map. At the time of writing, Landsat imagery is freely available via the USGS Earth Explorer platform^63^. Mapping was conducted using Landsat 4/5 in years 1988–1999 and 2004–2010, Landsat 7 in years 2000–2003, and Landsat 8 in years 2013–2018. Landsat 7 could not be used for years 2004–2012 because it lost its scan-line corrector in 2003, with >35% imagery data loss^64^. This was insufficient for mapping, so we reverted to Landsat 4/5 imagery until Landsat 8 imagery became available in 2013. Consequently, 2011 and 2012 were not mapped as this period was only covered by Landsat 7 imagery. 2013 was mapped as normal using Landsat 8 pre-post monsoon imagery (i.e., landslides from 2011 and 2012 were excluded from the inventory, with only new landslides occurring post-2012 mapped). Landsat products have a 16-day temporal resolution. However, in Nepal, with cloud cover pervasive throughout the year, pre- and post-ASM images were acquired between start October and end April, i.e., either side of the May–September monsoon season. Note that the post-imagery used to map a given time slice was typically used as the pre-imagery for the next time slice, thus ensuring that mapping was continuous, with no significant time gaps. The name and date of the satellite imagery used to map each year, as well as a summary of each year’s mass-wasting data, are shown in the Supplementary Materials (Table S1). Landsat 4/5 has a spatial resolution of 30 × 30 m, while Landsat 7/8 was pansharpened with panchromatic imagery to 15 × 15 m. Thus, the minimum mappable feature size was ~1000 m^2^.

Mass-wasting features were identified by visual comparison between pre- and post-imagery for a given year. Images were viewed as false RGB images with red band = infrared, green band = green and blue band = blue. This combination was used because the reflectivity differences strongly highlighted vegetated areas relative to bare earth. If a new bare-earth feature appeared in the landscape between the pre- and post-imagery and had the typical shape and location of a mass-wasting event it was delineated as a polygon. All types of rainfall-triggered landslides^65^ were included in the inventory, i.e., landslides were not differentiated by type. Care was taken to avoid mapping features related to land clearance, such as deforestation and cut-and-fill practices, including features due to undercutting by roads or channels. All mapped mass-wasting events included the combined scar, runout and depositional zones, as these were not distinguishable at the spatial resolution of the imagery. Steps were taken to avoid mass-wasting amalgamation, i.e. separation of mass-wasting events whose runouts combined to form one single deposit, as this is known to impact mapping results^66^. Mass-wasting events that scarred or disturbed vegetation/material within the boundary of a previous landslide were recorded as reactivations (failures involving the displacement of previously undisturbed material that initiated from or intersected with the boundary of a previous failure), although image resolution may mean some of these could have been remobilisations (movements of previously disturbed material only) rather than reactivations. In total, 12,920 moderate to large (~1000 m^2^) mass-wasting events were mapped across 29 separate time slices from 1988 to 2018 (see Supplementary Data 1 for the geometric and satellite information of each mapped feature).

Each time slice included a given year’s monsoon season (May–September) plus a varying number of non-monsoon months either side. The variation in the number of October–April months included in each time slice was an unavoidable consequence of extensive cloud cover across the Himalayas. However, since our time slices had varying lengths, both between time slices and within time slices (as several tiles were required to map the entire study region, and invariably these tiles had different acquisition dates and cloud cover), it is necessary to consider the effect of this on our results. Our analysis of ASM- and extreme rainfall-triggered mass-wasting assumes that all mass-wasting was triggered during a given time slice’s monsoon season. As these time slices include months outside of the monsoon period, it is possible that some rainfall-triggered events did not occur during the monsoon. However, it is known that this region experiences little rainfall-triggered landsliding outside of the monsoon period^5,67^. Indeed, we find no correlation between the number of non-monsoon months within a time slice and number of mass-wasting events mapped (Supplementary Fig. S11). Furthermore, we find no correlation between the total rainfall in the non-monsoon months between time slices and the deviation of a time slice from the normalisation in Fig. 2b (Supplementary Fig. S12). This suggests, as expected, that variable time slice length cannot explain the normalisation results. To further reduce error in mapping procedure, we applied a 20% assumed error to all mapped mass-wasting areas. This incorporation should account for variability in mapped mass-wasting caused by including non-monsoon months, as well as for any erroneously included mass-wasting events attributable to non-rainfall dominated processes such as undercutting by river channels or earthquakes. Note that road-associated and coseismic mass-wasting events were explicitly not included in this inventory, though rainfall induced reactivations and remobilisations of coseismic mass-wasting are included. Coseismic mass-wasting events in 2015 were identified and avoided using the dataset of Roback et al.^24^. Furthermore, possible coseismic events triggered by an *M*_w_ 6.9 event that occurred midway through the 1988 monsoon season and affected a small portion of the study region were identified and avoided based on their slope position^52,68^.

### Scar area and volume derivations

As stated, the satellite imagery resolution allowed mass-wasting features to be mapped with combined scar, runout and depositional zones. Since total areas with long runouts can cause large overestimates in subsequent volume derivations, corrections for runout are needed by estimating landslide scar areas^3^. This was achieved using the procedure of Marc et al.^69^. First, mass-wasting widths were calculated for each mapped feature using their perimeters, areas and the assumption that each feature can be approximated by an elliptical shape^66,69^. Second, assuming that mass-wasting scars have an aspect ratio of 1.5 (ref. ^70^) for a wide range of landslide sizes, scar areas can be calculated from *A*_s_ = 1.5 × *W*^2^, where *A*_s_ is scar area (m^2^) and *W* is feature to width (m).

Mass-wasting volumes were then estimated for both total areas and scar areas using the scaling relationships of Larsen et al.^29^, *V* = *α*.*A*^*γ*^, where *V* is the volume (m^3^), *A* is the area (m^2^), and *α* and *γ* are constant scaling parameters. For scar areas, appropriate values for *α* and *γ* reported by Larsen et al. are: *γ* = 1.262 ± 0.009 and log_10_*α* = −0.649 ± 0.021 for scar areas <10,000 m^2^ and *γ* = 1.41 ± 0.02 and log_10_*α* = −0.63 ± 0.06 for scar areas >10,000 m^2^. For total areas, we used the ‘all landslide’ parameters reported by Larsen et al.^29^, where *γ* = 1.332 ± 0.005 and log_10_*α* = −0.836 ± 0.015. Note that since these area–volume scaling relationships are designed for landslide events, they may overestimate the volumes of any remobilisations in our inventory, thus leading to potential overestimates in our overall ‘New + RR’ rate. However, any errors should be accommodated by the 20% error applied to all mapped features and thus unlikely to impact the overall results.

### Precipitation data

We use two precipitation products: PERSIANN-CDR and APHRODITE. The product properties and use justifications of these are outlined in the following paragraphs.

The PERSIANN Climate Data Record (CDR) product has a spatial resolution of 0.25° × 0.25° and temporal resolution of 3 h to 1 month over the period 1983–present^30^. This record is developed using the PERSIANN algorithm on GridSat-B1 IR satellite data. The algorithm is trained using hourly stage IV precipitation data from the National Centres for Environmental Prediction (NCEP) and then adjusted using the Global Precipitation Climatology Project (GPCP) dataset^30^. This product was selected as it is one of only a few accessible precipitation products with a spatial resolution of at least 0.25° × 0.25° that fully spans our time period of 1988–2018 (ref. ^31^). Daily precipitation totals (mm) for May–September were obtained from the CHRS data portal^71^ for our study region for all PERSIANN-CDR grid tiles that were at least 50% within our study region. Standard GIS tools were used to extract the various ASM strength metrics used.

PERSIANN-CDR is a widely used and comprehensively evaluated product (e.g. ref. ^72^). It was found to perform excellently when evaluated against 1400 ground stations for capturing the spatial and temporal patterns of rainfall in the monsoon-regions of eastern China^73^, and outperformed the TMPA (TRMM Multi-satellite Precipitation Analysis) dataset in its ability to capture the overall characteristics of Hurricane Catrina^72^. Furthermore, PERSIANN-CDR was found to have lower monthly mean variance compared to other satellite products, showing particularly small variance with the GPCP1DD product^74,75^. Similarly, despite being slightly outperformed by other products, the PERSIANN-CDR dataset was capable of capturing inter-annual monsoon precipitation in Pakistan, with high (0.8) R values when compared to in situ data^76^. However, PERSIANN-CDR has some limitations. First, as with all satellite products, it can struggle to capture orographic effects^77^. However, a benefit of PERSIANN-CDR is that it is designed specifically for use in longer-term studies^30,78^ and is considered one of the most temporally homogeneous products. Accordingly, unlike other satellite products whose methodologies could introduce temporal variance, any errors in the PERSIANN-CDR product introduced by orographic effects should be more systematic through time, and so not significantly bias our time series. This is important for this study, which requires a homogeneous rainfall series to ensure that any normalised perturbations are due to physical process changes, rather than changes in rainfall data collection methodology. Second, PERSIANN-CDR is reported to have a tendency to under-predict values of extreme precipitation^73,78^. Thus, to ensure that any under prediction of rainfall by PERSIANN-CDR does not impact our normalisation, and to allow for a more robust consideration of daily extreme precipitation, we also make use of the APHRODITE Asian Precipitation Highly Resolved Observational Data Integration Towards Evaluation of water resources) product^32^.

APHRODITE has the same spatial resolution as PERSIANN-CDR (0.25° × 0.25°) across monsoon-Asia, with daily coverage across the study region for 1951–2015. APHRODITE is based on rain gauge data from 5000 to 12,000 stations and is designed to optimise representation of orographic precipitation patterns. The temporal coverage of APHRODITE has advantages and disadvantages for this study. The disadvantage is that it does not allow us to assess the post-2015 earthquake preconditioning (a key aim of the study, and why the PERSIANN-CDR data are used to assess the entire time series). The advantage of the temporal coverage is that with a 64-year time series, robust analysis of extreme events and recurrence intervals are possible. APHRODITE is also considered as one of the most accurate products over the Himalaya^32,79^, making it a logical product to corroborate the results of our normalisation undertaken with PERSIANN-CDR. In summary, PERSIANN-CDR is used obtain a time-stable assessment of the entire time series, including the key post-2015 period (which APHRODITE cannot give without blending it with another dataset), while APHRODITE is used to corroborate the PERSIANN-CDR data and provide an unbiased comparison between the ASM strength analysis and extreme daily rainfall analysis.

### ASM strength-normalised mass-wasting rate

Empirical relationships between ASM strength and mass-wasting can be used to predict how much background mass-wasting is expected to occur each year based on that years ASM strength. Four previously investigated proxies of ASM strength^3,33^, for both the PERSIANN-CDR and APHRODITE data, were correlated with each measure of mass-wasting volume (total and scar volumes (m^3^/km^2^) of new and reactivated/remobilised landslides [‘New + RR’] and of only new landslides [‘New Only’]). These were total grid-averaged MJJAS precipitation, total grid-averaged MJJAS precipitation >25 mm, total grid-averaged precipitation from 15 June to September, and total grid-averaged precipitation >25 mm for 15 June–September. The ASM strength proxies which provided the best fit to the mass-wasting data were total MJJAS rainfall for the PERSIANN-CDR data, and total MJJAS rainfall >0.25 mm for the APHRODITE data (see Fig. 2 for best-fit results and Supplementary Figs. S1–S3 for all other correlations).

For each measure, the ASM strength-normalised rate of each year is then calculated by taking the ratio of the actual mass-wasting mapped for that year to that predicted by the equations in Fig. 2. A value of one indicates that the actual observed mass-wasting in a year is what would be expected given the ASM strength, while a value significantly above one indicates that there was more mass-wasting than expected given the ASM strength, implying perturbation above the background by some other event. Errors in the normalised rate include the standard error in the data points used to calculate the prediction equations, an assumed standard deviation of 20% in mass-wasting area to account for variability in mapping period and any mapping error, and the standard deviations reported in the area–volume conversion parameters. Assuming that these errors are uncorrelated, they were combined using standard Gaussian propagation of error to obtain the uncertainties for each measure (Fig. 3a, b).

Furthermore, prior to correlating mass-wasting with ASM strength, we removed the largest landslide from a given monsoon season if it had a scar-volume twice as large as the second largest landslide^3^. This approach is designed to remove any landslides that are anomalously large for the monsoon season in which they occurred, and thus likely caused by progressive failure across multiple monsoon seasons (e.g., the Jure landslide^3,80^). By removing these events, we can be confident that any identified perturbations are not due to a single anomalously large landslide. Accordingly, 12 events were removed from the analysis, one event in each of 1988, 1996, 2000, 2003, 2004, 2005, 2014 and 2017, and two events in both 2009 and 2015.

### Three-parameter inverse-gamma distributions

To further confirm that the identified perturbations are not due to stochastic variation in landslide size, we fit three-parameter inverse-gamma distributions to the PDF of landslide area for several subsets of our inventory (all years, all pre-2015 non-perturbed years, 1989, 1993, 1995, 2002, 2015 and 2016–2018). The PDF of landslide area *p* (*A*_L_) is given by Eq. (1)^43^:

1 $$p({A}_{\mathrm L})=\frac{1}{{N}_{\mathrm {LT}}}\frac{\partial {N}_{\mathrm L}}{\partial {A}_{\mathrm L}}$$

where *N*_LT_ is the total number of landslides in the subset, *A*_L_ is landslide area, *δN*_L_ is the number of landslides with areas between *A*_L_ and *A*_L_ + *δA*_L_. The three-parameter inverse-gamma distribution fitted to the PDFs is defined by Eq. (2)^43^:

2 $${{\mathrm {pdf}}}({{A}}_{l}|\alpha ,\eta ,\lambda )=\left[\frac{{\lambda }^{2\alpha }}{\varGamma (\alpha )}\right]\left[{\left(\frac{1}{x+{\eta }^{2}}\right)}^{(\alpha +1)}\right]\exp \left[-\frac{{\lambda }^{2}}{x+{\eta }^{2}}\right]$$

where *α* controls the exponent of the inverse power law (i.e., the steepness of the right tail of the PDF), *η* controls the steepness, or bend, of the left tail of the PDF, and *λ* controls the position of the rollover. The position of the rollover indicates the landslide area below which the inverse power law decay observed for medium and larger landslides no longer applies. The PDFs and three-parameter inverse-gamma distribution were fitted to each subset using the LAMPRE software^81^, which utilises Maximum Likelihood Estimation (MLE) to optimise the parameters of the PDF and a bootstrapped (here with 1000 simulations) Kolmogorov–Smirnov (K–S) test to estimate parameter uncertainty and overall goodness of fit of the inventory data to the fitted distribution.

The exponent, *α*, of the inverse power law describes the rate at which the probability of getting larger landslides decreases. A larger exponent indicates that the probability of getting larger events is decreasing quickly, and thus that larger landslides are contributing less to each inventory. Conversely, a smaller exponent indicates that the probability of getting larger events is decreasing more slowly, and thus that larger landslides are contributing more to each inventory. Thus, if the exponents of the distributions fitted to each subset are similar above comparable cut-offs, then we can be confident that a perturbation is caused by some physical process that causes an increase in landslides of all sizes, rather than a small number of anomalously large landslide events.

Note that our rollover values (see main text) are larger than the values obtained for the Gorkha coseismic landslides (2500 m^2^)^24^ and previously mapped monsoon-triggered landslides (1200 m^2^)^3^. This is likely because of differences in mapping resolution, with the minimum possible size feature that could be mapped for the coseismic and previous mapped landslides an order of magnitude smaller than could be mapped here^3,24^. However, our rollover values are comparable to similar studies using imagery with 30–15 m resolution imagery^82^, suggesting that our inventory is as substantially complete as would be expected given the resolution of the satellite imagery. Our scaling exponents are also slightly smaller than the value of ~2.47 obtained for higher resolution inventories of both monsoon-triggered and earthquake-triggered landslides in Nepal^3,24^. Again, this is likely an artefact of imagery resolution, and that we are under-sampling the smallest events.

### Percentage change in mass-wasting

To calculate the percentage change in 2015 mass-wasting, we divided the study region into 84 grid cells (Fig. 6). For each grid cell, we calculated the mean mass-wasting (based on scar volumes) observed across all unperturbed monsoon seasons (i.e., all years except 1988, 1989, 1993, 1995, 2002 and 2015). We then calculated the percentage change in 2015 monsoon-triggered mass-wasting for each grid relative to that grid’s mean. By only calculating each cell’s average with the non-perturbed years, we obtain an approximation of average mass-wasting expected per grid in a typical monsoon season without extreme rainfall. This will not consider monsoonal forcing in the detail it was on the regional scale. However, since we know that 2015 was not impacted by any extreme rainfall, each grid’s 2015 percentage change in monsoon-triggered mass-wasting should approximately reflect the ‘above average’ or excess mass-wasting experienced in 2015 due to the earthquake compared to an average non-perturbed monsoon season. The same method was used to assess the percentage change in mass-wasting in 2002 and 2004.

### Excess topography

Excess topography, a measure of the total volume of rock mass above a specified threshold hillslope angle^49^, was extracted from the Japanese Aerospace Exploration Agency (JAXA)-copyrighted ALOS World 3D DEM using the ‘excesstopography’ function in the Matlab TopoToolbox^83^. Excess topography was calculated at five threshold angles: 25°, 30°, 35°, 40° and 45°. The average excess topography at each threshold across each grid cell was then extracted using standard ArcGIS zonal statistics tools.

### Reporting summary

Further information on research design is available in the Nature Research Reporting Summary linked to this article.

## Supplementary information

Supplementary Information

Peer Review File

Reporting Summary

Description of Additional Supplementary Files

Supplementary Data1

## Data Availability

The raw mass-wasting data used within this manuscript are provided as a.text file (Supplementary Data 1) that includes the geometries (areas, volumes), centroid coordinates and satellite data used to map each individual feature. A polygon shapefile of the landslide inventory on which these data are derived can be freely accessed from the National Geoscience Data Centre (NGDC) repository (item ID 166966): https://webapps.bgs.ac.uk/services/ngdc/accessions/index.html?simpleText=landslide%20nepal#item166966.

## Code availability

This manuscript includes no custom code or algorithms.
