# Supplementary material for: 30-year record of Himalaya mass-wasting reveals landscape perturbations by extreme events
Source: Nat Commun. 2021 Nov 18;12:6701. doi: 10.1038/s41467-021-26964-8 (PMC8602672; doi:10.1038/s41467-021-26964-8)
Supplement: Supplementary file 1 — Supplementary Information [file 41467_2021_26964_MOESM1_ESM.pdf]

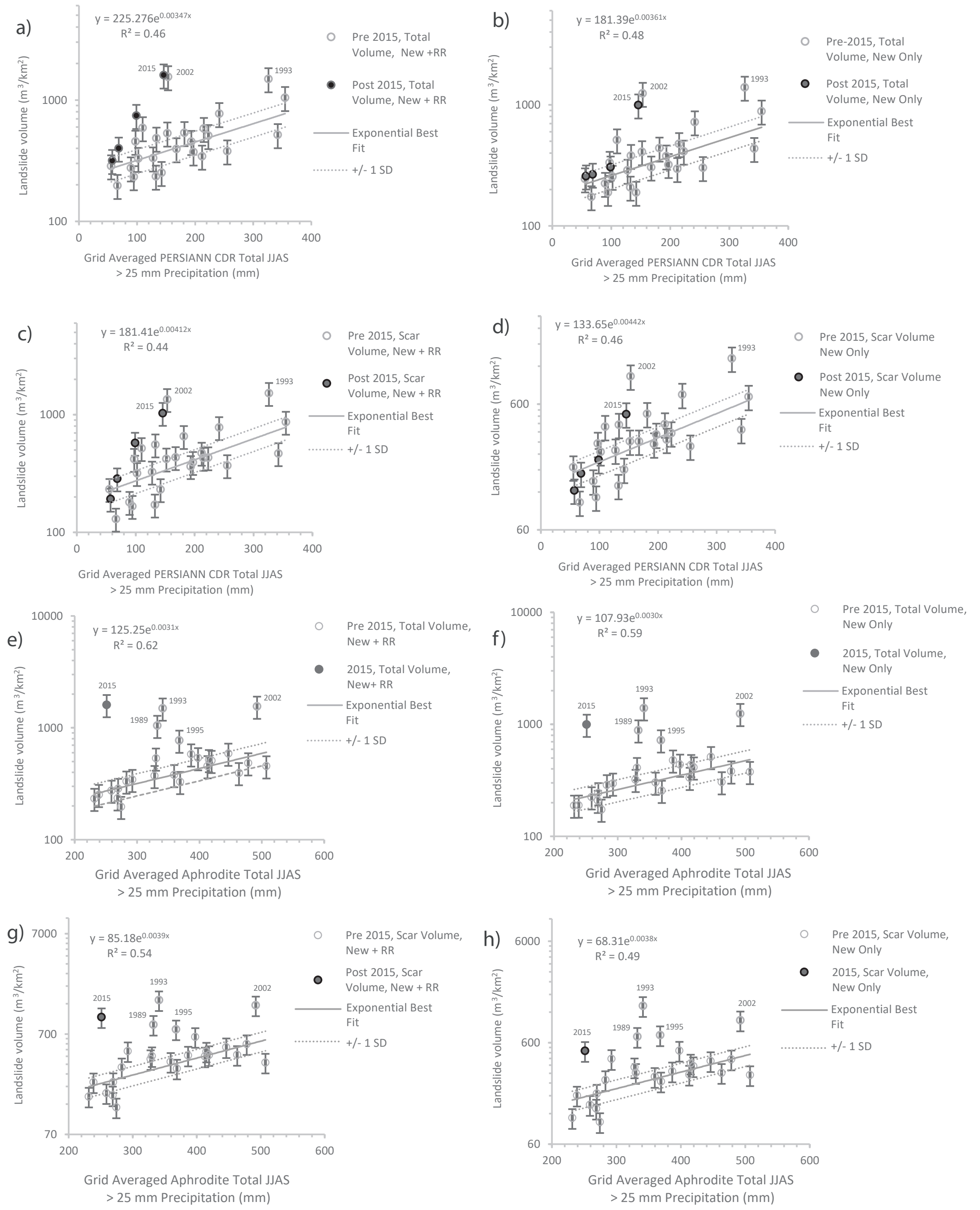

Figure S1. a – d) Empirical relationships between measures of mass-wasting volume (m³/km²) and PERSIANN-CDR total MJJAS precipitation > 25 mm for a) total “New + RR” volume, b) total “New Only” volume, c) scar “New + RR” volume and d) scar “New Only” volume. e – h) Empirical relationships between measures of mass-wasting volume (m³/km²) and APHRODITE total 15th June - Sept precipitation > 25 mm for e) total “New + RR” volume, f) total “New Only” volume, g) scar “New + RR” volume and h) scar “New Only” volume. Where, in all cases “New + RR” refers to the combined volumes of both new failures and reactivations/remobilisations and “New Only” refers to just the volumes of new failures, with reactivations and remobilisations excluded. The exponential best fits shown on these graphs apply to the non-anomalous pre-2015 points only, with all anomalous points labelled individually. The post-2015 points are also shown for reference, as are the +/- 1 standard errors on the fit equations.

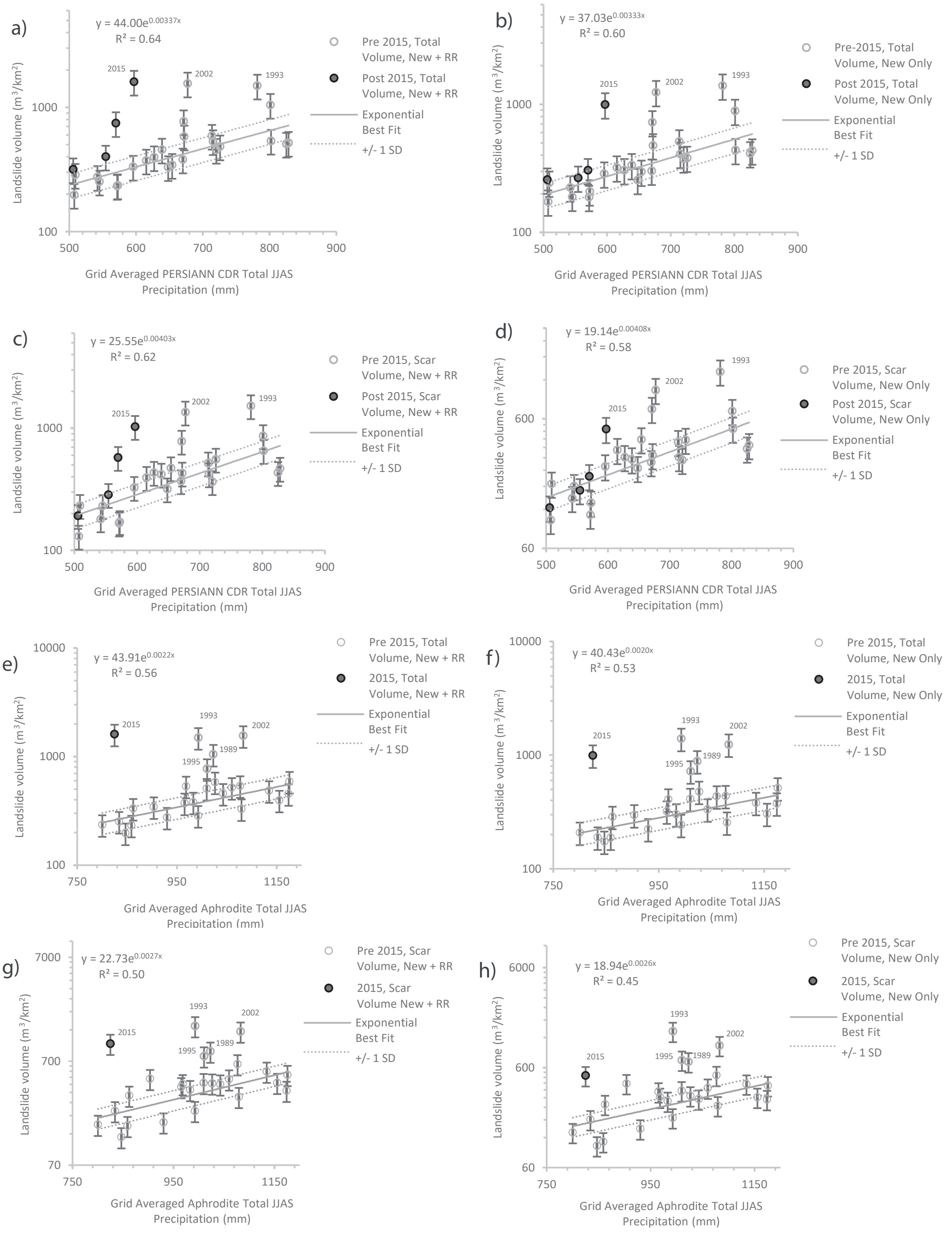

Figure S2. a – d) Empirical relationships between measures of mass-wasting volume (m^3/km^2) and PERSIANN-CDR total 15th June - September precipitation for a) total "New + RR" volume, b) total "New Only" volume, c) scar "New + RR" volume and d) scar "New Only" volume. e – h) Empirical relationships between measures of mass-wasting volume (m^3/km^2) and APHRODITE total 15th June – September precipitation for e) total "New + RR" volume, f) total "New Only" volume, g) scar "New + RR" volume and h) scar "New Only" volume. Where, in all cases "New + RR" refers to the combined volumes of both new failures and reactivations/remobilisations and "New Only" refers to just the volumes of new failures, with reactivations and remobilisations excluded. The exponential best fits shown on these graphs apply to the non-anomalous pre-2015 points only, with all anomalous points labelled individually. The post-2015 points are also shown for reference, as are the +/- 1 standard errors on the fit equations.

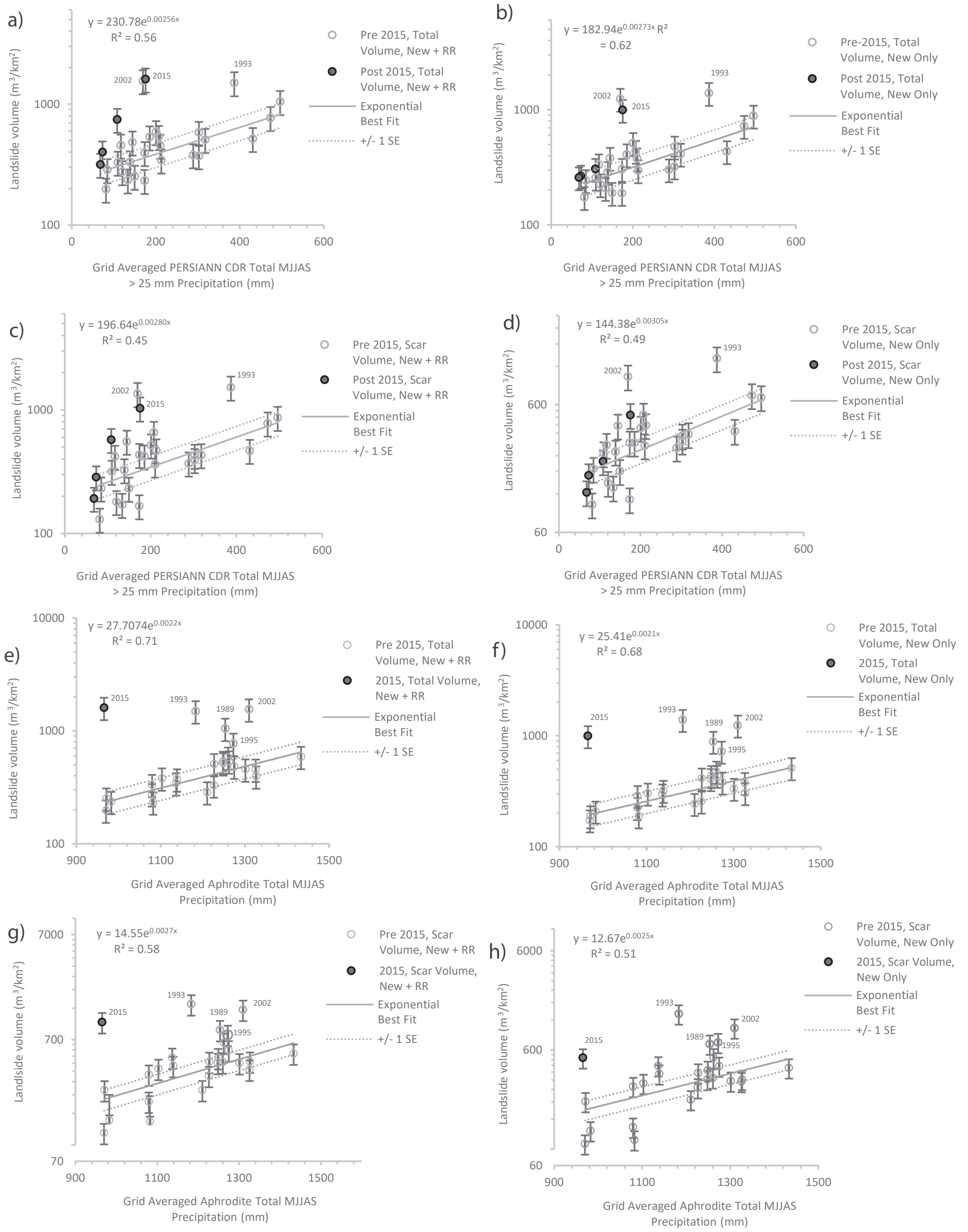

Figure S3. a – d) Empirical relationships between measures of mass-wasting volume (m^3/km^2) and PERSIANN-CDR total MJJAS > 25 mm precipitation for a) total "New + RR" volume, b) total "New Only" volume, c) scar "New + RR" volume and d) scar "New Only" volume. e – h) Empirical relationships between measures of mass-wasting volume (m^3/km^2) and APHRODITE total MJJAS precipitation for e) total "New + RR" volume, f) total "New Only" volume, g) scar "New + RR" volume and h) scar "New Only" volume. Where, in all cases "New + RR" refers to the combined volumes of both enw failures and reactivations/remobilisations and "New Only" refers to just the volumes of new failures, with reactivations and remobilisations excluded. The exponential best fits shown on these graphs apply to the non-anomalous pre-2015 points only, with all anomalous points labelled individually. The post-2015 points are also shown for reference, as are the +/- 1 standard errors on the fit equations.

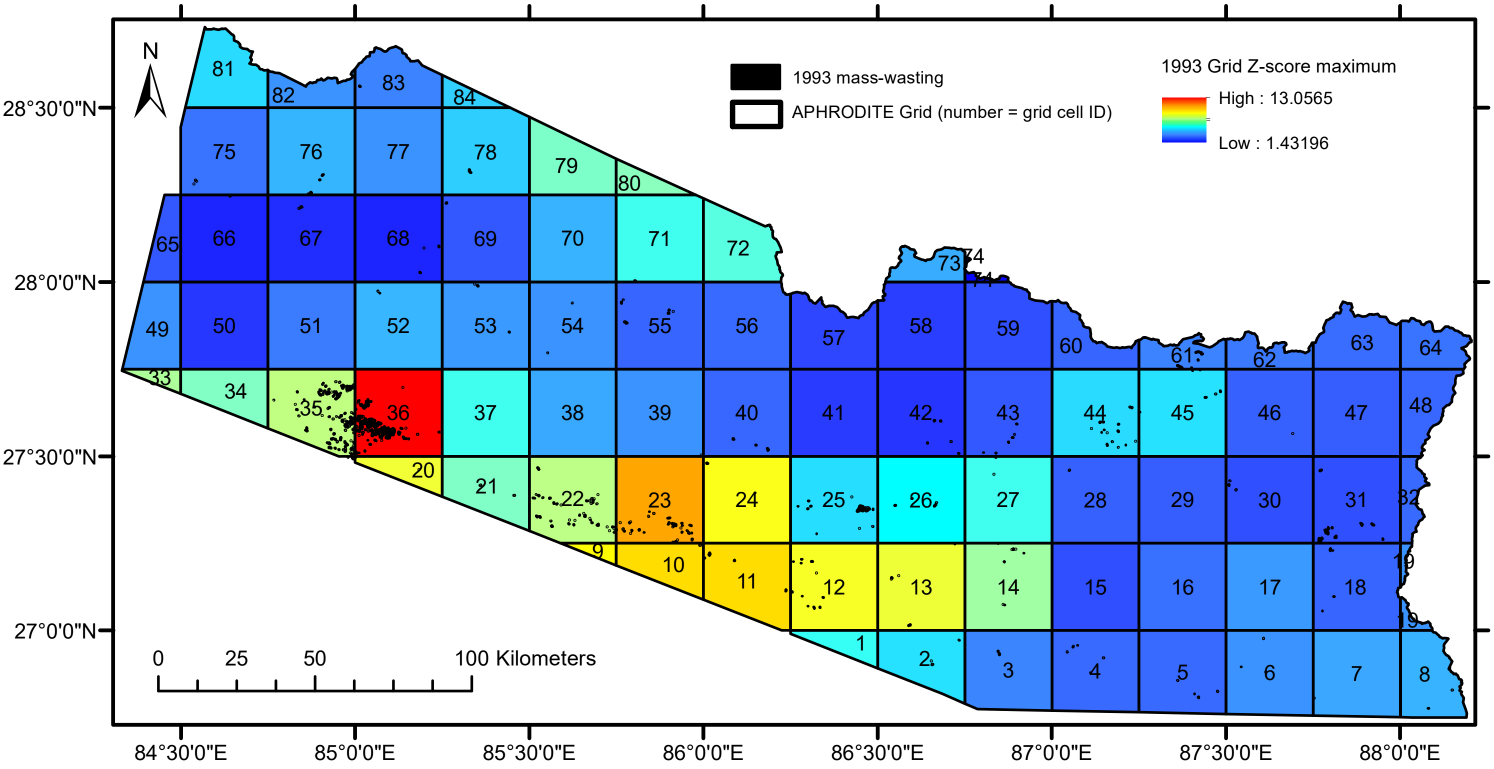

Figure S4. Map showing the 1993 Z-score anomaly and associated mass-wasting.

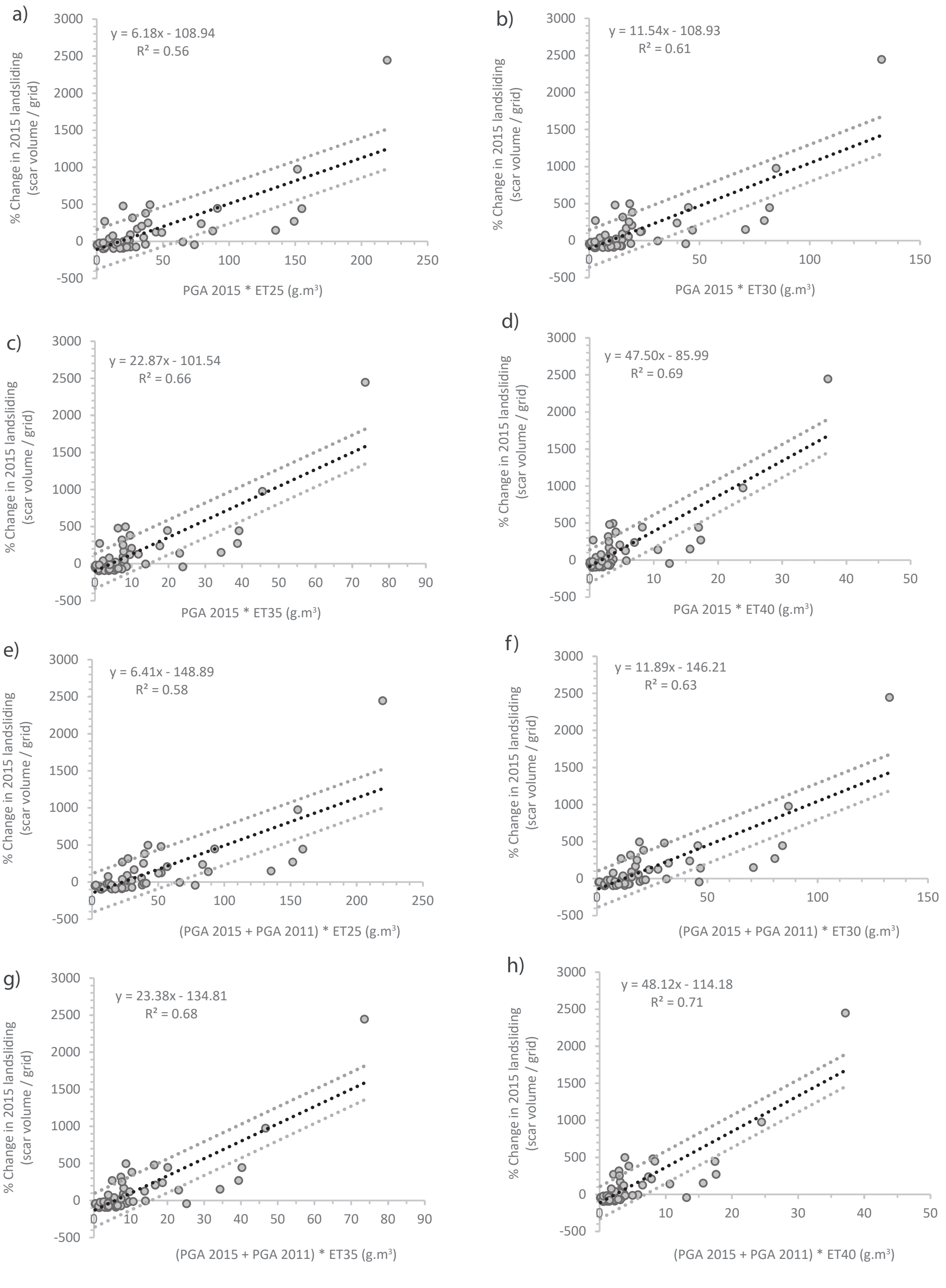

Figure S5. Correlations between excess monsoon-triggered mass-wasting in 2015 and maximum summed PGA in the 2015 main shock and largest aftershock multiplied by excess topography above a threshold angles of a) 25°, b) 30°, c) 35° and d) 40°. e – h) Correlations as in a – d) but with the PGA from the 2011 earthquake included in the summed PGA. The linear best-fits are shown with +/- 1 standard error in each case.

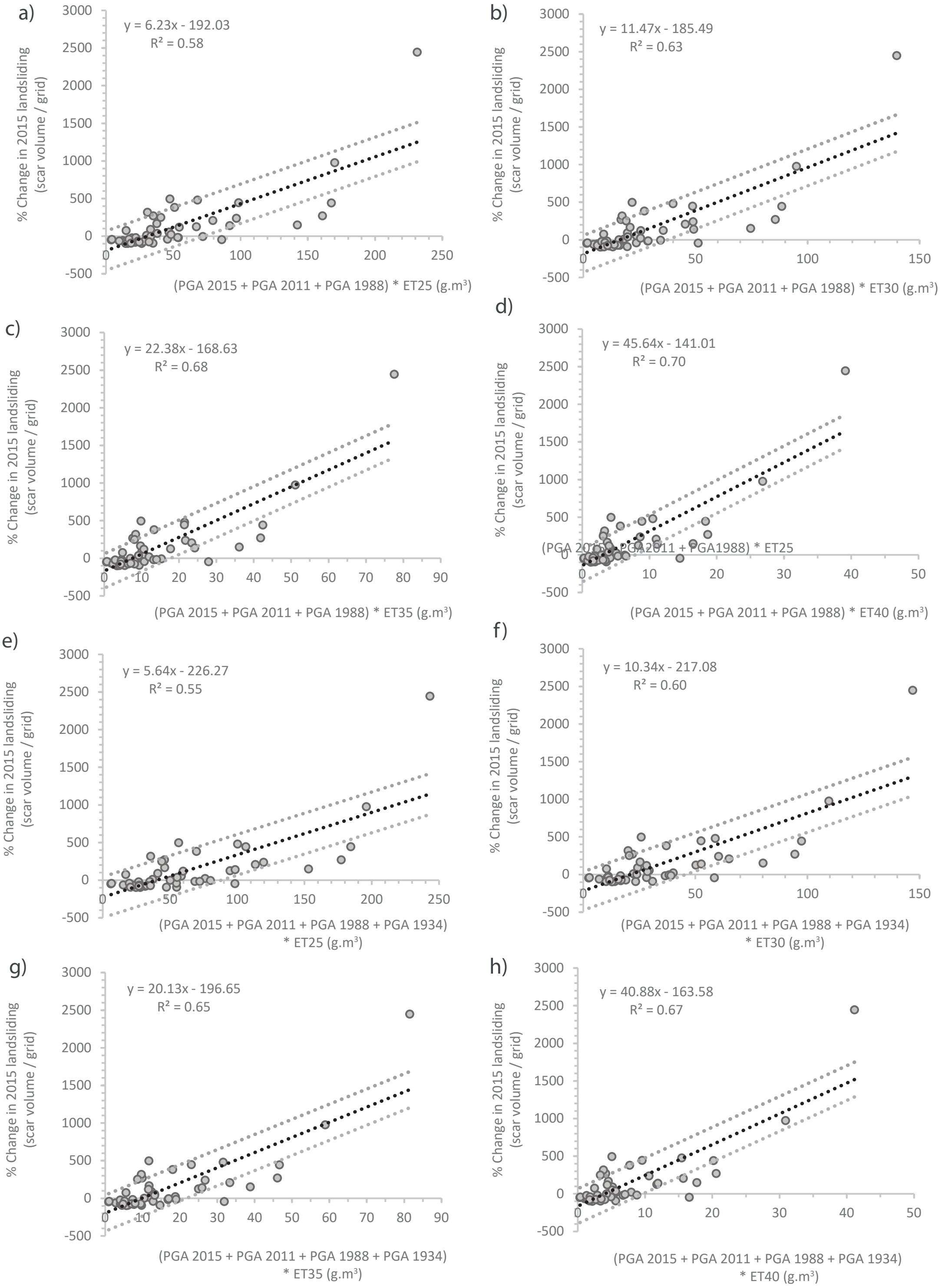

Figure S6. Correlations between excess monsoon-triggered mass-wasting in 2015 and maximum summed PGA in the 2015 main shock, 2015 largest aftershock, 2011 earthquake and 1988 earthquake multiplied by excess topography above a threshold angles of a) 25°, b) 30°, c) 35° and d) 40°. e – h) Correlations as in a – d) but with the PGA from the 1934 earthquake included in the summed PGA. The linear best-fits are shown with +/- 1 standard error in each case.

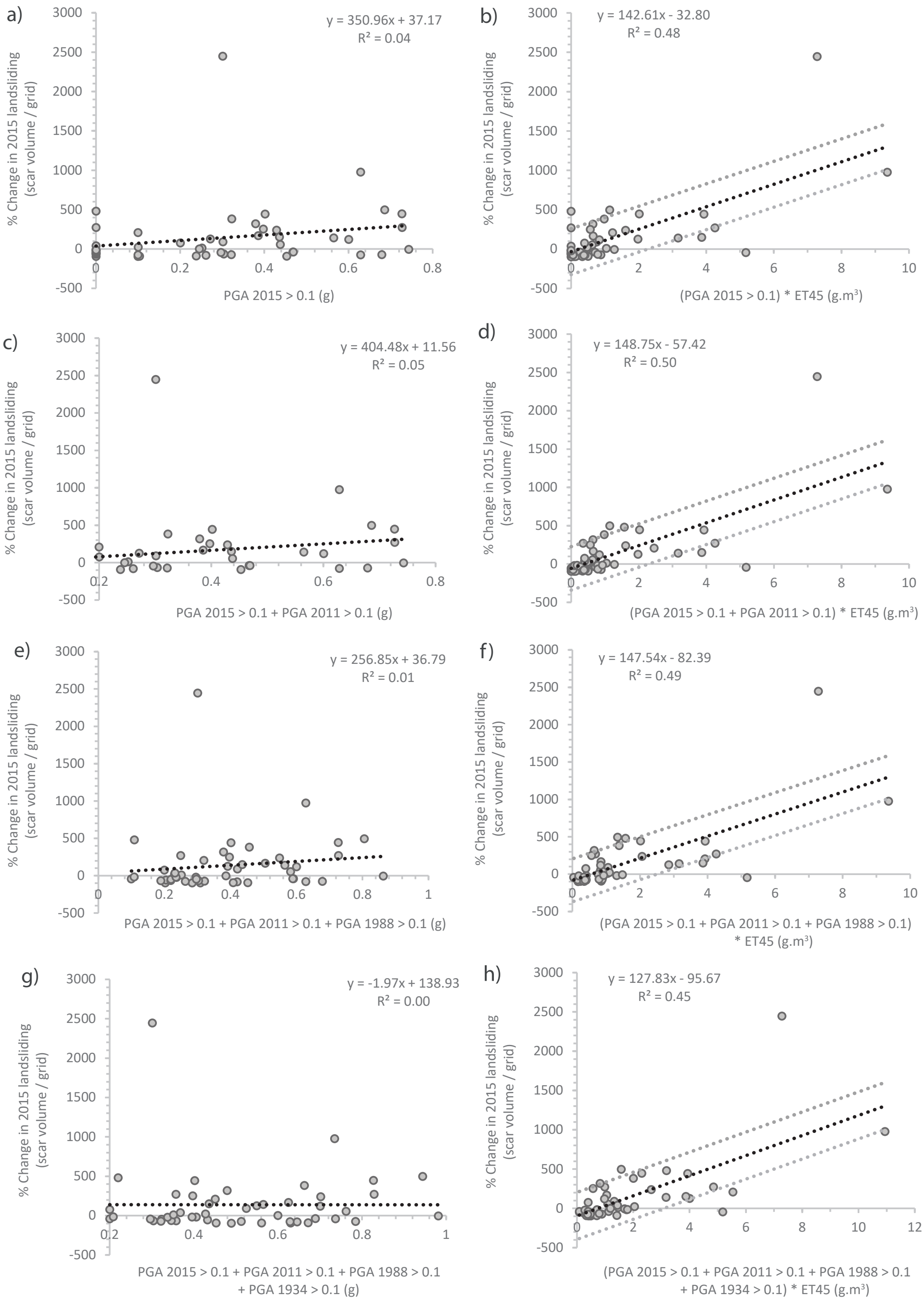

Figure S7. Correlations between excess monsoon-triggered mass-wasting in 2015 and summed PGA > 0.1 g in a) the 2015 main aftershock and largest aftershock, b) as a) but plus the 2011 PGA > 0.1 g, c) as b) but plus the 1988 PGA > 0.1 g, and d) as in c) but plus the 1934 PGA > 0.1 g. e – h) the same correlations in a – d) but with PGA multiplied by excess topography above a threshold angle of 45°.

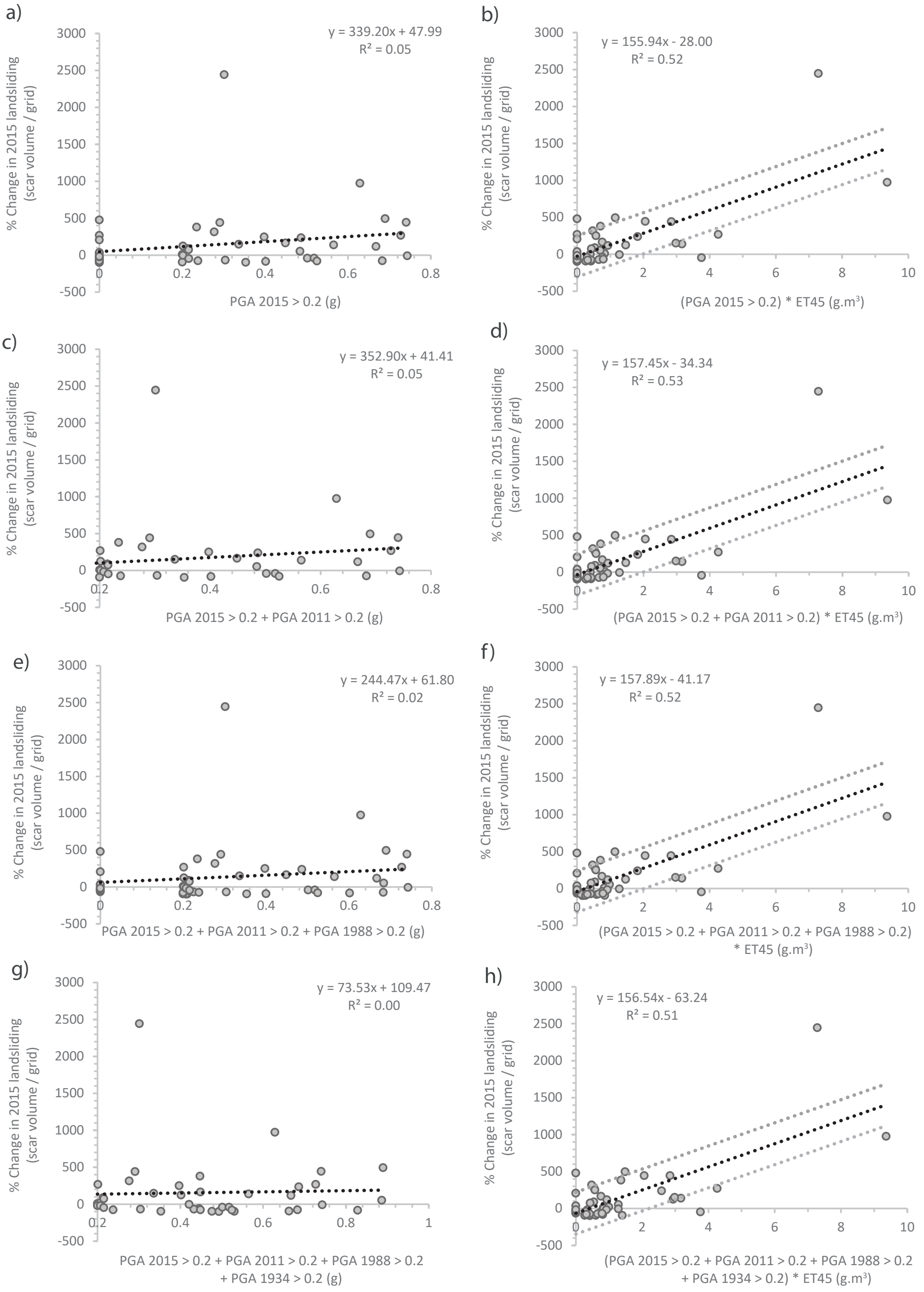

Figure S8. Correlations between excess monsoon-triggered mass-wasting in 2015 and summed PGA > 0.2 g in a) the 2015 main aftershock and largest aftershock, b) as in a) but plus the 2011 PGA > 0.2 g, c) as in b) but plus the 1988 PGA > 0.2 g, and d) as in c) but plus the 1934 PGA > 0.2 g. e – h) the same correlations in a – d) but with PGA multiplied by excess topography above a threshold angle of 45°.

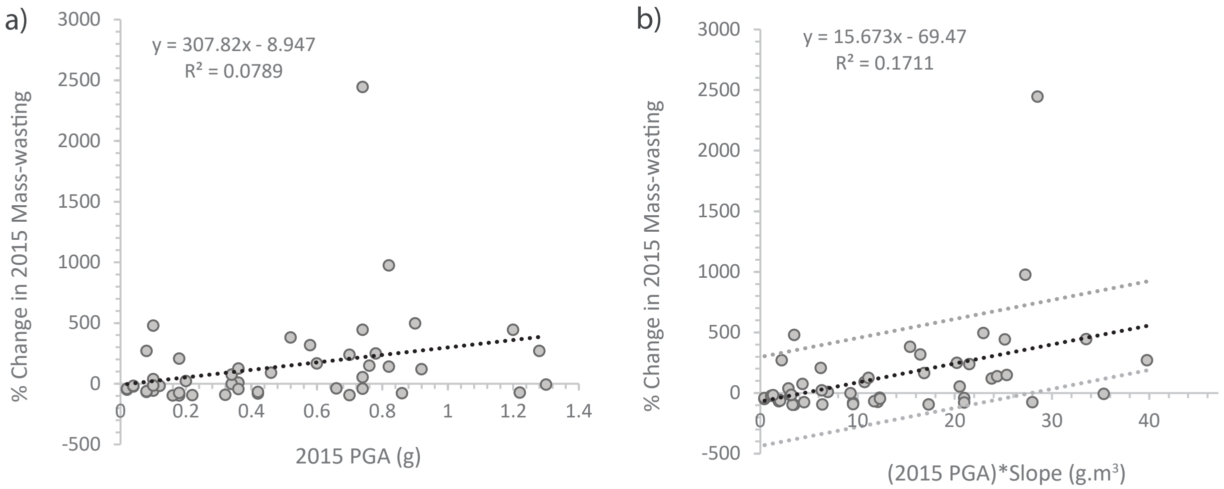

Figure S8. Correlations between excess monsoon-triggered mass-wasting in 2015 and a) maximum summed PGA in the 2015 main shock and largest aftershock, and b) maximum summed PGA in the 2015 main shock and largest aftershock multiplied by average slope angle.

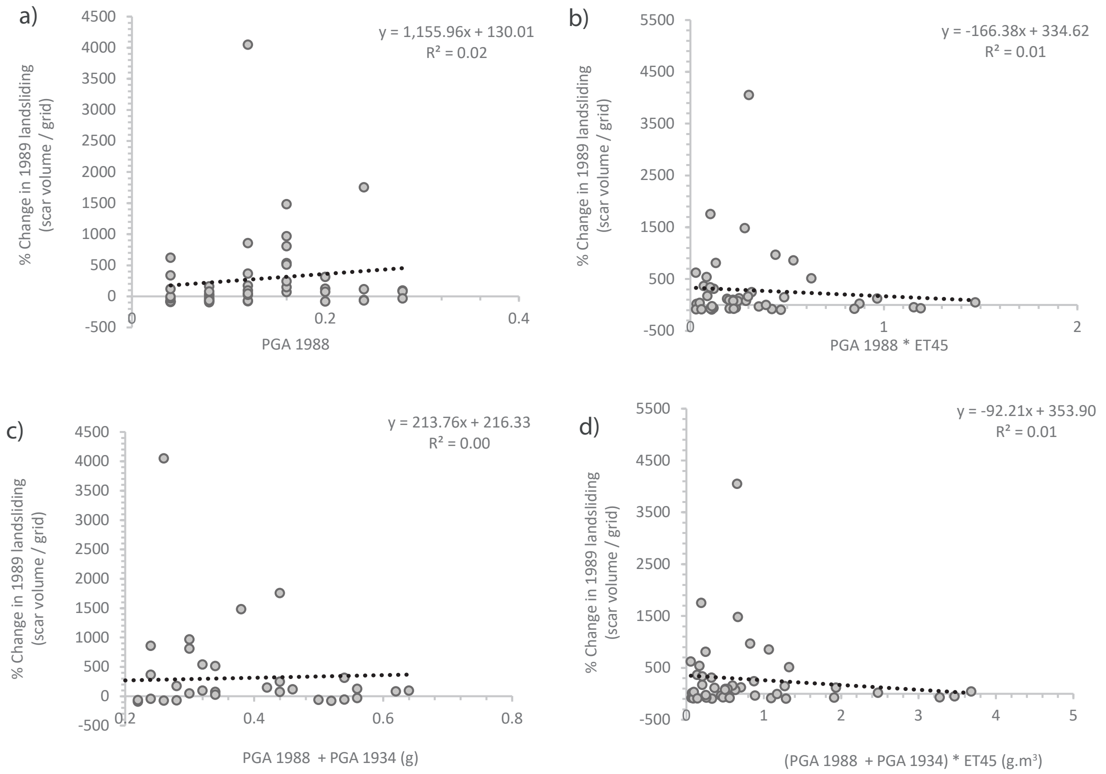

Figure S10. Correlations between excess monsoon-triggered mass-wasting in 1989 and summed PGA in a) the 1988 earthquake, b) the 1988 earthquake multiplied by excess topography above a threshold angle of 450, c) the 1988 and 1934 earthquake, d) the 1988 and 1934 earthquakes multiplied by excess topography above a threshold angle of 45°.

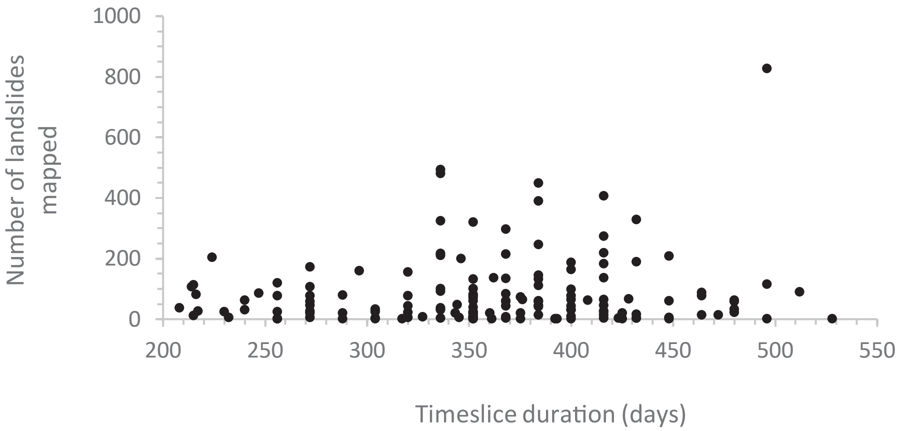

Figure S11 – Correlation between time slice length (where variation is due to varying numbers of days between October and April) and number of mapped features. We find no positive relationship between the two, suggesting that very few events occur between October and April, and thus that our varying time slice lengths do not unduly affect our analyses.

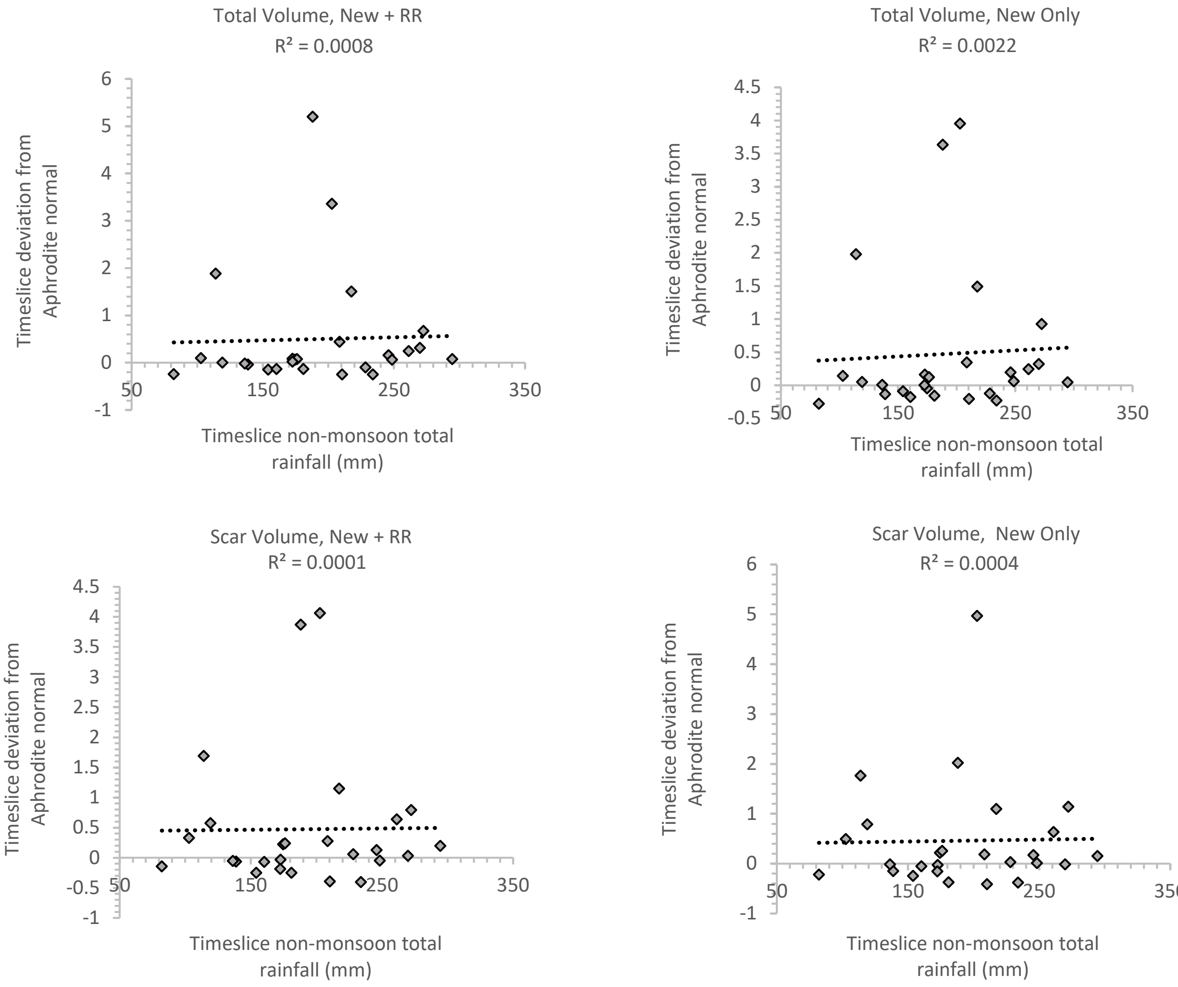

Figure S12 – Correlation between total rainfall in the non-monsoon months included in each mapping interval and the deviations from the normal n the normalised rate observed in figure 3b. We find no positive relationship between the two, suggesting that our varying time slice lengths do not unduly affect our analyses.

Year	No. Mapped Features	Total Volume "New + RR" (m^3)	Total Volume "New Only" (m^3)	Scar Volume "New + RR" (m^3)	Scar Volume "New Only" (m^3)	Satellite Product Used
1988	552	23842587	20327357	25329600	21316385	Landsat 4/5
1989	368	44606067	37795615	36977800	29307496	Landsat 4/5
1990	282	24798168	20356572	18248563	13367633	Landsat 4/5
1991	185	14664730	12655041	20171382	17717302	Landsat 4/5
1992	206	10757394	8060680	9882284	7721032	Landsat 4/5
1993	688	63706490	59524933	64963866	59052172	Landsat 4/5
1994	239	15881316	13668377	16828875	14655775	Landsat 4/5
1995	329	32881528	30801027	33287489	30403043	Landsat 4/5
1996	349	17878160	14024401	20811952	15219065	Landsat 4/5
1997	248	16196123	12896086	15801173	11798940	Landsat 4/5
1998	274	20637304	16252455	23724167	17550177	Landsat 4/5
1999	369	25149652	21853231	22080466	16885497	Landsat 4/5
2000	477	19763192	14557902	18940784	13471883	Landsat 7
2001	572	22742863	17444836	17995094	12919774	Landsat 7
2002	1337	66201168	52972260	57674509	42572127	Landsat 7
2003	297	19987080	16671775	17856598	14606480	Landsat 7
2004	564	22259342	18215714	20338658	16930196	Landsat 4/5
2005	149	9962131	8952169	7263236	5938146	Landsat 4/5
2006	206	14173187	12255139	13929565	10967058	Landsat 4/5
2007	211	22935697	18688172	27965378	21391510	Landsat 4/5
2008	216	12195684	10402270	9918266	8057466	Landsat 4/5
2009	175	11456406	9351355	9609328	6937638	Landsat 4/5
2010	310	14054953	10916087	13546765	10640480	Landsat 4/5
2013	433	9931421	8042592	7118385	4642711	Landsat 8
2014	507	20382998	18199991	25598549	24139158	Landsat 8
2015	1328	79809974	42454871	61102244	21285238	Landsat 8
2016	890	31772872	13029709	24516651	9213868	Landsat 8
2017	753	18691302	12983404	14089973	9062546	Landsat 8
2018	406	13507735	10991457	8200926	5270255	Landsat 8

Table S1 Summary of yearly mass-wasting data
