# Supplementary material for: 30-year record of Himalaya mass-wasting reveals landscape perturbations by extreme events
Source: Nat Commun. 2021 Nov 18;12:6701. doi: 10.1038/s41467-021-26964-8 (PMC8602672; doi:10.1038/s41467-021-26964-8)
Supplement: Supplementary file 4 — Description of Additional Supplementary Files [file 41467_2021_26964_MOESM4_ESM.pdf]

Description of Additional Supplementary Files

Supplementary Data 1

Point-data of our mass-wasting inventory, including the monsoon year, perimeter, total area (combined scar, runnout and deposition zones), centroid coordinates
scar area, total volume and estimated scar volume of all mapped features. The type and date of the satellite imagery used to map each feature are also included (Pre-Sat and Post_Sat).
