# Supplementary material for: 30-year record of Himalaya mass-wasting reveals landscape perturbations by extreme events
Source: Nat Commun. 2021 Nov 18;12:6701. doi: 10.1038/s41467-021-26964-8 (PMC8602672; doi:10.1038/s41467-021-26964-8)
Supplement: Supplementary file 2 — Peer Review File [file 41467_2021_26964_MOESM2_ESM.pdf]

nature portfolio

Peer Review File

30-year record of Himalaya mass-wasting reveals landscape perturbations by extreme events

Open Access This file is licensed under a Creative Commons Attribution 4.0 International License, which permits use, sharing, adaptation, distribution and reproduction in any medium or format, as long as you give appropriate credit to the original author(s) and the source, provide a link to the Creative Commons license, and indicate if changes were made. In the cases where the authors are anonymous, such as is the case for the reports of anonymous peer reviewers, author attribution should be to 'Anonymous Referee' followed by a clear attribution to the source work. The images or other third party material in this file are included in the article's Creative Commons license, unless indicated otherwise in a credit line to the material. If material is not included in the article's Creative Commons license and your intended use is not permitted by statutory regulation or exceeds the permitted use, you will need to obtain permission directly from the copyright holder. To view a copy of this license, visit http://creativecommons.org/licenses/by/4.0/.

REVIEWER COMMENTS

Reviewer #2 (Remarks to the Author):

Review of Perturbations to Himalaya mass-wasting by rainfall, earthquakes and road building.

Jones et al., Nature Comms.

The paper attempts to explore the association between Himalayan mass wasting to monsoon strength, road construction and extreme storms. In this respect this is a timely piece of research that tackles a topic of wide interest and relevance. The paper focuses upon the generation of a new time series of mass wasting events mapped across a large portion of Nepal over a period of 29 years, and then seeks to explain variations away from the long-term average rate of mass wasting. The generation of this time series is an exciting prospect as there is potential to feed into wider understanding of landsliding in the Himalayas, understanding of landscape evolution and hazard and risk.

Whilst this is an ambitious intention, I remain concerned by the issues identified below that relate to both the presentation of the manuscript, but more fundamentally to the underlying research. Based upon this the conclusions and the headline message around which the paper is framed appears problematic in several aspects.

My comments below are intended to strengthen this work in future and bring attention to some key issues which I my view should be addressed prior to considering this research for publication, and I note that addressing these to support the same conclusions represents a significant effort. It may well be that some of the suggestions are not feasible, but if this is the case, the aim and conclusions of the paper should be modified to reflect this.

I provide some general comments about what has been undertaken, rather than focussing on detailed line by line comments at this stage.

Methods:
• Overall the methods are not well explained (I don't think it would be possible to replicate what has been done based on the detail provided), and much remain ambiguous and undefined. Some of the comments below may arise from this lack of clarity.
• The mass wasting time-series is collated by using a pair of images to identify each feature from before and after each monsoon over a 29-year period. By definition, the mapping therefore only intends to capture mass wasting that occurs within the monsoon period only, and not total change from one year to the next. The decision to do this is presumably to isolate the monsoon impact, which is fine, but this has consequences for other analyses that can be conducted with this data. The clarity of this distinction is lost throughout the manuscript which is currently presented as the net contribution of a range of factors that relate both to the monsoon (ASM), but also potentially to the period outside of the monsoon that is not consistently mapped (which potentially includes EQs, road construction and extreme rainfall). As a result, without very careful explanation, the results as presented seem to be quite misleading. There are some important details that arise as a result, which I pick up on below.
• Images have been selected to bracket the monsoon period as tightly as possible, with the date range for pre- and post- monsoon images stated as latest end April and earliest end October respectively. This sampling raises several questions and some implicit assumptions which are not explained in the results that are generated…
• On the basis of the description in the manuscript, the mapping period for each feature could cover a shortest period in any year between 30 April and 31 October of 182 days (best case), or a longest period of 550 days (worst case). I note from the supplementary data that the actual mapping period (newest image date minus oldest image date in pair used to identify each feature) has a shortest range of 208 days, and the longest range of 528 days. Critically therefore, some mass wasting

features are identified from across a period that is double that of other features. If all mass wasting occurs only in the monsoon this might be OK, but we don't know this and it probably doesn't – specifically that associated with factors other than the monsoon, so this is a problem. This is an issue that I consider further below.

• Moreover, by summarising the average mapping interval by year from the Supp Data, there is considerable systematic variation in the mapping periods between years (minimum average mapping interval = 225 days (2015), maximum = 457 days (2007)), which again is double. The data from some years is therefore implicitly covering a different time period and so may be systematically over- or under-representing mass wasting that is unevenly distribution through the year. The explanation of the method does not suggest that the variable mapping period is normalised in the analysis that follows, and even if it was this wouldn't fully account for this inconsistency.

• There is also considerable variation in the period over which individual features have been mapped within each individual year of the dataset (e.g. 32-day range between longest and shortest periods in 2006, to a 272-day range in 1995. This is a > 8-fold difference between years, which again raises issues for comparison and for identification of longer-term trends that really need to be considered.

• Some important related issues arise here. First, the comparison through time relies on very consistent data, but it remains unclear how this variability in period over which data has been collected is considered, and the manner in which associated temporal uncertainty in the timing of mass wasting is accounted for. Second, there is an implicit assumption here that the majority of the mass wasting under consideration occurs within the monsoon only, and from the above, for some years mass wasting occurring only in ca. 7 months of the year is mapped. Whilst this may well be true for ASM-triggered mass wasting, the same cannot be said for EQs, road construction and potentially extreme rainfall events (see below). How are mass wasting events outside of the monsoon considered and how to they feature in the overall calculations of relative contribution? The manner in which the data appears to have been collated implies that this will, unfortunately, not be possible to resolve….

Completeness of data:

• A second significant issue arises from the mapping methodology, and the completeness of the data generated. The numbers of landslides mapped in each year are very low compared to other published datasets for this region and period, particularly for the Gorkha earthquake. Whilst I appreciate the difficulties in comparing datasets from different mapping techniques and imagery, the closest comparable effort appears to be that reported in Regmi et al (2016) which is also based primarily on LandSat data. For a smaller area, they map ca. 4,000 landslides, as compared to only 1,400 landslides in a far bigger area in this paper.

• This apparent difference extends to a comparison of the aerial extent of landslides (this paper identifies ca. 17.6 km2 of mass wasting, Roback identifies ca. 87 km2 for 2015 in > 20k events). These differences are surprising given the stated minimum mapped landslide size is small and more comparable to other efforts (300 m2 – although this in itself seems very small, given Landsat pixel sizes….).

• Related, I also note the discussion of the power-law fitting, and the lowest size thresholds identified at around 11,000 m2. One implication of this threshold is that below this size, the inventory is incomplete. Therefore, the completeness of the dataset needs to be fully evaluated – as at the moment the manuscript is very unclear on this and there are stark differences to other research. As this completeness is absolutely fundamental to your calculations of relative impacts on mass wasting from different controls, this needs addressing.

• As in previous papers, there is a focus here only on new landslides, rather than any form of reactivations. The justification for this is a little vague, but has implications for the results that are generated: "We also removed landslide reactivations from this second "corrected" volume of mass-wasting. This is because we define earthquake preconditioning, a key process for investigation in this study, as a post-earthquake increase in new ASM-triggered mass-wasting, which would not include reactivations of coseismic or past ASM-triggered deposits." Despite this, it remains true that an EQ might trigger a very small part of a much bigger eventual landslide that fails under rainfall, but this would not be included in this analysis, despite the fact that this event would not have occurred without the earthquake. Without fully evaluating the relative contribution of reactivations versus new

landslides, I don’t think you can just exclude these, particularly given the ambition here for time-dependent susceptibility assessments. From a hazard perspective, this is also problematic – a farmer in Nepal doesn’t care if he is hit by a new landslide or a reactivation – it is still happening because of the earthquake and subsequent rainfall… This choice again adds a small but critical subtlety to the simple summary figures that are the headline of this paper, which I feel are problematic without proper explanation.

Non-stationary baseline conditions:

• An overarching concern is the very simplified view of the conditions that are potential controls on mass wasting in this part of the Himalayas during the study period. Critically, the baseline conditions are far from stationary. The period considered has experienced considerable social, political and economic change which remains highly challenging to disentangle from impacts on mass wasting. The period covers significant outmigration of working age males – with a well-documented impact on the degradation of agricultural land, the civil conflict – which dramatically impacted farming practise, the changes in the planning process – which influences when and where developments such as road construction takes place, significant urbanisation, plus much much more… The processes upon which you focus (roads, ASM, EQs, extreme rainfall) are playing out upon this backdrop, and I would argue cannot be separated from this without a considerably higher resolution and precision of data. I don’t therefore believe that it is possible to ‘isolate’ the influence of the chosen factors as is suggested here. In the simplest sense this issue is illustrated in the calculation of the long-term average mass wasting rate – which by definition assumes zero change in background conditions by over the 30 years – when these long-term changes may each be a significant and interrelated catalyst in promoting mass wasting.

• Related to this, the word ‘correlation’ is in my view used incorrectly throughout the manuscript – without direct data on the controls rather than just mass wasting on its own, this analysis does not really go beyond identifying ‘coincidence’.

Assessment of roads:

• The primary impact of roads on mass wasting in the Himalaya is from rural or seasonal roads constructed with minimal engineering input. Rural roads (ca. 2 – 3 m in width, unsurfaced and commonly with minimal spectral contrast to the surrounding landscape) are arguably invisible – or at best inconsistently visible – in LandSat imagery (15 m, 30 m). Evidence to the contrary needs to be provided if this is how this has been done… I am therefore unclear on the confidence with which the association between roads and landslides can really be made when the roads must be barely visible in the imagery used here. The method to identify this association is also not well explained so this analysis remains unconvincing.

• The only fact presented about roads is the timing of a World Bank funded program – this seems a far too simplistic association to imply in the absence of any data on roads, particularly given the far wider range of donor funded road and rural access programs that have rolled out during this study period, in addition to the wider set of influences on road construction in Nepal (see above, and below).

• A key further consideration is that rural road construction in Nepal is widely acknowledged to be a highly seasonal activity, very unevenly distributed through the year. Where the analysis conducted here only samples part of the year (and inconsistently so…), the risk of not capturing the impact of road construction in a uniform manner is therefore high. There are several important related issues for the analysis undertaken: (1) the timing of road construction is closely linked to both the fiscal year and the annual planning cycle in Nepal – with a common rush in road construction towards the end of the former. This research needs to consider how completely road construction impacts are captured if a net impact is to be derived – but I am not clear that the present data can actually do this; (2) The planning process for road construction has changed significantly during the study period considered here – from the 14 step planning process (up until 2017) and the 7 step process thereafter – this significantly influences when roads are built, so there is potential for a shift in bias through the time period considered; (3) related, the budgeting structure and decision making for rural road construction has also changed significantly during this period, with a notable localisation of construction capacity and decision making. A consequence is that when the mapping technique covers the monsoon period

only, road construction – and the associated landslides – will potentially not be mapped, which may well be a significant number, and given then the annual date of imagery used in the mapping varies year on year, therefore the period being sampled varies. If road construction was uniformly distributed through the year this might be considered negligible, but it is not, and so this is potentially very important. This variability in road building activity needs to be considered and a demonstration of its role made… Without actual data on road construction, I don't see how this can be achieved, and so this represents a considerable extra effort.

• There is also an implicit assumption that road construction has an instantaneous impact on mass wasting and no longer-term legacy effect, which is surprising given the argument for time-dependent landslide susceptibility. So, I don't agree with this statement: "However, as road-associated mass-wasting is not related to rainfall, a second "corrected" volume of mass-wasting was calculated with this mass-wasting type removed". Road construction can prime a slope to fail if it does not immediately trigger collapse (in the same manner as the EQ preconditioning ideas) – but rainfall remains a key recurrent trigger. In this sense, without more detailed data, it remains very difficult to separate rainfall and road impacts. This really needs thinking through in more detail and supporting with analysis…

Consideration of earthquakes:

• The selection of potentially landslide triggering earthquakes has a weak justification. The 30 km radius for inclusion is arbitrary, and more fundamentally using epicentre is a very crude approach to identifying which events might be important when we know that the spatial distribution of PGA or PGV is what controls landsliding. It is impossible to associate the pattern of PGA or PGV to the epicentre location alone. If the analysis were to consider for example the extension of the 1g isoseismal into the AOI for all earthquakes during the mapping period, I would not be surprised to see the 1988 and 2011 earthquakes included. This really needs to be considered, and if no impact on mass wasting is observed for these events, then why is this the case?

• The paper considers to some extent the ideas around the legacy effects of previous earthquake damage in conditioning subsequent mass wasting, although doesn't really get into much detail on this idea. Research on this topic is in its infancy, but some key issues seem to be overlooked here. The legacy damage effect of earthquakes is not just experienced in the transient response immediately (1 – 5 years) after the initial shaking. For example, the Parker et al paper referenced identifies damage legacy effects manifest over intervals of ca. 40 years for Mw7.7 and & 7.1 events in NZ. With this in mind, it is surprising here not to see any consideration of the potential legacy of historic and very much larger earthquakes which have directly impacted the AOI (in addition to 1988, 2011, also 1934, 1833, 1505 and 1255…).

Consideration of extreme rainfall:

• I'm very surprised given the 30-year period, the extent of the study, and the local climate, that only two extreme rainfall events have been identified as having an influence on mass wasting. This potentially shows the relatively insensitivity of the approach described. I note that the perturbations are identified and then a driver is sought, rather than looking at drivers and trying to identify if a mass wasting response occurred…this seems a slightly circular approach. Given that there is no data on extreme rainfall considered here to assess which events have consequences, and which don't, by only considering two events this analysis does not seem comprehensive enough to really evaluate the net impacts of extreme rainfall events.

Other key comments:

• I am uncomfortable about relying on 'using the methods described by Marc et al. 21', - this paper is not a good example of clarity of explanation and I would strongly suggest the methods used here are fully explained to avoid perpetuating ambiguity.

• The choice of rainfall product really needs to be justified and evaluated (e.g. see Ullah et al., 2018, Remote Sensing). For example, as the rainfall data has ca. 28 km grid size, how does this compare to

storm size and how does it influence total monsoon rainfall calculations in an AOI with a significant precipitation gradient, and how does the averaging in the derivation of this product, and in your subsequent treatment of the data behave over mountain topography?

• The paper makes claims around showing a link between ASM and mass wasting, and hence hazard. Given this, it is not clear why no attempt to compare the outputs to more widely used monsoon strength indices is made (e.g. SASMI). I note the Petley et al (2007) paper reference conducts a comparable analysis for fatal landslides in Nepal with SASMI, and identifies what on the face of it is the inverse to the relationship you show, but does go on to offer some explanations of this. This is important as 'monsoon strength' means different things to different people – and most will read this as measures such as SASMI, rather than a bespoke Nepal specific strength measure.

• The LandSat derived background mass wasting rate is compared to the data from Roback to assess the relative impact of the Gorkha earthquake. As far as I can understand from the explanation, this appears problematic because (1) the Roback data is much higher resolution, is collected over an area that is entirely impacted by the earthquake, and is data collected from imagery over < 60 days (these factors all derive a very high event mass wasting rate), but (2) the data that this is compared to presented here is collected over a far bigger area where only a small portion is directly impacted by the Gorkha earthquake, the resolution and completeness is at least an order of magnitude lower, and the time period of mapping is much wider. All of these factors will accentuate the difference between the short and long term mass wasting rates. This just does not appear a valid comparison to make. It is not clear why the data mapped here for 2015 is not used here, or does the relatively limited number of mapped features inhibit this…?

• This point above also raises a question about how to compare rates between the controls identified. For the extreme rainfall, a local average rate is derived and compared to one specific to that event. This seems different for how the comparison is undertaken for Gorkha, where rates for the whole area impacted are considered. As it is known (and shown in Figure 1) that landsliding varies considerably across this area over the long term, how then is the impact of roads handled – is this a deviation in local mass wasting rate for the location of the road only, or a net change in rate for the whole area? When comparing events and controls, how each is treated needs to be consistent, which does not appear to be the case at the moment…Again this has a big influence on the simple summary stats….

Figures:
• Figure: 1 – no admin boundaries make this difficult to orient. DEM data has no source / attribution. DEM scale is odd – seems to be reporting a new highest place on earth! Surely the max elevation is 8,848 m, not >11,000 m?
• Figure: 2 – confidence intervals would be useful here, particularly given the variability on the log axis.
• Figure: 3 – it would seem sensible to only show EQs of sufficient magnitude to trigger landsliding (e.g. 6Mw…) not all.

Supplementary data:
• The mapped shapefiles would be more widely useful than the *.txt file of summary stats.
• I would also like to see the full inventory posted to this repository for wider scrutiny and for use in other research: https://www.sciencebase.gov/catalog/item/586d824ce4b0f5ce109fc9a6

Reviewer #3 (Remarks to the Author):

Review of Jones et al “Perturbations to Himalaya mass-wasting by rainfall, earthquakes and road building”
Oliver Francis

This is an interesting and in-depth study into the mass wasting processes of the Himalaya. The authors use an extensive landslide inventory and precipitation data to estimate the contributions of different processes to mass wasting. The authors produce the first empirical relationship between total monsoon rainfall and mass wasting and use this to identify 4 perturbations to the average mass wasting events. Further they identify mass wasting associated with road building and identify it as a growing contributor to erosion rates. I believe this are novel and important findings which further our understanding of the erosion of the Himalaya.

I have several comments concerning the methodology and findings of the paper and several smaller suggestions for improving the clarity of the manuscript. I will go through the major comments first and provide a list of the smaller comments with line numbers afterwards.

Major comments

The work presented here in this manuscript quantifies the volumes of mass wasting produced by different processes across a 30 year time period and argues these are meaningful for understanding long term erosion rates and forecasting changes due to climate change or road construction. However, without an understanding of the frequency of these processes it is impossible to convert these volumes into rates and put them into context of time scales longer than the 30 years recorded here. I recommend identifying the return period of the extreme rainfall events in 1993 and 2002 and comparing those with the frequency of Gorkha Earthquake in order to fully understand their relative contributions to erosion rates.

Along a similar line I also found the quantification of the contribution of road construction to mass wasting confusing. It is unclear in the manuscript how these mass wasting events are identified, are they simply the volume of material produced during the construction? Or do they include the enhanced mass wasting associated with undercutting of slopes and poor slope reinforcement? Separating these two processes is important for understanding the continued contribution of road construction to mass wasting after the study period. It is also unclear how the recorded areas are converted to volumes; these events are not landslides so using a landslide area – volume scaling is not appropriate. Volumes of material produced by the construction of the roads could be estimated by measuring the width of the roads and the heights of their cuttings into the hillslope. Accurate estimations of the mass wasting produced by construction and post construction along with road mapping would allow forecasting of mass wasting of future construction projects.

Finally, while acknowledging the limits of the format of the journal, I feel that several extra figures (listed below) are required in order to help the reader interpret the findings and the methodology of the paper.
Minor comments

Line 54: I find the definition of the “background” rate confusing. Is the background rate simply the estimated rates of mass wasting from the monsoons? Or does it also include storm induced mass wasting outside of the monsoon months? Clarifying this definition along with the timing of the monsoon will help to better explain the aim of the paper.
Line 64: Cosmogonic should be cosmogenic
Line 79: The definition of mass wasting associated with road building is confusing. Is the sediment produced by cutting and constructing the road recorded or is it the enhancement due to undercutting of slopes? The former is a single event while the later is a continuing process. Figures of examples of road associated mass wasting would help with these definitions
Line 112: ~ should be >

Line 112: The magnitude-frequencies of the mapped mass wasting events should be included as a figure to help the reader understand their statistics better, particularly as their shape is used in the methodology
Line 134: How is the strength of a monsoon defined? Strength could be total precipitation or intensity or another measure of precipitation. I would avoid the word strength and be more specific
Line 138: I found the current wording of the methodology confusing. As it is currently worded it sounds like a single mean is produced of the monsoon landsliding rather than a yearly estimate. I suggest rephrasing it to: "each of the 29 ASM seasons."
Line 153: A figure of the yearly frequency – magnitudes would help the reader interpret and understand these findings themselves.
Line 161: If a monsoon can produce mass wasting significantly greater than the estimated value from the normalisation it raises the question of how well the normalisation works. If this storm is as anomalous as is implied by the normalisation the manuscript should justify why it is not included in the normalisation. This would include discussion on how to identify extreme events, particularly as they seem to be exponential rather than normally distributed in nature.
Line 167: The use of landslide density is confusing and hides the fact that the 2002 storm was more erosive than the 1997 storm. I suggest presenting all findings in terms of volume rather than area.
Line 169: To combine these two events I feel the return time of the rainstorms needs to be understood. Without knowing the return period of the storms, it is unclear whether these events are frequent, and therefore important to the long-term erosion rate or insignificant. Combining them together could make them seem more significant than they are.
Line 175: Preconditioning is set up to be an important part of the manuscript however very little time is spent on describing it in this study. A figure showing the described correlation between earthquake magnitude and preconditioning (particularly as no mention of preconditioning prior to 2015 despite multiple >Mw6 earthquakes) would help to give more attention to this process.
Line 177: The timing of the earthquake compared to the monsoon is not clear. It should be noted somewhere that the earthquake occurred prior to the monsoon. It is also unclear whether the coseismic landslides were mapped or the Roback 2018 inventory was used.
Line 178: It is not clear why preconditioning increases mass wasting by a factor of 4 but is only the same as 2 ASMs. This should be rewritten for clarity
Line 187: Reference to figures
Line 193: Again, reference to figures
Line 196: If this were true surely the uncorrected trends would not decrease through time? They maintain a similar trend to the corrected i.e. decreasing to the background rate which implies that preconditioning is important here too
Line 197: The areas shown in figure 4 cannot be compared to volume therefore it is hard to compare with the other perturbations discussed
Line 201: It would be useful to know something about the amount of road construction occurring. What is the correlation between rate of road building and mass wasting? This would help clarify the definition of road associated mass wasting and forecasting into the future. Figures of images showing examples of mass wasting associated with road construction will also help to clarify how they are related and defined.
Line 202: As area is shown in figure 4 it is now not clear whether volume or area is discussed here. I again recommend only using volume when discussing or show results.
Line 207: A figure showing the absolute values of mass wasting would be useful to show how the contribution by each process changes through time.
Line 236: You have not identified the controls on these events, are these years of intense monsoon that are poorly predicted by the normalisation method or are they freak storms of abnormal intensity? It is also possible similar storms occurred without causing significant mass wasting, without any analysis on rainfall patterns in this period it is difficult to interpret these events. Without clear definitions of extreme events and their return periods it is difficult to determine whether these events are likely to change through time.
Line 261: The timing of the monsoon should be mentioned to help the reader understand why these months were chosen for imaging

Line 300: This section should be above the section discussing precipitation as it links directly to the discussion of mass wasting.
Line 343: The averaging of the monsoon should be using volume rather than area. Area is not a useful measure of sediment flux due to the relationship between landslide area and volume.
Line 370: Here it would be useful to have a figure of the magnitude – frequencies of the years described. The reader can then made informed decisions on the applicability of this methodology.
Figure 2: It would be good to have the events discussed in the manuscript highlighted in this figure. Some form of time series of precipitation would also help to understand the extreme rainfall event described.
Figure 4: The "Reactivations + Road" line is hard to see. Area is difficult to compare with volume as discussed in the rest of the manuscript. Area is not important for erosion rates and should not be compared. Absolute volume values through time would be much more useful.

Reviewer #2 (Remarks to the Author):

Anonymous

Comment(s) 1:

My comments below are intended to strengthen this work in future and bring attention to some key issues which I my view should be addressed prior to considering this research for publication, and I note that addressing these to support the same conclusions represents a significant effort. It may well be that some of the suggestions are not feasible, but if this is the case, the aim and conclusions of the paper should be modified to reflect this.

• Overall the methods are not well explained (I don't think it would be possible to replicate what has been done based on the detail provided), and much remain ambiguous and undefined. Some of the comments below may arise from this lack of clarity.

Response(s) 1:

As implied by the reviewer, many of the key issues raised by this review are in part due to misunderstanding of our methods and data completeness. As such, we believe that many of the issues raised, which could have required significant extra effort or not been feasible in the timeframe, are in fact not such big issues at all. Consequently, we believe that the vast majority of our initial aims and conclusions can be supported. The exception to this is our analysis and results pertaining to roads. As we outline in several places below, we have come to the conclusion that, despite our road-tipping data being temporally continuous, we do not have sufficient supporting data to analyse and assess road-tipping impacts in a comparable manner to the ASM, extreme rainfall and earthquakes. As such, we have decided to remove the road-tipping elements of this manuscript, which we believe makes the narrative of the publication more concise and impactful, and allows us to focus on what are, in our opinion, some very novel and impactful results on earthquake preconditioning.

Comment 2:

The mass wasting time-series is collated by using a pair of images to identify each feature from before and after each monsoon over a 29-year period. By definition, the mapping therefore only intends to capture mass wasting that occurs within the monsoon period only, and not total change from one year to the next. The decision to do this is presumably to isolate the monsoon impact, which is fine, but this has consequences for other analyses that can be conducted with this data. The clarity of this distinction is lost throughout the manuscript which is currently presented as the net contribution of a range of factors that relate both to the monsoon (ASM), but also potentially to the period outside of the monsoon that is not consistently mapped (which potentially includes EQs, road construction and extreme rainfall). As a result, without very careful explanation, the results as presented seem to be quite

misleading. There are some important details that arise as a result, which I pick up on below.

Response 2:

This is not correct, and is a misunderstanding arising from a lack of clarity on our part. Our mapping is continuous, with the post imagery used to map one year almost always used as the pre-imagery for the next year. As is now described in lines 99 – 103:

"Mapping was conducted across 29 individual time slices, where each time slice encompassed a given year's monsoon season (approx. May – September) as well as a varying number of months either side (see methods). Our inventory is continuous, with the post-imagery used to map one time slice typically used as the pre-imagery for the following time slice."

As such, our mapping does not only capture the monsoon, instead capturing all mass-wasting across the mapped period, with each time slice approximately cantered on the monsoon season.

Comment(s) 3:

• Images have been selected to bracket the monsoon period as tightly as possible, with the date range for pre- and post- monsoon images stated as latest end April and earliest end October respectively. This sampling raises several questions and some implicit assumptions which are not explained in the results that are generated…

• On the basis of the description in the manuscript, the mapping period for each feature could cover a shortest period in any year between 30 April and 31 October of 182 days (best case), or a longest period of 550 days (worst case). I note from the supplementary data that the actual mapping period (newest image date minus oldest image date in pair used to identify each feature) has a shortest range of 208 days, and the longest range of 528 days. Critically therefore, some mass wasting features are identified from across a period that is double that of other features. If all mass wasting occurs only in the monsoon this might be OK, but we don't know this and it probably doesn't – specifically that associated with factors other than the monsoon, so this is a problem. This is an issue that I consider further below.

• Moreover, by summarising the average mapping interval by year from the Supp Data, there is considerable systematic variation in the mapping periods between years (minimum average mapping interval = 225 days (2015), maximum = 457 days (2007)), which again is double. The data from some years is therefore implicitly covering a different time period and so may be systematically over- or under-representing mass wasting that is unevenly distribution through the year. The explanation of the method does not suggest that the variable mapping period is normalised in the analysis that follows, and even if it was this wouldn't fully account for this inconsistency.

• There is also considerable variation in the period over which individual features have been mapped within each individual year of the dataset (e.g. 32-day range between longest and shortest periods in

2006, to a 272-day range in 1995. This is a > 8-fold difference between years, which again raises issues for comparison and for identification of longer-term trends that really need to be considered.

• Some important related issues arise here. First, the comparison through time relies on very consistent data, but it remains unclear how this variability in period over which data has been collected is considered, and the manner in which associated temporal uncertainty in the timing of mass wasting is accounted for. Second, there is an implicit assumption here that the majority of the mass wasting under consideration occurs within the monsoon only, and from the above, for some years mass wasting occurring only in ca. 7 months of the year is mapped. Whilst this may well be true for ASM-triggered mass wasting, the same cannot be said for EQs, road construction and potentially extreme rainfall events (see below). How are mass wasting events outside of the monsoon considered and how to they feature in the overall calculations of relative contribution? The manner in which the data appears to have been collated implies that this will, unfortunately, not be possible to resolve….

Response(s) 3:

We have grouped these comments together as they concern the same issue around the mapping of our mass-wasting database. As outlined above, the mapping was continuous and our revised methodology sections now make this clear.

However, we do agree that the impacts of time-slice length could have been considered. Our analysis pertaining to the ASM and extreme rainfall assumes that the majority of rainfall-triggered mass-wasting will occur between May and Sept, when Nepal is known to experience the vast majority of its rainfall. Therefore, as the reviewer themselves outlines, our varying time slice length will not be a problem if it's true that most mass-wasting occurs during the monsoon-period, which is always regularly included within each time slice. Indeed, the mapping period variation will only be a problem if the months October – April see significant mass-wasting. Our assumption that most mass-wasting occurs between May - September is backed up by the literature (see Petley et al., 2007 and Stanley et al., 2020) and by sensitivity analysis we have now conducted, which shows no correlation between time-slice length (i.e. number of October – April months included in a time slice) and mass-wasting occurrence (see supp materials; fig. 2).

To further confirm that the number of Oct – April months included will not impact our results, we went back and re-mapped several extra time-slices between October and April to see how many rainfall-triggered events actually occurred in these periods. These extra time slices were: November 1988 to March 1989 (6 events occurred), September 1998 to January 1999 (4 events occurred), November 2005 to April 2006 (9 events occurred), October 1996 to December 1996 (0 events occurred) and October 1997 to November 1997 (0 events occurred). In all of these cases, the number of events to occur was below 10% (in all but the Nov05 – Apr06 case below 5%) of the total mass-wasting of the main time slices these periods were included in. I.e. the mass-wasting in the Oct – April months is well within the 20% assumed mapping error we applied to all of our calculations. As such, in the case of rainfall-triggered events (i.e. the ASM and extreme rainfall) our sensitivity analysis suggests the variable

number of Oct – April months included in each time slice will not impact the results, and any variability due to this should be within the 20% error applied to our calculations.

As mentioned above we have removed roads from this analysis.

Discussion / description on the above is now included in the methods section of the manuscript for clarity, lines 487 – 507:

"Each time slice included a given year's monsoon season (May – September) plus a varying number of months either side. The variation in the number of October – April months included in each time slice was an unavoidable consequence of the high levels of cloud cover across the Himalayas. However, as our time slices had varying lengths, both between time slices and within time slices (as several tiles were required to map the entire study region, and invariably these tiles had different acquisition dates and cloud cover), it is necessary to consider the effect of this on the subsequent analysis. Our analysis of ASM-triggered and extreme rainfall triggered mass-wasting assumes that all mass-wasting was triggered during a given time slice's monsoon season. As these time slices include months outside of the monsoon period, it is possible that some of these rainfall-triggered events did not occur during the monsoon. However, it is known that this region experiences very little rainfall-triggered landsliding outside of the monsoon period [6,42]. Indeed, we find that there is no correlation between number of non-monsoon months within a time slice and number of mass-wasting events mapped (Supplementary materials, Fig. 2). This suggests that, as expected, the majority of rainfall triggered events can be assumed to have occurred during the monsoon season, and thus that variable time slice length will not unduly impact the results. Furthermore, to ensure that this variability in time slice length does not impact the results, we applied a 20% assumed error to all mapped mass-wasting areas. This assumed error should account for any variability in mapped mass-wasting caused by including non-monsoon months, as well as for any inadvertently included mass-wasting events that are attributable to non-rainfall dominated processes such as undercutting by river channels or roads."

Comment 4:

Completeness of data:

• A second significant issue arises from the mapping methodology, and the completeness of the data generated. The numbers of landslides mapped in each year are very low compared to other published datasets for this region and period, particularly for the Gorkha earthquake. Whilst I appreciate the difficulties in comparing datasets from different mapping techniques and imagery, the closest comparable effort appears to be that reported in Regmi et al (2016) which is also based primarily on LandSat data. For a smaller area, they map ca. 4,000 landslides, as compared to only 1,400 landslides in a far bigger area in this paper.

Response 4:

We did not map any coseismic mass-wasting, only rainfall-triggered (and previously road-tips, though as mentioned we have now decided not to include these). This is now made clear in the manuscript, lines 103 – 105:

"The inventory does not include any coseismic mass-wasting, as inventories of coseismic mass-wasting triggered by the 2015 Gorkha earthquake already exist [28,32,35]."

As such, the comparison of our data to Regmi et al., (2016) is meaningless, as their inventory was of coseismic failures (and used GF-imagery with a resolution of 0.8 m as well as Landsat, meaning they could map at a far higher resolution anyway).

Where coseismic mass-wasting is considered (i.e. from 2015), we exclusively use the Roback inventory.

Comment 5:

• This apparent difference extends to a comparison of the aerial extent of landslides (this paper identifies ca. 17.6 km2 of mass wasting, Roback identifies ca. 87 km2 for 2015 in > 20k events). These differences are surprising given the stated minimum mapped landslide size is small and more comparable to other efforts (300 m2 – although this in itself seems very small, given Landsat pixel sizes….).

Response 5:

As mentioned above, we did not map any coseismic mass-wasting. So the comparison made here is not valid as it is between rainfall-triggered landslides during the 2015 monsoon season and Gorkha coseismic landslides (with the latter coseismic mapped to a much higher resolution).

300 m^2 is not correct, a mistake on our part. It is technically 900 m^2 (i.e. 30 * 30 m), but we round this up to 1000 m^2 in the manuscript. (see line 97).

Comment 6:

• Related, I also note the discussion of the power-law fitting, and the lowest size thresholds identified at around 11,000 m2. One implication of this threshold is that below this size, the inventory is incomplete. Therefore, the completeness of the dataset needs to be fully evaluated – as at the moment the manuscript is very unclear on this and there are stark differences to other research. As this completeness is absolutely fundamental to your calculations of relative impacts on mass wasting from different controls, this needs addressing.

Response 6:

We agree that discussion on inventory completeness is important, and should be given more attention. As such, on lines 590 – 597 we compare the power-law / area-frequency results of our mapping to others:

"Note that our x-min values are an order of magnitude larger than the x-min values obtained for the Gorkha coseismic landslide data base of Roback et al. [28] and the monsoon-triggered landslide data of Marc et al. [4]. This is likely a result of the difference in mapping resolution between this study

and the others, with the minimum possible size feature that could be mapped by Roback et al. [28] *and Marc et al.* [4] *an order of magnitude smaller than could be mapped here. However, our cut-off x-min values are comparable to similar studies using imagery with 30 – 15 m resolution imagery* [77]*, suggesting that our inventory is as substantially complete as would be expected given the resolution of the satellite imagery."*

As we could only map events > 1,000 m^2, when comparing relative impacts of ASM and 2015 coseismic, we now only compare across a comparable size range (i.e. we remove all Roback events below 1,000 m^2 so we are comparing like for like in terms of event sizes). This information is now included in lines 652 – 661:

"The comparisons between the ASM-triggered, extreme rainfall-triggered and earthquake preconditioning induced controls are straightforward, as all are based on the same resolution data mapped across the same study region. However, the comprehensive inventory of Gorkha coseismic failures presented by Roback et al., 2018 [28] *was mapped at a higher resolution and not fully coincident with our original mapping extent, though only 171 (0.69%) of the 24,915 events mapped by Roback et al. fall outside of our study region. As such, to comparably estimate the total Gorkha mass-wasting relative to our ASM totals, we remove all landslides from the Roback inventory which fall outside of our mapped region, or have mapped areas outside of the area-frequency range of our inventory (i.e., any coseismic failures with areas < 1,000* m^2*)."*

Comment 7:

• As in previous papers, there is a focus here only on new landslides, rather than any form of reactivations. The justification for this is a little vague, but has implications for the results that are generated: "We also removed landslide reactivations from this second "corrected" volume of mass-wasting. This is because we define earthquake preconditioning, a key process for investigation in this study, as a post-earthquake increase in new ASM-triggered mass-wasting, which would not include reactivations of coseismic or past ASM-triggered deposits." Despite this, it remains true that an EQ might trigger a very small part of a much bigger eventual landslide that fails under rainfall, but this would not be included in this analysis, despite the fact that this event would not have occurred without the earthquake. Without fully evaluating the relative contribution of reactivations versus new landslides, I don't think you can just exclude these, particularly given the ambition here for time-dependent susceptibility assessments. From a hazard perspective, this is also problematic – a farmer in Nepal doesn't care if he is hit by a new landslide or a reactivation – it is still happening because of the earthquake and subsequent rainfall… This choice again adds a small but critical subtlety to the simple summary figures that are the headline of this paper, which I feel are problematic without proper

explanation.

Response 7:

We agree that the inclusion (or not) of reactivations is debateable, particularly when it comes to discussions about preconditioning. Indeed, the reason for having the corrected and uncorrected rates was to see the changing impacts of reactivations through time. By excluding reactivations from the uncorrected rates, we did not intend to exclude them fully from the discussion, only to highlight that much of the extra rainfall-triggered mass-wasting post-2015 is due to reactivations rather than new failures, which is an interesting observation in its own right. To make all of this clearer, and to ensure the subtlety of the discussion is preserved, we are now much more careful of our definition of preconditioning, offering two potential definitions on lines 303 – 312:

"However, whether it suggests the landscape had recovered by 2016 – 2017 depends upon the precise definition of earthquake preconditioning. If preconditioning is defined to include any exhumation and transport of material, i.e. to include both an increase in newly triggered landslides and reactivations of coseismic material, then these results indicate that 2016 and 2017 were still perturbed and that the 2015 Gorkha earthquake had a comparable timescale to the ChiChi event, which showed recovery over 2 – 5 years [20]. However, if preconditioning is more strictly defined as only enhanced mass-wasting due to ground damage, i.e. only newly triggered failures, excluding reactivations of already disturbed coseismic material, then our results suggest that the landscape had actually recovered by 2016, with corrected rates in 2016 within +/- 1 SD of the normal."

We hope that adding this nuance goes some way to clarifying why we separated reactivations and new failures, and hopefully provides much more useful insight into the potential processes and timescales of earthquake preconditioning.

Comment 8:

Non-stationary baseline conditions:

• An overarching concern is the very simplified view of the conditions that are potential controls on mass wasting in this part of the Himalayas during the study period. Critically, the baseline conditions are far from stationary. The period considered has experienced considerable social, political and economic change which remains highly challenging to disentangle from impacts on mass wasting. The period covers significant outmigration of working age males – with a well-documented impact on the degradation of agricultural land, the civil conflict – which dramatically impacted farming practise, the changes in the planning process – which influences when and where developments such as road construction takes place, significant urbanisation, plus much much more… The processes upon which you focus (roads, ASM, EQs, extreme rainfall) are playing out upon this backdrop, and I would argue cannot be separated from this without a considerably higher resolution and precision of

data. I don't therefore believe that it is possible to 'isolate' the influence of the chosen factors as is suggested here. In the simplest sense this issue is illustrated in the calculation of the long-term average mass wasting rate – which by definition assumes zero change in background conditions by over the 30 years – when these long-term changes may each be a significant and interrelated catalyst in promoting mass wasting.

Response 8:

We fully agree that the socio-economic processes described here will have some impact on mass-wasting (another reason for including the 20% mapping error/uncertainty), and would be a fascinating area of future research. However, accounting for this is currently beyond the scope of this paper, so we have to assume that at a given time, all natural mass-wasting drivers will be impacted similarly by socio-economics. We appreciate that this is a big assumption, but as we say, disentangling the socio-economics from the natural processes is far beyond what can be achieved here. Furthermore, it is the road-tipping that is most likely to have been impacted by changing socio-economics, which is another reason why we have decided to no longer consider roads within this analysis.

Comment 9:

• Related to this, the word 'correlation' is in my view used incorrectly throughout the manuscript – without direct data on the controls rather than just mass wasting on its own, this analysis does not really go beyond identifying 'coincidence'.

Response 9:

In retrospect, we agree that in some places our use of correlation was inappropriate, and have changed terminology to coincident where necessary (e.g. lines 173-175 now read: *"Instead, both perturbations were coincident with "cloud-outburst" extreme rainfall events that are reported in the literature").*

We do however continue to use correlation for figure 2, the fit of which we do consider to be correlated.

Comment(s) 10:

Assessment of roads:

• The primary impact of roads on mass wasting in the Himalaya is from rural or seasonal roads constructed with minimal engineering input. Rural roads (ca. 2 – 3 m in width, unsurfaced and commonly with minimal spectral contrast to the surrounding landscape) are arguably invisible – or at best inconsistently visible – in Landsat imagery (15 m, 30 m). Evidence to the contrary needs to be provided if this is how this has been done… I am therefore unclear on the confidence with which the association between roads and landslides can really be made when the roads must be barely visible in the imagery used here. The method to identify this association is also not well explained so this analysis remains unconvincing.

• The only fact presented about roads is the timing of a World Bank funded program – this seems a far too simplistic association to imply in the absence of any data on roads, particularly given the far wider range of donor funded road and rural access programs that have rolled out during this study period, in addition to the wider set of influences on road construction in Nepal (see above, and below).

• A key further consideration is that rural road construction in Nepal is widely acknowledged to be a highly seasonal activity, very unevenly distributed through the year. Where the analysis conducted here only samples part of the year (and inconsistently so…), the risk of not capturing the impact of road construction in a uniform manner is therefore high. There are several important related issues for the analysis undertaken: (1) the timing of road construction is closely linked to both the fiscal year and the annual planning cycle in Nepal – with a common rush in road construction towards the end of the former. This research needs to consider how completely road construction impacts are captured if a net impact is to be derived – but I am not clear that the present data can actually do this; (2) The planning process for road construction has changed significantly during the study period considered here – from the 14 step planning process (up until 2017) and the 7 step process thereafter – this significantly influences when roads are built, so there is potential for a shift in bias through the time period considered; (3) related, the budgeting structure and decision making for rural road construction has also changed significantly during this period, with a notable localisation of construction capacity and decision making. A consequence is that when the mapping technique covers the monsoon period only, road construction – and the associated landslides – will potentially not be mapped, which may well be a significant number, and given then the annual date of imagery used in the mapping varies year on year, therefore the period being sampled varies. If road construction was uniformly distributed through the year this might be considered negligible, but it is not, and so this is potentially very important. This variability in road building activity needs to be considered and a demonstration of its role made… Without actual data on road construction, I don't see how this can be achieved, and so this represents a considerable extra effort.

• There is also an implicit assumption that road construction has an instantaneous impact on mass wasting and no longer-term legacy effect, which is surprising given the argument for time-dependent landslide susceptibility. So, I don't agree with this statement: "However, as road-associated mass-wasting is not related to rainfall, a second "corrected" volume of mass-wasting was calculated with this mass-wasting type removed". Road construction can prime a slope to fail if it does not immediately trigger collapse (in the same manner as the EQ preconditioning ideas) – but rainfall remains a key recurrent trigger. In this sense, without more detailed data, it remains very difficult to separate rainfall and road impacts. This really needs thinking through in more detail and supporting with analysis…

Response(s) 10:

Despite the fact that our mapping was continuous (i.e. we will have captured all road-tips above the minimum mappable size threshold), in retrospect we agree with many of the reviewer comments here, and conclude that at the current time we do not have sufficient supporting data to analyse and assess

roads in a manner comparable to the other mass-wasting drivers considered. As such, we have made the decision to no longer consider the explicit impacts of roads, and instead streamline the manuscript to focus on rainfall and earthquakes, with particular attention switched to earthquake preconditioning. The potential impacts of roads on our other mass-wasting drivers is now accounted for in the applied mapping error/uncertainty, which is applied to account for any mapped mass-wasting that is actually primarily triggered by road/channel undercutting rather than rainfall alone.

Comment(s) 11:

Consideration of earthquakes:

• The selection of potentially landslide triggering earthquakes has a weak justification. The 30 km radius for inclusion is arbitrary, and more fundamentally using epicentre is a very crude approach to identifying which events might be important when we know that the spatial distribution of PGA or PGV is what controls landsliding. It is impossible to associate the pattern of PGA or PGV to the epicentre location alone. If the analysis were to consider for example the extension of the 1g isoseismals into the AOI for all earthquakes during the mapping period, I would not be surprised to see the 1988 and 2011 earthquakes included. This really needs to be considered, and if no impact on mass wasting is observed for these events, then why is this the case?

• The paper considers to some extent the ideas around the legacy effects of previous earthquake damage in conditioning subsequent mass wasting, although doesn't really get into much detail on this idea. Research on this topic is in its infancy, but some key issues seem to be overlooked here. The legacy damage effect of earthquakes is not just experienced in the transient response immediately (1 – 5 years) after the initial shaking. For example, the Parker et al paper referenced identifies damage legacy effects manifest over intervals of ca. 40 years for Mw7.7 and & 7.1 events in NZ. With this in mind, it is surprising here not to see any consideration of the potential legacy of historic and very much larger earthquakes which have directly impacted the AOI (in addition to 1988, 2011, also 1934, 1833, 1505 and 1255…).

Response(s) 11:

Our revised manuscript includes a substantially expanded section on earthquake preconditioning. As suggested by the reviewer, we now dedicate several paragraphs to the analysis of the potential longer term impacts of older earthquakes in 1934, 1988 and 2011 that were coincident with our study region. This is included in lines 314 – 352:

"This analysis provides insight into short-term Himalaya preconditioning of the type observed by Marc et al., [20]*. However, as already described, preconditioning has also been observed over decadal timescales* [33]*, suggesting that there may be two distinct modes of preconditioning. One, short-term preconditioning via near-surface damage that is rapidly exploited by subsequent rainfall as new failures. And two, decadal scale preconditioning that is seemingly only exploited during subsequent*

large-magnitude earthquakes [33]. This longer-term preconditioning is less well understood than the short-term, but could be due to deeper bedrock damage that cannot be exploited by near surface processes (e.g. rainfall), and thus only becomes apparent when that bedrock is exhumed to the surface or impacted by a subsequent large magnitude earthquake. As our study region is coincident with the rupture zones of several M_w 6.0 earthquakes over the past century (1934 M_w 8.0, 1988 M_w 6.9, 2011 M_w 6.9; Fig. 6), this study is well placed to investigate whether these earlier earthquakes had any impact on landsliding following the 2015 earthquake, and thus provide much needed insight into longer-term modes of preconditioning. Using the same grid cells as shown in fig. 5, we calculate for each cell with less than 20% ice cover the summed maximum observed USGS calculated Peak Ground Accelerations (PGA) [49] from the two largest 2015 earthquakes (the 25th April mainshock and 12th May aftershock), and from these 2015 events plus preceding earthquakes in 2011, 1988 and 1934. We then plot these summed PGAs against each grid cell's 2015 monsoon season percentage change in mass-wasting volume density (m^3/km^2) (Fig. 7a – b). Surprisingly, there is no relationship between cumulative PGA and mass-wasting increase in either case (R^2 = 0.097 and 0.062), with the addition of historic earthquakes weakening the fit, suggesting that these older earthquakes have not induced any decadal scale preconditioning on the landscape. This raises the question of whether it is PGA alone that induces earthquake preconditioning. As seismic ground motion is known to undergo amplification when travelling across ridgelines [50–52], earthquake preconditioning may be most likely where high PGA is coincident with high excess topography, where excess topography is defined as the volume of rock-mass above a landscape's threshold angle [53]. To investigate this, we first plot the average excess topographies of each grid cell used in Figs 7a-b (see methods) against the respective percentage changes in mass-wasting across those grid cells (Supplementary materials, Fig 1). We find that the relationship between excess topography and 2015 monsoon-triggered mass-wasting is stronger than that of cumulative PGA, with an R^2 value of 0.34. We then weight the summed PGA's used in Figs 7a -b by the average excess topography of each grid cell, and re-plot these weighted values against 2015 percentage mass-wasting change (Fig. 7c - d). PGA weighted by excess topography has a stronger relationship with 2015 mass-wasting than either factor alone, with R^2 values of 0.46 in the case including only the 2015 PGA, and 0.56 in the case including historic earthquake PGA. This suggests that it is PGA coincident with excess topography that leads to enhanced preconditioning, and that large earthquakes in 1934, 1988 and 2011 were still exerting a legacy effect on the landscape in 2015."

We also dedicate new analysis and several paragraphs to the discussion of why the 2015 earthquake induced a large short term perturbation, but 1988 did not. This is included in lines 354 – 389:

"Whilst the earthquakes in 2011 and 1988 may have imparted a longer-term impact on the landscape, our normalisation suggests that they did not induce any short-term perturbations such as that observed following the 2015 earthquake. Unfortunately, we were not able to map the mass-wasting

during 2011 and 2012 due to scan-line errors in the Landsat 7 satellites. However, we can undertake a direct comparison between 2015 and 1988 to investigate why 2015 caused significant short-term perturbations and 1988 did not. One potential explanation is that the PGA induced in 1988 was lower than those induced in 2015. Indeed, according to the USGS, the maximum PGAs induced within our mapping region by the 1988 event was 0.28 g, compared to a maximum of >0.74 g in 2015 (Fig. 6). To further investigate these differences, we again divided our study region into the same grids used to analyse the extreme rainfall (Fig. 5), and counted how many grid cells exceeded PGA thresholds of 0.1, 0.3 and 0.5 g in 2015 and 1988. Of those cells experiencing > 0.1 g, we then counted how many experienced a percentage increase in mass-wasting volume density (m^3/km^2) in the 2015, 1988 and 1989 post-earthquake monsoon seasons, as well as the average mass-wasting percentage change across those cells (Table 2a). We included the 1989 monsoon seasons as it was the first full monsoon-season after the 1988 earthquake, which occurred on 21/08/1988. This analysis highlights that the 2015 main rupture zone was more coincident with our mapping region than 1988, with 54 cells impacted by > 0.1g in 2015 compared to 38 in 1988, had higher and more widespread PGA values > 0.3 g, and had a higher average percentage change in mass-wasting across the affected grid cells, with a 164% average increase in 2015 compared to 71% in 1989.

Furthermore, our previous analysis suggests that excess topography also plays a potentially important role in controlling earthquake preconditioning. To investigate whether this was the case for the 2015 and 1988 earthquakes, we directly compare the impacts of 2015 and 1988 across a comparable range of PGA (0.1 – 0.3g; Table 2b). This highlights that even across the same PGA range, the 2015 monsoon experienced much higher average mass-wasting than 1989, with an average increase of 328% compared to 168%. This is despite the fact that the 1989 monsoon season observed far higher rainfall, with our normalisation (Fig. 3) highlighting that the increases observed in 1989 are only slightly higher than expected given the high monsoon strength. In other words, our normalisation suggests that the above-average 1989 mass-wasting is attributable to the higher than average monsoonal rainfall. This suggests that the 1988 earthquake did not induce any comparable preconditioning to 2015, even across the same PGA range. This can again be explained by excess topography, which was three times higher in the grid cells impacted by the 2015 earthquake. This provides further support to the idea that the topographic signature of earthquakes leads to preconditioning being induced only where high PGA is coincident with high excess topography, which did not occur in 1988."

Overall, we believe that these new results are very novel, particularly for the Himalayas, and provide significant further insight into the processes and timescales of earthquake preconditioning.

Comment(s) 12:

Consideration of extreme rainfall:

• I'm very surprised given the 30-year period, the extent of the study, and the local climate, that only two extreme rainfall events have been identified as having an influence on mass wasting. This potentially shows the relatively insensitivity of the approach described. I note that the perturbations are identified and then a driver is sought, rather than looking at drivers and trying to identify if a mass wasting response occurred…this seems a slightly circular approach. Given that there is no data on extreme rainfall considered here to assess which events have consequences, and which don't, by only considering two events this analysis does not seem comprehensive enough to really evaluate the net impacts of extreme rainfall events.

Response(s) 12:

We thank the reviewer for these points. As such, we include significant new analysis and discussion where we define and identify all extreme storm events, and then look at mass-wasting response, as suggested. We do this using pre-existing rainfall indices (derived from the TRMM4B42 product). This new analysis is included on lines 181 – 270:

"These reports suggest that the 1993 and 2002 perturbations were due to extreme rainfall events that caused significantly more mass-wasting than that caused by a typical ASM season. This raises several important questions. First, are these the only such extreme rainfall events to have occurred across the mapped period? Or, have other similar events occurred without triggering significant mass-wasting? Second, what is the return period of these events? This latter point is particularly important, as quantifying the expected frequencies of extreme rainfall events is vital for hazard management, as well as for gaining a better understanding of longer-term mass-wasting contributions.

To understand the frequency and impact of extreme rainfall events, we obtained and analysed a dataset of extreme precipitation indices derived by Stanley et al., [42] that are recommended for use in relation to precipitation extremes by the Expert Team on Climate Change Detection Indices (ETCCDI) [43]. For this analysis we use the RX1 index, which is the highest annual 24-hour precipitation total. These data are derived from the TRMM3B42 climate record, a product which has already been found to show co-occurrence between extreme rainfall and landsliding in the Himalayas [42], has a spatial resolution of 0.25 x 0.25^o and covers the period 1999 – 2017. Whilst these data do not cover our entire mapped period, they still allow us to compare 17-years of mass-wasting to extreme precipitation, including the 2002 perturbation.

Before quantifying the frequency and impact of extreme rainfall events, it is necessary to precisely define what is meant by "extreme". In this case, we divide our study region into 85 grid cells and define "extreme" based on the grid cell RX1 values observed in 2002 (Fig. 5). The 11 grid-cells impacted by the 2002 event observed an average RX1 value of 208 mm. However, as the Himalayas have a strong precipitation gradient [30,44], it would not be robust to define extreme rainfall only in terms of an absolute value. This is because cells in the higher Himalayas, which generally experience less rainfall but have

a more mass-wasting prone topography, could have lower absolute rainfall thresholds for triggering significant mass-wasting than the south, where the 2002 event occurred. As such, we also define extreme rainfall according to a grid cell's RX1 percentage change from its mean value. In 2002, the average RX1 percentage increase across the affected cells was 79%. We therefore define a grid cell as having experienced an extreme rainfall event if the RX1 value is > 200 mm, and/or >80% higher than its average.

Using this definition, we extracted every grid cell per year that exceeded the extreme thresholds. We also calculated the total mass-wasting volume density (m^3/km^2) for each of the extracted grid cells, as well as the associated percentage change in mass-wasting volume density from each of those cell's means. As summarised in Table 1, between 1999 and 2017, 48 cells met the definition of extreme, which under the assumption that contiguous cells from the same year are from the same event, equates to 13 individual extreme rainfall events. Of these rainfall events, seven show a clear mass-wasting response, with the highest percentage changes in volume density (60 - 2400%) observed in 2002 (Table 1). Of the six extreme rainfall events that did not cause a mass-wasting response, five occurred across cells that are almost permanently covered by ice in the High Himalayas (Fig. 5), and thus rarely experienced mass-wasting in any years. The final cell not to show a response is partially coincident with the flat Terai plains, the topography of which would not promote significant mass-wasting even under extreme rainfall conditions.

With seven mass-wasting triggering extreme rainfall events now identified, the total mass-wasting volume caused by these events can be estimated. From the equations in Fig. 2, we estimate how much total mass-wasting would have been expected in each year based on the total monsoonal rainfall. Consequently, on the assumption that any mass-wasting above these estimates in the years known to have extreme rainfall events are due to those events, the total extreme rainfall generated mass-wasting can be estimated from the sum of the differences between the actual and predicted total volumes for those years (1993, 2000 – 2004, 2006). This gives a total extreme rainfall-triggered mass-wasting volume across the entire mapped region of 8.93×10^7 m^3, which, given the average mass-volume caused by a non-perturbed monsoon season across the same region is 1.87×10^7 m^3 (see methods), equates to approximately 4.7 ASM-seasons of mass-wasting.

This total is likely to be an under-estimate of extreme rainfall-triggered mass-wasting, as smaller extreme rainfall events outside of the period covered by the RX1 data may have been missed. Furthermore, owing to the coarse resolution of the TRMM data, any highly localised extreme rainfall events with a smaller spatial extent are not resolvable. However, any underestimation caused by unidentified smaller extreme rainfall events is unlikely to be significant. Of the total mass-wasting volume estimated for the known extreme rainfall events, 43% was from the 1993 storm, 54% was from the 2002 storm, and just 3% due to the smaller storms identified in other years. Thus, even if smaller-

scale extreme rainfall events have been missed, their mass-wasting contributions will have been insignificant relative to the large-scale events in 1993 and 2002.

The explanation for why the 2002 event was more erosive than the others is likely due to the fact that its spatial extent was significantly larger than any other event, and its absolute and percentage changes in RX1 values (300 mm; 160%) were the largest of all the identified extreme rainfall events. The 2002 event affected at least 13 cells, whereas only one of the other identified mass-wasting triggering extreme rainfall events affected more than five cells, which was nine cells in 2004. Furthermore, the two largest area extreme rainfall events since 2002 (in 2003 and 2004) were partially coincident with the extent of the 2002 event, with none of the coincident cells in 2003 and 2004 experiencing the expected mass-wasting increase. This suggests that the landscape required several years to recover and re-accumulate sediment following the 2002 exhumation event, an observation that is comparable to the 10^0 - 10^2 post-extreme event recovery times estimated in other mountain ranges [45].

Having quantified the mass-wasting impact of extreme rainfall events, we also estimate their return periods. In 17 years, this region experienced seven extreme events, giving a recurrence interval of 2.6 years. However, 2002 was the only storm of its type, both in terms of spatial extent and mass-wasting response, to occur across the 17 years, giving a recurrence interval for this scale event of 18 years. However, if as suggested by the literature the 1993 event is comparable to 2002, and that as suggested by our mass-wasting time series no other events of this scale occurred across the mapped 29 years, a recurrence interval of 15 years for this scale event may be more appropriate."

Overall, this approach has allowed us to much more comprehensively assess the net impacts of extreme rainfall events, at least for 17 years of our time series, which is still a far longer record than any other studies we have seen.

Comment 13:

• I am uncomfortable about relying on 'using the methods described by Marc et al. 21', - this paper is not a good example of clarity of explanation and I would strongly suggest the methods used here are fully explained to avoid perpetuating ambiguity.

Response 13:

We only used the "methods described by" terminology in the manuscript itself, and do include the full outline of Marcs methods in the methods section on lines 600 – 621:

"The empirical relationships between ASM precipitation and mass-wasting can be used to predict how much background mass-wasting would have been expected to occur each year based on that year's rainfall. The equations derived for the different measures of mass-wasting volume (Fig. 2) are, where V is volume in m^3/km^2 and x is grid averaged precipitation in mm:

- $V = 36.855e^{0.00288x}$ *for uncorrected total volumes.*
- $V = 28.083e^{0.00298x}$ *for corrected total volumes*
- $V = 1.9245e^{0.00287x}$ *for uncorrected scar volumes*
- $V = 1.5053e^{0.00290x}$ *for corrected scar volumes*

For each measure, the ASM-strength normalised rate for each year is then calculated by taking the ratio of the actual mass-wasting mapped for that year to that predicted by the above equations. I.e. a value of one indicates that the actual observed mass-wasting in a year is what would be expected given the ASM-strength, whilst a value significantly above one indicates that there was more mass-wasting than expected given the ASM-strength, implying perturbation above the background by some other event. The errors in the normalised rate include the standard error in the data points used to calculate the prediction equations, an assumed standard deviation of 20% in mass wasting area to account for variability in mapping period, and the standard deviations reported in the area-volume conversion parameters. Assuming that these errors are uncorrelated, they were combined using standard Gaussian propagation of error to obtain the uncertainties for each measure as displayed in Fig. 3. Furthermore, as in the analysis of Marc et al., [4], the 2014 Jure landslide was removed from the analysis, as this event is widely considered to have been caused by progressive failure across multiple years, and so is known not to be attributable to any single ASM-season [4].”

Comment 14:

• The choice of rainfall product really needs to be justified and evaluated (e.g. see Ullah et al., 2018, Remote Sensing). For example, as the rainfall data has ca. 28 km grid size, how does this compare to storm size and how does it influence total monsoon rainfall calculations in an AOI with a significant precipitation gradient, and how does the averaging in the derivation of this product, and in your subsequent treatment of the data behave over mountain topography?

Response 14:

A full treatment of precipitation gradients over mountainous topography is beyond the scope of the research undertaken here. However, to address this issue we include a new paragraph dedicated to evaluating the literature on the PERSIANN-CDR product on lines 543 – 562:

“The PERSIANN-CDR product is now a widely used product, which has been well evaluated in the literature (e.g. Nguyen et al., [69] and references therein). Indeed, PERSIANN-CDR was found to perform excellently when evaluated against 1400 ground-stations at capturing the spatial and temporal patterns of rainfall in the monsoon-regions of eastern China [70], and outperformed the TMPA dataset in its ability to capture the overall characteristics of Hurricane Catrina [69]. Furthermore, the PERSIANN-CDR product was found to have lower monthly mean variance when compared to other satellite derived products, showing particularly small variance with the GPCP1DD product [71,72]. Similarly, despite

being slightly outperformed by other products, the PERSIANN-CDR data set was capable of capturing inter-annual monsoon precipitation in Pakistan, with high (0.8) R values when compared to in-situ data [73]. However, it should be noted that the PERSIANN-CDR product has some limitations. First, it is reported to have a tendency to under-predict values of extreme precipitation [70]. It for this reason that we used the TRMM3B42 derived extreme precipitation indices to further investigate extreme rainfall events. Second, as with all satellite derived products, it remains unclear how well orographic effects are captured [74], which is an ongoing research area within meteorology. However, a benefit of the PERSIANN-CDR product is that it is designed specifically for use in longer-term studies[37] and is considered one of the most temporally homogenous products. As such, unlike other satellite products whose methodologies could introduce temporal variance, any errors in the PERSIANN-CDR product introduced by orographic effects should be more systematic through time, and so not significantly bias our time-series."

On the topic of grid-size, it is undoubtable that some very localised storms will be missed by rainfall products of this scale (TRMM is also 28 m). However, as we show in our analysis, the mass-wasting impacts of small localised storms (even those of size > 28 km) are negligible compared to the large spatial extent events such as 2002, as outlined on lines 242 – 249:

"Furthermore, owing to the coarse resolution of the TRMM data, any highly localised extreme rainfall events with a smaller spatial extent are not resolvable. However, any underestimation caused by unidentified smaller extreme rainfall events is unlikely to be significant. Of the total mass-wasting volume estimated for the known extreme rainfall events, 43% was from the 1993 storm, 54% was from the 2002 storm, and just 3% due to the smaller storms identified in other years. Thus, even if smaller-scale extreme rainfall events have been missed, their mass-wasting contributions will have been insignificant relative to the large-scale events in 1993 and 2002."

Comment 15:

- The paper makes claims around showing a link between ASM and mass wasting, and hence hazard. Given this, it is not clear why no attempt to compare the outputs to more widely used monsoon strength indices is made (e.g. SASMI). I note the Petley et al (2007) paper reference conducts a comparable analysis for fatal landslides in Nepal with SASMI, and identifies what on the face of it is the inverse to the relationship you show, but does go on to offer some explanations of this. This is important as 'monsoon strength' means different things to different people – and most will read this as measures such as SASMI, rather than a bespoke Nepal specific strength measure.

Response 15:

We did look at the relationship between our rainfall triggered mass-wasting and SASMI, but found no relationship between the two. We suspect this is because SASMI is a very regional index, which simply doesn't capture the localised changes in rainfall observed across our much smaller overall study region. As mentioned, Petley found an inverse relationship, and whilst he offers some explanation for this, it still highlights the fact that very large extent indices such as this are actually not immediately appropriate for use in smaller sub-regions. However, we do appreciate that our use of the term "strength" could induce confusion. As such, we explicitly define what we mean by ASM-strength, and make it clear that in this case the term is used to mean total May – September rainfall in our study region. This is now included in lines 122 – 127:

"We use total May – September precipitation as a proxy for local monsoon strength, as typical measures of monsoon strength such as the SASMI [39] are derived over extensive regional scales, and do not accurately capture local changes in monsoon conditions across the study region. As such, from this point forward, the term "ASM strength" is defined here specifically as total May – September precipitation across the mapped region."

Comment 16:

• The LandSat derived background mass wasting rate is compared to the data from Roback to assess the relative impact of the Gorkha earthquake. As far as I can understand from the explanation, this appears problematic because (1) the Roback data is much higher resolution, is collected over an area that is entirely impacted by the earthquake, and is data collected from imagery over < 60 days (these factors all derive a very high event mass wasting rate), but (2) the data that this is compared to presented here is collected over a far bigger area where only a small portion is directly impacted by the Gorkha earthquake, the resolution and completeness is at least an order of magnitude lower, and the time period of mapping is much wider. All of these factors will accentuate the difference between the short and long term mass wasting rates. This just does not appear a valid comparison to make. It is not clear why the data mapped here for 2015 is not used here, or does the relatively limited number of mapped features inhibit this…?

Response 16:

The aim of the relative comparison between the overall 2015 coseismic mass-wasting the mass-wasting typically triggered in an average (unperturbed by extreme storms) ASM seasons. So the fact that Gorkha was mapped over 60 days is irrelevant. The coseismic events in the Roback inventory are predominantly assigned to either the April main shock or the May aftershock, i.e. represent relatively instantaneous mass-wasting that was then mapped over a 60 day period. We simply want to estimate the total coseismic mass-wasting triggered by Gorkha relative to what is triggered across an average 5 month ASM-seasons, so the issues of mapping timescale difference is very much built into the comparison / not relevant.

However, we accept the issues about differences in mapping resolution, and the fact that the Gorkha coseismic inventory includes events at a scale we could not map. As such, as already discussed above, in the revision we only make this comparison for coseismic events above the mappable size range. This should make the comparison more applicable.

Furthermore, we accept that the region impacted by Gorkha is slightly smaller than our overall study region, though still impacted ~50% of it. However, the aim of this comparison is not to just look at the region impacted by an earthquake and see how this compares to the monsoon, but rather to look at a given large region of the Himalayas, and for that region see how much mass-wasting across longer timescales is due to earthquakes vs rainfall. If you only ever looked at the specific regions bounded by high earthquake PGA, you will ignore vast regions that are continuously impacted by rainfall, but never by large magnitude earthquakes, thus underestimating overall impacts of rainfall through time, and overestimating earthquakes. By taking a large region, that is roughly 50% impacted by a single earthquake, we can thus get an appreciation of the relative impacts of earthquakes vs rainfall, which would include the fact that overall earthquake extents are far less than rainfall extents – i.e. the argument about low-frequency, localised high-magnitude events vs high-frequency, large-extent, low magnitude events.

And in response to the final comment, again, we did not map coseismic failures ourselves, and as the Roback inventory is the most complete coseismic dataset for Gorkha this was used.

Comment 17:

• This point above also raises a question about how to compare rates between the controls identified. For the extreme rainfall, a local average rate is derived and compared to one specific to that event. This seems different for how the comparison is undertaken for Gorkha, where rates for the whole area impacted are considered. As it is known (and shown in Figure 1) that landsliding varies considerably across this area over the long term, how then is the impact of roads handled – is this a deviation in local mass wasting rate for the location of the road only, or a net change in rate for the whole area? When comparing events and controls, how each is treated needs to be consistent, which does not appear to be the case at the moment…Again this has a big influence on the simple summary stats….

Response 17:

We accept that we needed to be clearer with how we compare drivers over different scales. We no longer look at roads. As for the other drivers, we use two consistent scales for comparisons in the revision. One, we look at specific extreme storms / localised earthquake preconditioning using the grid-cells in figure 5. Two, when making the overall comparisons between net contributions of each driver, we use the scale of the entire region, estimating the total net contribution of each across our whole mapped area, as outlined on lines 624 – 662:

“With multiple different mass-wasting drivers isolated, we can estimate the relative contributions of each to overall mass-wasting across our mapped region. Across this region, the average ASM-triggered mass-wasting volume for unperturbed years (1988 – 1992, 1994 – 1999, 2005, 2007 – 2014) is 1.89 x10^{7} m^{3}. From the equations in fig. 2, we know how much mass-wasting would have been expected based on the ASM-precipitation in each year. As such, on the assumption that any mass-wasting above the expected in years known to observe an extreme event are due to that event, the total mass-wasting attributable to each extreme event type can be estimated from the difference between the expected and actual.

To estimate the total relative mass-wasting due to extreme rainfall events, we first calculate the sum of the differences between expected and actual total mass-wasting volumes for all years known to have experienced a storm (1993, 2000 – 2004 and 2006). This gives a volume of 8.93x10^{7} m^{3,} which is equivalent to ~4.7 average ASM seasons.

The estimate of total relative mass-wasting due to earthquake-preconditioning depends on the precise definition of what earthquake preconditioning includes. By the strictest definition of preconditioning, which as discussed above would only include fresh failures, the years 2015 – 2018 observed a total corrected volume of 7.9 x10^{7} m^{3} yet were predicted to have experienced just 3.9 x10^{7} m^{3} based on the rainfall totals and the equations in fig. 2. This gives a difference of 4.1 x10^{7} m^{3}, which, given the average mass-volume caused by a non-perturbed monsoon season across the mapped region is 1.89 x10^{7} m^{3}, equates to approximately 2.2 ASM-seasons worth of mass-wasting. However, by a looser definition of mass-wasting, which includes reactivations of coseismic mass-wasting, the years 2015 – 2018 observed a total uncorrected volume of 1.4 x10^{8} m^{3} despite only being predicted to have experienced 4.7 x10^{7} m^{3}. This gives a difference of 9.6 x10^{7} m^{3}, which equates to approximately 5.1 ASM-seasons worth of mass-wasting.

The estimate of total relative mass-wasting due to the 2015 Gorkha coseismic failures is more uncertain. The comparisons between the ASM-triggered, extreme rainfall-triggered and earthquake preconditioning induced controls are straightforward, as all are based on the same resolution data mapped across the same study region. However, the comprehensive inventory of Gorkha coseismic failures presented by Roback et al., 2018 [28] was mapped at a higher resolution and not fully coincident with our original mapping extent, though only 171 (0.69%) of the 24,915 events mapped by Roback et al. fall outside of our study region. As such, to comparably estimate the total Gorkha mass-wasting relative to our ASM totals, we remove all landslides from the Roback inventory which fall outside of our mapped region, or have mapped areas outside of the area-frequency range of our inventory (i.e., any coseismic failures with areas < 1,000 m^{2}). The total volume of the remaining 12,843 coseismic failures is 3.32x10^{8} m^{3}, which equates to 17.6 ASM-seasons worth of mass-wasting across our study region.”

Comment 18:

• Figure: 1 – no admin boundaries make this difficult to orient. DEM data has no source / attribution. DEM scale is odd – seems to be reporting a new highest place on earth! Surely the max elevation is 8,848 m, not >11,000 m?

Response 18:

Added the outlines of the Districts of Nepal to help orient figure. DEM issue has been solved (DEM corrected and filled), with correct elevation now stated. Attribution to JAXA/ALOS now included in figure reference.

Comment 19:

• Figure: 2 – confidence intervals would be useful here, particularly given the variability on the log axis.

Response 19:

We have added error bars to all points to help the reader get an understanding of the uncertainty – this error includes the 20% mapping error applied to all events (intended to account for unseen impacts of undercutting, socio-economics, varying mapping intervals etc.) and the standard deviations in the Larsen parameters used to undertake the area to volume conversions.

Comment 20:

• Figure: 3 – it would seem sensible to only show EQs of sufficient magnitude to trigger landsliding (e.g. 6Mw…) not all.

Response 20:

Fair point, now only show the main earthquakes in 2015, 2011 and 1988 (the only ones above M_w 6.0).

Comment 21:

Supplementary data:

• The mapped shapefiles would be more widely useful than the *.txt file of summary stats.
• I would also like to see the full inventory posted to this repository for wider scrutiny and for use in other research: https://www.sciencebase.gov/catalog/item/586d824ce4b0f5ce109fc9a6

Response 21:

We said this to the editor in the initial cover letter, but may not have reached the reviewers: we fully commit to publishing the full dataset as shapefiles shortly after the publication of the paper.

Reviewer #3 (Remarks to the Author):

Oliver Francis

Comment 22:

The work presented here in this manuscript quantifies the volumes of mass wasting produced by different processes across a 30 year time period and argues these are meaningful for understanding long term erosion rates and forecasting changes due to climate change or road construction. However, without an understanding of the frequency of these processes it is impossible to convert these volumes into rates and put them into context of time scales longer than the 30 years recorded here. I recommend identifying the return period of the extreme rainfall events in 1993 and 2002 and comparing those with the frequency of Gorkha Earthquake in order to fully understand their relative contributions to erosion rates.

Response 22:

We agree that an analysis of event frequency is necessary to facilitate a full understanding of the relative long-term contributions of the events we have identified, and have made this clear in the manuscript on lines 185 - 188:

"Second, what is the return period of these events? This latter point is particularly important, as quantifying the expected frequencies of extreme rainfall events is vital for hazard management, as well as for gaining a better understanding of longer-term mass-wasting contributions."

As outlined to reviewer 2 in comment/response 12, we have then substantially expanded our section on extreme rainfall, where we now specifically we specifically define extreme storms based on the 2002 event (lines 200 - 212), before using a new dataset of extreme rainfall data (lines 190 - 198) to identify how many extreme storm events similar to 2002 have occurred (lines 214 - 226), and thus estimate recurrence intervals (lines 263 – 270). We also compare these to the estimated recurrence intervals of earthquakes of magnitude comparable to Gorkha (lines 418 - 422).

Comment 23:

Along a similar line I also found the quantification of the contribution of road construction to mass wasting confusing. It is unclear in the manuscript how these mass wasting events are identified, are they simply the volume of material produced during the construction? Or do they include the enhanced mass wasting associated with undercutting of slopes and poor slope reinforcement? Separating these two processes is important for understanding the continued contribution of road construction to mass

wasting after the study period. It is also unclear how the recorded areas are converted to volumes; these events are not landslides so using a landslide area – volume scaling is not appropriate. Volumes of material produced by the construction of the roads could be estimated by measuring the width of the roads and the heights of their cuttings into the hillslope. Accurate estimations of the mass wasting produced by construction and post construction along with road mapping would allow forecasting of mass wasting of future construction projects.

Response 23:

We agree that separating road-tips (which is exclusively material excavated by during road construction and dumped onto hillslopes) and mass-wasting enhanced by road undercutting. The events we mapped were exclusively road-tips and did not include any enhanced monsoon-triggered mass-wasting due to road undercutting. This is because identifying which monsoon-triggered mass-wasting events were influenced by roads, and to what degree a given road actually influenced a given mass-wasting, would require far higher resolution road-data than is available for much of our time series. We account for the influence of road undercutting on our monsoon-triggered events by implementing a 20% error on all areas, where part of this error is designed to account for any mass-wasting events mapped as monsoon-triggered, that were actually influenced by undercutting by rivers or roads. We had been using Larsen's shallowest soil landslide type parameters for calculating the volumes of these road tips, but in retrospect we agree this is inappropriate. Furthermore, whilst we agree that in theory it could be possible to estimate the volumes of road tips by measuring road widths and cutting heights, this would require DEMs with a resolution of at least 5 m. Unfortunately, we only have access to 30 m DEMs, and whilst 12.5 m DEMs could be purchased, this would still not be sufficient to accurately estimate road widths/heights, which are typically < 10 m. As such, we are unfortunately unable to estimate road volumes at the current time, and consequently have made the decision to remove all work relating to road-tipping from this manuscript. Whilst we think it presents a fascinating area of further study, we simply do not have sufficient data at the current time to properly assess road impacts, so believe that removing roads and focusing on earthquakes vs rainfall will make the manuscript more streamline and impactful.

Comment 24:

Line 54: I find the definition of the "background" rate confusing. Is the background rate simply the estimated rates of mass wasting from the monsoons? Or does it also include storm induced mass wasting outside of the monsoon months? Clarifying this definition along with the timing of the monsoon will help to better explain the aim of the paper.

Comment 24:

The word "background" was intended to refer only to mass-wasting due to the annual monsoon. I.e. to only include regular low-magnitude drivers, and not include low-frequency high-magnitude drivers such as storms and earthquakes. This is now defined / discussed on lines 39 – 43 and 61 – 64:

"Background rates of mass-wasting are driven by tectonic uplift [2,7] *and climate* [8–11]*, though the relative contributions of these controls over geological timescales is difficult to unravel* [12]*. At shorter timescales, background rates of mass-wasting are transiently perturbed by a variety of low-frequency, high-magnitude drivers such as extreme rainfall, earthquakes and floods* [13–17]*."*

"High rates of tectonic uplift and the Asia Summer Monsoon (ASM) drive high background rates of mass-wasting [18,21–24]*, which are perturbed by extreme events such as floods* [25]*, extreme rainfall* [26,27] *and earthquakes* [4,28]*."*

However, we agree that in some places this terminology was used ineffectively, so have removed it from several lines, and replaced it with clearer descriptions. E.g. lines 140 – 142 changed from *"... a narrow band of assumed background mass-wasting...*" to "*a narrow band of mass-wasting around the expected normalised value of one*". Likewise, we have removed the word "background" from line 86/87, simplifying it from "*ASM-strength normalised background rate" to just "ASM-strength normalised rate*". We also added the timing of the monsoon-season to lines 99 – 101 for clarity:

"Mapping was conducted across 29 individual time slices, where each time slice encompassed a given year's monsoon season (approx. May – September) as well as a varying number of months either side (see methods)."

Comment 25:

Line 64: Cosmogonic should be cosmogenic

Response 25:

Corrected, now on line 49.

Comment 26:

Line 79: The definition of mass wasting associated with road building is confusing. Is the sediment produced by cutting and constructing the road recorded or is it the enhancement due to undercutting of slopes? The former is a single event while the later is a continuing process. Figures of examples of road associated mass wasting would help with these definitions

Response 26:

See comment/response 1 and comment/response 23; roads now removed from manuscript due to lack of supporting data.

Comment 27:

Line 112: ~ should be >

Response 27:

Corrected, now line 97.

Comment 28:

Line 112: The magnitude-frequencies of the mapped mass wasting events should be included as a figure to help the reader understand their statistics better, particularly as their shape is used in the methodology.

Response 28:

We have included a new figure (4), which shows the cumulative distribution functions of the area-frequency data for key selections of our inventory: 1993 event, 2002 event, the post-2015 years, and the unperturbed years.

Comment 29:

Line 134: How is the strength of a monsoon defined? Strength could be total precipitation or intensity or another measure of precipitation. I would avoid the word strength and be more specific.

Response 29:

In this case we define strength specifically as the total grid-average monsoon (May – Sept) precipitation across our study region. We have now explicitly defined this in the manuscript, making it clear that whenever the term "ASM-strength" is used, it refers to this definition of strength. See lines 122 – 130:

"We use total May – September precipitation as a proxy for local monsoon strength, as typical measures of monsoon strength such as the SASMI [39] are derived over extensive regional scales, and do not accurately capture local changes in monsoon conditions across the study region. As such, from this point forward, the term "ASM strength" is defined here specifically as total May – September precipitation across the mapped region. Of the 24 pre-Gorkha earthquake years included in this analysis, 22 years display moderate to strong exponential relationships between mass-wasting volume per unit area and total grid-averaged precipitation, with R^2 values for these years of 0.61 – 0.66 for scar volumes and 0.83 – 0.84 for total volumes (Fig. 2)."

Comment 30:

Line 138: I found the current wording of the methodology confusing. As it is currently worded it sounds like a single mean is produced of the monsoon landsliding rather than a yearly estimate. I suggest rephrasing it to: "each of the 29 ASM seasons."

Response 30:

Changed to *"…each of the 29-mapped time slices"* (now line 139 – 140).

Comment 31:

Line 153: A figure of the yearly frequency – magnitudes would help the reader interpret and understand these findings themselves.

Response 31:

We have included a new figure (4), which shows the cumulative distribution functions of the area-frequency data for key selections of our inventory: 1993 event, 2002 event, the post-2015 years, and the unperturbed years. We do not show these for all years, as having 29 separate curves is unreadable, and detracts from the comparison between the main events.

Comment 32:

Line 161: If a monsoon can produce mass wasting significantly greater than the estimated value from the normalisation it raises the question of how well the normalisation works. If this storm is as anomalous as is implied by the normalisation the manuscript should justify why it is not included in the normalisation. This would include discussion on how to identify extreme events, particularly as they seem to be exponential rather than normally distributed in nature.

Response 32:

We have added substantial new discussion r.e. extreme rainfall (see comment/responses 12 and 22). This includes more explicit discussion of why the extreme rainfall events don't fit the normalisation (lines 169 – 175), a precise definition of what is meant by an extreme storm (lines 200 – 212), the number of similar events to have occurred (lines 214 – 226), the fact that no other events had comparable spatial extents to 2002 (lines 251 – 261) and the return periods of these events (lines 263 – 270).

Comment 33:

Line 167: The use of landslide density is confusing and hides the fact that the 2002 storm was more erosive than the 1997 storm. I suggest presenting all findings in terms of volume rather than area.

Response 33:

All results now presented with volume, usually as volume/density (m^3/km^2), to account for the varying grid cell sizes used in much of the new analysis.

Comment 34:

Line 169: To combine these two events I feel the return time of the rainstorms needs to be understood. Without knowing the return period of the storms, it is unclear whether these events are frequent, and therefore important to the long-term erosion rate or insignificant. Combining them together could make them seem more significant than they are.

Response 34:

Recurrence intervals now calculated, as outlined in comment/responses 12, 22 and 32, and on lines 263 – 270.

Comment 35:

Line 175: Preconditioning is set up to be an important part of the manuscript however very little time is spent on describing it in this study. A figure showing the described correlation between earthquake magnitude and preconditioning (particularly as no mention of preconditioning prior to 2015 despite multiple >Mw6 earthquakes) would help to give more attention to this process.

Response 35:

As outlined in comment/response 11, we have added substantially more discussion and analysis on earthquake preconditioning. Due to figure limitations, we decided not to include the suggested figure, though we do add a paragraph to fully describe preconditioning (lines 273 – 288), and devote significant space to discussing why earlier $M_w > 6.0$ earthquakes in 1988 and 2011 did not induce the same short term perturbation as seen following 2015 (lines 354 – 389).

Comment 36:

Line 177: The timing of the earthquake compared to the monsoon is not clear. It should be noted somewhere that the earthquake occurred prior to the monsoon. It is also unclear whether the coseismic landslides were mapped or the Roback 2018 inventory was used.

Response 36:

Timing of Gorkha relative to the monsoon now included (line 281-282). Clarified that we did not map any coseismic events (lines 103 - 105), and that the pre-existing Roback inventory was used to look at the coseismic events (lines 402 - 405)

Comment 37:

Line 178: It is not clear why preconditioning increases mass wasting by a factor of 4 but is only the same as 2 ASMs. This should be rewritten for clarity

Response 37:

The ASM-normalised mass-wasting rate is strongly dependent on the rainfall observed in that year. So as the 2015 rainfall was very low, 2015 had a factor of 4 perturbation relative to what would have been expected given the rainfall, which equates to approx. 2 ASMs in absolute terms. To avoid this confusion, the section that included line 178 has been completely re-written, so that the normalised mass-wasting rates and ASM-equivalents are now in completely different sections (the former in lines 290 – 300, and the latter in lines 391 – 405).

Comment 38:

Line 187: Reference to figures

Line 193: Again, reference to figures

Response 38:

Equivalent lines now referenced to figure 3 (lines 290; 297).

Comment 39:

Line 196: If this were true surely the uncorrected trends would not decrease through time? They maintain a similar trend to the corrected i.e. decreasing to the background rate which implies that preconditioning is important here too

Comment 39:

This discussion hinges on what is considered to be preconditioning. If preconditioning is only considered to be new failures, then we see that the corrected rates fall immediately back to near the normal. And yes, the uncorrected rates also decay back to the normal in a few years (due to less and less reactivations), but by the strictest definition this still wouldn't be considered earthquake preconditioning, instead being due to the natural process of material slowly being transported off hillslope and/or revegetation stabilising previously unstable coseismic deposits. However, we accept this is an area that is open for debate, and highly dependent on definitions, so we have added several lines explicitly defining different potential types of preconditioning, and how our results relate to each (lines 304 - 312).

Comment(s) 40:

Line 197: The areas shown in figure 4 cannot be compared to volume therefore it is hard to compare with the other perturbations discussed

Line 201: It would be useful to know something about the amount of road construction occurring. What is the correlation between rate of road building and mass wasting? This would help clarify the definition of road associated mass wasting and forecasting into the future. Figures of images showing examples

of mass wasting associated with road construction will also help to clarify how they are related and defined.

Line 202: As area is shown in figure 4 it is now not clear whether volume or area is discussed here. I again recommend only using volume when discussing or show results.

Response(s) 40:

Roads no longer being considered, figure 4 removed.

Comment 41:

Line 207: A figure showing the absolute values of mass wasting would be useful to show how the contribution by each process changes through time.

Response 41:

Figure 4 now removed, so comparison between reactivation and road processes is no longer valid.

Comment 42:

Line 236: You have not identified the controls on these events, are these years of intense monsoon that are poorly predicted by the normalisation method or are they freak storms of abnormal intensity? It is also possible similar storms occurred without causing significant mass wasting, without any analysis on rainfall patterns in this period it is difficult to interpret these events. Without clear definitions of extreme events and their return periods it is difficult to determine whether these events are likely to change through time.

Response 42:

We add several sentences to explicitly discuss the questions raised here (lines 169 – 175), concluding that it is the latter case, with these being freak storms of abnormal intensity. We clearly define what we mean by an extreme storm (lines 200 – 212) and identify other storms that occurred without mass wasting (lines 214 – 226). This allows a more quantitative assessment of how often these events are likely to occur (e.g. recurrence intervals; lines 263 - 270).

Comment 43:

Line 261: The timing of the monsoon should be mentioned to help the reader understand why these months were chosen for imaging

Response 43:

Added (now line 459/460).

Comment 44:

Line 300: This section should be above the section discussing precipitation as it links directly to the discussion of mass wasting.

Response 44:

Moved above precipitation discussion (now line 509)

Comment 45:

Line 343: The averaging of the monsoon should be using volume rather than area. Area is not a useful measure of sediment flux due to the relationship between landslide area and volume.

Response 45:

All analysis now re-done with volume (lines 600 – 621; 624 – 662)

Comment 46:

Line 370: Here it would be useful to have a figure of the magnitude – frequencies of the years described. The reader can then made informed decisions on the applicability of this methodology.

Response 46:

See comment/responses 38 and 31.

Comment 47:

Figure 2: It would be good to have the events discussed in the manuscript highlighted in this figure. Some form of time series of precipitation would also help to understand the extreme rainfall event described.

Response 47:

Added monthly rainfall time-series to figure. Added labels for the main perturbing events.

Comment 48:

Figure 4: The "Reactivations + Road" line is hard to see. Area is difficult to compare with volume as discussed in the rest of the manuscript. Area is not important for erosion rates and should not be compared. Absolute volume values through time would be much more useful.

Response 48:

Figure now removed.

REVIEWER COMMENTS

Reviewer #3 (Remarks to the Author):

Review of revised Jones et al 2020 Nature Communications paper
Oliver Francis
This revised manuscript has tackled the major comments I raised in the previous round of reviews. The manuscript quantifies the impact of the Asia Monsoon on the Himalaya via an in-depth study of a 30 year long mass wasting inventory. Quantifying the mass wasting associated with the Monsoon has allowed the authors to identify the contribution of extreme rainfall and earthquake preconditioning to erosion rates – the first study to do so in the Himalaya. Alongside this they have identified a potential topographic signature of long-term earthquake preconditioning in the highest and steepest parts of the mountain range. I have some minor comments and suggestions for the authors, primarily on clarifying some of the terminology and definitions they have used. I have summarised the main points in the paragraphs below and listed the other points with line number at the end of this document.
I found the terminology used to describe the various mass wasting volumes (Total mass wasting volume, Corrected mass wasting volume, and Scar volume) used in this paper confusing. In the methodology section (lines 513 – 514) Scar area (or volume) is described as the result of a correction of the total landslide polygons to remove the influence of long runout landslides. This leads to confusion when discussing the Corrected mass wasting volume, which is just the total mass wasting volume minus the mass wasting associated with reactivating previous deposits. I suggest renaming the "Corrected mass wasting" to a term which closer describes the process by which it is produced, perhaps "New mass wasting" or "non-reactivating mass wasting".
I also would like to see some uncertainty analysis undertaken on the definition of an extreme rainfall event. Currently the authors use a threshold of 1.8x the mean annual 24-hour total of precipitation of a cell to define an extreme event based upon the 2002 rainfall event. This threshold could be too high for other unrecognised events which trigger abnormal volumes of landsliding. A possible method of quantifying the uncertainty in the precipitation threshold used could be via analysing the mass wasting inventory. A threshold for an extreme mass wasting event could be defined and compared to the precipitation threshold required to produce it, any unpredicted extreme mass wasting events can then be quantified and used as uncertainty for the precipitation threshold.
Minor Comments
Line 112: Does the correct volume only apply to mass wasting produced by mass wasting?
Line 123: What are the units of the Monsoon Strength?
Line 136: Which mass wasting volume is used to define the ASM strength normalised mass wasting?
Line 175: How well do the locations of the rainfall event and the 2002 mass wasting correlate? (reference figure 5)
Line 181: Make it clearer that you believe the extra mass wasting is due to case 2 (170) i.e. A localised rainstorm not recorded in the ASM strength
Line 197: It is worth highlighting the 1993 event is not recorded in the RX1 data, so the precipitation threshold is based exclusively on 2002
Line 203: How are the cells related to the rainfall event identified?
Line 229: Perhaps fits would be a better way to describe the equations
Line 235: Across the entire mapped region and period
Line 341: It is not clear why ridges are discussed and then the concept of excess topography is brought up
Line 342: When are these changes relatively to? Between two inventories?
Line 372: The 1988 earthquake had a maximum PGA of 0.28g
Line 443: You could mention your finding of the requirement of a recovery time of an area after an extreme mass waste event as this is the opposite of the earthquake preconditioning. i.e. An event reduces future mass wasting
Line 456: How were landslides mapped in 2013? Were new landslides assumed to occur in 2013 or discounted?
Line 537: It would be useful to have a quick definition of how reactivated mass wasting was identified

within the methods.
Line 584: What are the units for the x-min?
Line 592: Quote the X-min of Marc et al and Roback et al.
Line 632: A complete list of the perturbed years rather than a hyphen would be clearer (2000, 2001, 2002, 2003, 2004)
Figure 1: Does this figure include the Coseismic mass wasting?
Figure 3: The left Y axis (ASM Normalised Mass wasting rate) could start at 0 and the Earthquake Magnitudes could be displayed on the left for clarity
Figure 4: The symbols in the legend should be larger to see the colours easier

Review of Jones et al,

The author present a new long term, multitemporal catalogue of landsliding in a substantial fraction of the Nepal Himalayas. Specifically they map about 30 years of annually resolved landsliding, that they use to discuss how monsoon strength, extreme rainfall and earthquake drives the regional landsliding. The methodology follow previous works (Marc et al 2015, 2019) in attempting to calibrate a relation between monsoon strength (estimated by satellite) and total landslide volumes, and then quantify deviation from this relationship to highlight the influence of earthquake (by focussing on the monsoon after the Gorkha earthquake) and extreme rainfall. After these analysis the author attempt to do estimate the relative erosion caused by the extreme rainfall and earthquake trigger relative to the annual monsoon.

I note that the manuscript has been drastically changed based on previous referees comments, so that one control effect (road) has been removed, while entirely new analysis on extreme rainfall and the role of past earthquake have been added. I appreciate clarification has been added about the methods and implications, but still I have several questions that may be important both on the old and new analysis.

As of now the key result is the identification of an empirical relationship between monsoon rainfall and annual landsliding. This allows to better constrain the impact of the earthquake, although the authors do not add much on this from Marc et al 2019 I would say, except that reactivation/remobilization (unclear) may recover over a bit more time than new landslides, as expected from the literature (Wenchuan EQ see Fan et al 2019, Chi-Chi EQ see Marc et al 2015 vs Hovius et al 2011).
The control for extreme rainfall and is likely an interesting result but is needing some clarification/extra analysis to be entirely convincing and quantitative. A key issue is that the author use diverse satellite rainfall (with severe limitations in steep topography), one for the long term monsoon strength and one for the extreme rainfall.
The budgeting of the different erosion factor is rather weak in my opinion and also likely need some clarification/updated analysis.

So overall, I think the work is a nice prolongation of Marc et al 2015/2019 on its first part (landslide mapping, monsoon forcing, EQ perturbation) although it cannot be published in this current form as substantial revisions and clarifications are needed. Then the paper is spreading in several directions with the newly added analysis (on extreme rainfall and EQ budget) and may need either substantial work to be at the level of the existing literature or need to be left for later.

My recommendation would be to focus on the new results that should be very valuable to the earth surface/natural hazard community (the MIL/ERIL scaling and the EQ preconditioning as a function of PGA/topo) and leave for future work claims about the sediment budget (between MIL/ERIL/EQIL) that are currently quite poorly analyzed.

Below I detail the major issues I have, a few minor issues and a list of line by line comment/suggestions.
In this review I used frequently the term MIL, EQIL and ERIL for Monsoon, Extreme Rainfall and EQ induced landslides, respectively.

Sincerely, do not hesitate to contact me if needed

Odin Marc

Major issue
1) Comparison of MIL and ERIL :

I think the approach of the author (triggered by the revisions and not planned) is problematic for several reasons.

1.1) The authors use Persiann to define the background MIL (in the form of a relation between total landsliding and the total rainfall over 5 months), because of its long term availability and stability. But then given that it is consider inadequate to capture extreme rainfall they use TRMM to constrain anomalous years.

I do believe that 1993 and 2002 are likely due to additional extreme events, as reported in the literature and as captured by TRMM for the 2002 events. However, I have many doubts about their following analysis.

Thus several suggestions come to me : Why to use non-overlapping product with different rainfall retrieval methods… At this point using Aphrodite raingage product (Yatagai et al 2012) may be better : It was shown by Andermann et al 2011 to be likely the most reliable rainfall product across the Nepal Himalayas. So potentially it could solve the author problem, by giving them a prediction as Vtot = Rmsn + Rextreme, from 1988 to 2015. Indeed with 0.25° daily resolution from 1951 to 2015 Aphrodite has the potential to capture daily extreme and long term trends. (True in this case the “post-seismic” in 2016-2018 should be calibrated with another dataset… This seems less problematic to me as better satellite data exist toward the present (and could be blend with Aphrodite maybe) and as to me the true novelty of the work is not in the post seismic calibration as much as in the meteorological calibration including MIL and ERIL)

I show below that the data on Landslide scar volume provided by the author does match monsoon rainfall for most year (especially after removing the largest landslide see comment below). 1993, 2002 and 2015 are outlier. Some other years too. On top of that, I show below rainfall maps from Aphrodite for the 2002 and 1993 event date given by the author which confirm that these events were captured by Aphrodite.

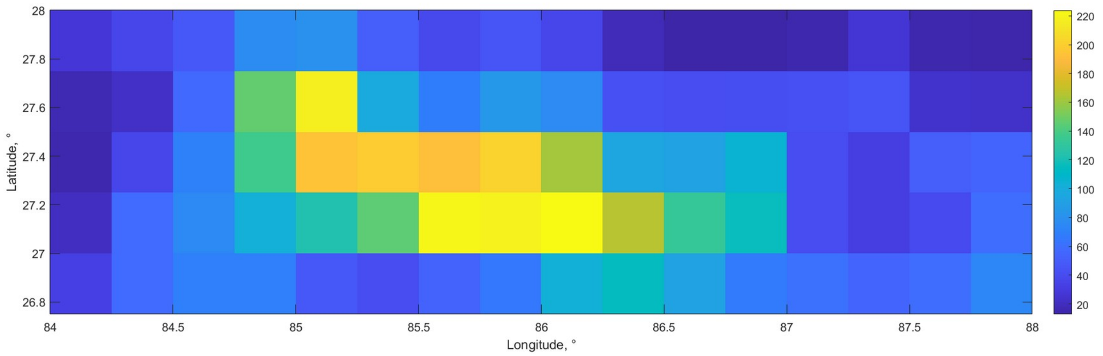

Rainfall map in mm for July 20+July 20 of 1993, from Aphrodite product. Both days had several gridcell >100 mm/day. Extent roughly similar to Fig 1 and 5 from the authors.

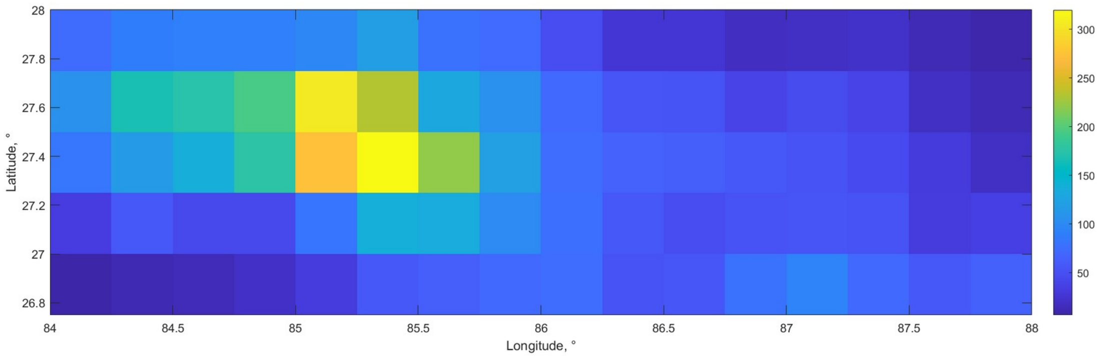

Same as above for the 23 of July 2002. Several gridcell have > 250 mm/day.

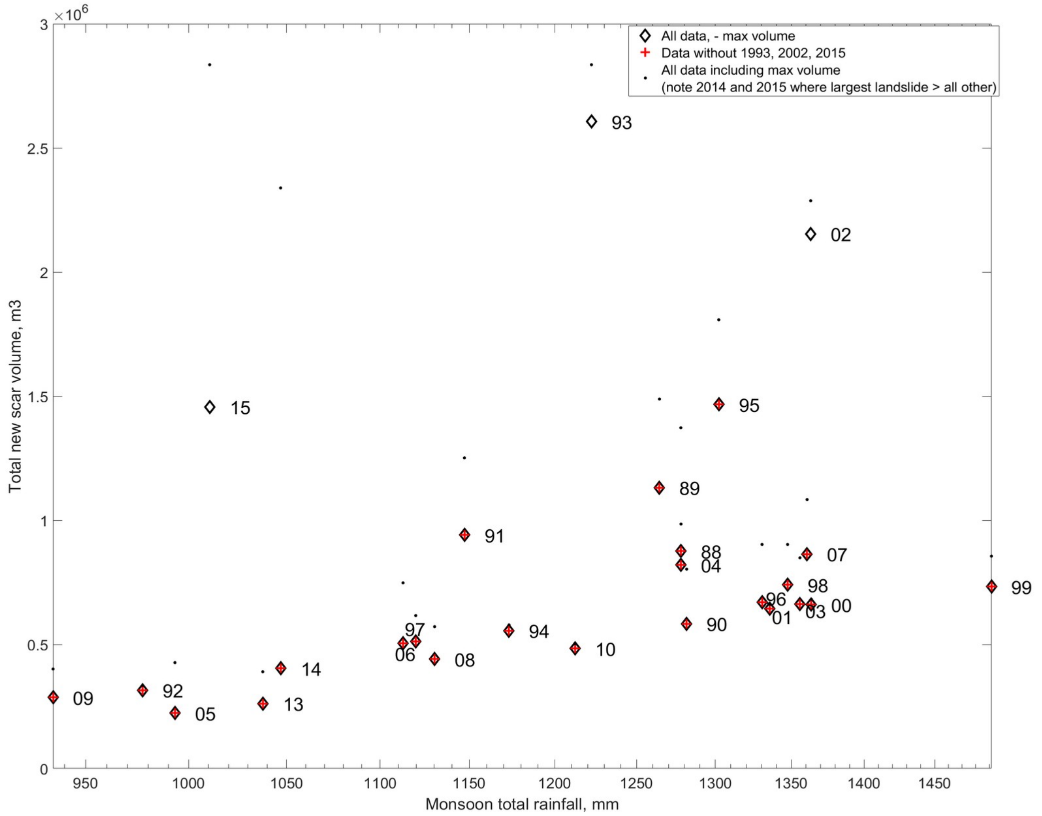

Figure : Monsoon total (May September in a polygon similar to mapping extent from Fig 1) from Aphrodite product from 1988 to 2015. 2015, 1993 and 2002 are strong outliers. However, 95 is another likely one and possibly 89 and 91… Of course the May-Sep total may not be the best index, see comment later. Also note how Persiann substantially underestimate rainfall with monsoon of 600-1100 mm

Anyway, Aphrodite seems to be the best, unique product with which the author could solve the MIL and ERIL respective influence, without introducing bias from different sensor and algorithm. It could also allow a unique treatment of extreme from 1988 to 2015, based on gridded daily rainfall.

1.2) It is not clearly explained how the authors jump from a whole area/ whole monsoon landslide mean to an analysis that relies on localized grids (for the ERIL and also EQ long-term preconditionning analysis). If they compare the landslide density in one given gridcell in one year to the 29 long term mean, or even the density predicted in average for this year it seems inadequate !! Because it is clear that the density varies a lot spatially during their 29 period, so they would need to account for that. Could not the author show that the deviation from Monsoon forcing (their Fig 2, My Figure above) relate to how strong and widespread was extreme rainfall in some years ?

1.3) Are the author sure that the May-Sep total rainfall is the best predictors ? Previous work (eg Gabet et al 2004) has suggested that it was only after the first weeks of the monsoons that landslide started. And likely that only intense rain burst after the hillslopes had received sustain rainfall for some weeks (and reached some state of partial saturation) were effective in triggering…
I think the author should double check if other basic indicator are not better at explaining their total landsliding (See Suppl Fig 4 of Marc et al for a selection of meaningful index).
Some (brief) discussion/ evaluation of this would be useful.

2) ERIL quantification and their long-term budget:
The definition of extreme rainfall and their recurrence time is fairly naive and with some inconsistencies. In part because they use short time series (Aphrodite would help), but not only.
For example, why do the author keep a mixed threshold of 200 mm and 80 % increase (L212) ? Also a % increase relative to the mean is not telling you how much "extreme" is an event without knowing its standard deviation… An Z-score or anomaly (i.e. [X -mean(X)]/Sigma(X)) would be the qualitative minimum...
But more generally the standard way to look at extreme value is through the (Generalized) Extreme Value theory (e.g., Overeem et al 2009, Saito et al 2012, Marc et al 2019b), or through the recent framework of the Metastatistical Extreme Value distribution (see Zorzetto et al 2015). In both framework in each grid cell the author could have fit a GEV or MEV distribution to the daily rainfall data of Aphrodite over the last 30 years… With this the author could define quantitative recurrence interval for diverse daily intensity across their study area and thus "how extreme" were the event in 1993 and 2002.
I think these quantification are needed to be consistent with the literature on extreme rainfall and to adequately estimate their budget relative to background MIL (especially through their recurrence time that may not be the same for 1993 and 2002).

3) EQ contribution to long-term budget:
I think the conclusions of the author (EQIL is negligible relative to rainfall) is an inference based on several mistakes done while comparing MIL and EQIL. I suspect that unless extending substantially the paper (with new methods and discussion), the authors may rather want to drop this part and limit themselves to rainfall control and preconditionning.

3.1) Clearly the long term budget of erosion due to EQ is not only due to the recurrence of one type of EQ, but to every EQ causing substantial ground shaking (so from Mw ~6 to 9 in the Himalayas, as paleo landslide data suggest (Schwanghart 2016). The approach of suming the impact of various EQ size is classic (Keefer 1994) and was applied in Nepal with substantially more care (Marc et al 2019), so if the author want to bring anything useful they cannot just show the impact of Gorkha and its recurrence time.

Further, the author neglect the aspect that MIL and EQIL may also have a different potential to trigger very large/ deep landslides.

3.2) EQ affected area vs monsoon affected area : Given your zone of interest is much larger than the EQ affected zone (despite imagery very few landslides occurring the Siwaliks and in the most eastern part of your mapping areas, where your fig 1 show a lot of the total monsoon induced mapping.) you are bound to underestimate the ratio EQIL/MIL … Possibly by almost a factor 2.

4) EQ long term preconditionning
Here my main interrogation is about the method :
L331 : We then plot these summed PGAs against each grid cell's 2015 monsoon season percentage change in mass-wasting volume density (m^3/km^2) (Fig. 7a – b)."
I am not sure what the authors did, as for the approach to spatialize ERIL (see comment 1.2). L216 they say they compare in each cell the total landsliding normalized by mean landsliding. Does it mean they do not account for a meteorological forcing, and the fact that strong monsoon should cause above average landsliding ? Or they also find a scaling at the 0.25° scale ? This should be clarified !

Also I think the author approach of coupling PGA and Excess topography (which essentially gives how frequent (and steep) are slopes in each gridcells) may be quite promising but I still think several clarification are needed :

Fig 7 (and S1) should plot the Excess mass wasting VS Excess Topography (NOT the opposite as it is now), so that knowing PGA and Topography, mass wasting can be predicted. This will change the R2 and I recommend the author to check if the best relationship is linear or not (log log plot) and recommend that they check if summing PGA above a threshold (as often PGA<= 0.1-0.2 g do not cause landslide and may not cause preconditionning, see Meunier et al 2007, Yuan et al 2013) improve their scaling.

Also it is not clear what they mean by weighted (From the values, it seems they simply did the product of PGA and topography). If so they should rather say that.
Last the author should comment on 1) potential cross-correlation between PGA and Topo from diverse EQ, 2) The influence of various threshold angle (the one they used is not even given in the methods !).

Additional Line By Line comments

L96 visual inspection ?

Results
L128: "22 years display moderate to strong exponential relationships between mass-wasting volume per unit area and total grid-averaged precipitation, with R^2 values for these years of 0.61 – 0.66 for"
I think the wording is slightly ambiguous: what about something like : we found that mass-wasting volume per unit area is increasing total grid-averaged precipitation

L142: "and post 2015". I would write out and add:
"Specifically, if coseismic reactivation are considered, the monsoon 2015-2017 are above the expected monsoon scaling, while considering only new failures, only the monsoon 2015 is anomalous, as reported by Marc et al 2019."

L298: In 2017 you state landslide was perturbed by 1.5-1.7 but with their uncertainties (on the annual volume) they are largely overlapping with the monsoon scaling 1 sigma…

L304 -L308: I agree with the author that both reactivation and new landslides are important for he post-seismic evolution of hazard but do not think they should discuss the "definition" of preconditionning. They preconditionning (as it name suggest) means the "conditions" of the landscape as changed so that future landslides may be more of less likely in (part of) the landscape. If it is due to strong shaking induced damage, there should be more NEW landslides. The part due to coseismic landslide reactivation (need to be more clear about what you did in the methods see my other comment below) is not necessarily landslide, but may simply be loose sediment transport during the monsoon. Several issue should be clarified here : it may not have the landslide A-V scaling so depending what you mapped your reactivation V my be a bit overestimated. Then it is relevant for hazard and sediment evacuation timescales, but probably not erosion as it does not create new loose sediments (just represent how much coseismic landslide deposit reach channel and in how much time).

L308: I disagree, here you mix too different type of measurement : ChiChi measurement in Marc et al 2015 is >90% new landslides, therefore it represents the recovery of the new landslide susceptibility, not the end of the abnormal remobilization of coseismic slides. So the ChiChi time should be compared to the ~6-14 months of Gorkha (the time from the EQ to the end of the first monsoon or the start of the next). If you want to compare your 2 years recovery (including remobilization), it may be better compared to the time of abnormal fluvial sediment export of 6-8 years from Hovius et al 2011 (which will include new and remobilized landslide as long as their sum is substantial relative to background landsliding).

I would try to state clearly the difference in this two concept (increased landslide susceptibility and increased landslide remobilization). And relate them to the relevant literature discussion.
On this you should also cite Dahlquist and West which showed the extra rainfall induced new debris flow was anomalous in 2015 only, whereas coseismic remobiliztions of landslides into debris flow was still substantial in 2016.

L320: "less well understood" … Not sure the short term preconditionning I better understood, but it is more frequently observed. So I would rather say that.

L321-322 : You suggest only large EQ can mobilize deep landslides but this is not so simple when you look at data either in Nepal or elsewhere, where very deep landslides can also be mobilized during rainfall (eg Morakot or Talas typhoon in Taiwan/Japan, see Marc et al 2018)

L325-327 : Why do you consider all these EQ for their "preconditionning impact" and not for their coseismic impact and thus for the long-term budget of your conclusions ?
Also you are contradicting yourself : you said long preconditionning may affect EQIL so you should look at Gorkha coseismic landslides (NOT the MIL) and see if they are better predicted when also considering past EQ shaking, like what they did in Parker et al., 2015.

L345 : you report $R2 = 0.34$ in text but $R2= 0.377$ in Figure S1. Also this figure should plot Excess mass wasting VS Excess Topography (NOT the opposite as it is now) and then show the R2 …

Conclusions:
L419 : I am surprised by this recurrence time. In Marc et al 2019 (see details there) we concluded to a 300 yrs return for Mw 7.9. Based on Bollinger et al 2014 but also the observed Gutenberg Richter from Avouac, 2015. Anyway, see my major comments but the EQ budget need to be substantially updated or abandonned.

Methods
L465 : it can be pansharpened with pan chromatic imagery at 15m resolution (This is not a technically a resampling as it is an extra layer of different data.)

L474: "All types of rainfall triggered mass wasting"
Unclear and the reference is not helping. Do you mean not only landslides but also say, rilling, debris flow ? Or do you mean mapped landslides were not differentiated by types ? Or something else ?

L481: "fully within existing mass-wasting" is a bit confusing; Do you mean that you mapped textural changes within active landslides, for which the responsible process is likely unclear and A-V scaling unconstrained. Or do you refer to the scarring of a revegetated previously mapped landslide ? In this second case it has more chance to be a proper landslide, and a reactivation too, but I would phrase: "scarring/vegetation disturbance within the boundary of previously mapped landslides were considered reactivation." something like that.
See Major comment fro the required clarification between Reactivation and Remobilization.

L499 : I am not sure this prove that the difference in mapping duration is not affecting your result, given obviously the primary control will be ASM rainfall. I appreciate your effort but if you want to support this claim I think the figure should show that there is no correlation between the non monsoonal number of months (ideally the non monsoonal amount of rainfall in case some year had extra monsoonal substantial precipitation) and the deviation from your landslide volume / Monsoon rainfall relationship.

L507 : or roads ? From the rebuttal I had the impression roads related slides where mapped separately and then not included in this version, it is not reflected in this sentence.

L537 : "it is the only accessible precipitation product with a spatial resolution of at least 0.25o by 0.25o that spans our required time period of 1988 – 2018"
This is simply not true. At least the two following product MSWEP (Beck et al 2017) and Aphrodite (Yatagai 2012) cover the period and have the resolution you want. I do not think you have any reason to use Persiann, which by only using infrared measurement is severely limited (see conclusions in Beck et al 2017).

L559: Ok but others product are also made for that, possibly with less issues (for daily rainfall) see Yatagai 2012, Beck et al 2017.

L619-621 : You argue you can remove the Jure landslide because of progressive failure. I agree but it may not be the only large landslides that may be decoupled from the monsoon. In Marc et al 2019, several other landslides were shown to be anomalous relative to the monsoon in which they occurred, and this was for only a few years…. So clearly your analysis may have several years disturbed by

exceptional landslides, and removing only Jur may not be adequate…. In the figure above I show the simplest correction that is to remove the largest scar volume in each year… In Marc et al 2019 a threshold for the ratio between the largest and second largest scar area seemed to be able to discriminate suspicious slides, and could be reused here.

Figure 2: Why not putting all years, including Post EQ ? This should allow to show which years are within the PreEQ fit and which are not. The 1Sigma uncertainties should appear in this figure too I would say, and if possible in the fit equation.

Corrected and Uncorrected terms are not ideal terms are it sounds like one is true and the other false whereas they represent different things. I would favor something like: New and New + Reactivation/Remobilization.

Figure 4 : I would recommend to show PDF curves rather than CDF. Because they can be better compared to previous work, especially in Marc et al 2019. Though the fact you find a power-law decay of ~2.5 is consistent with the higher resolution inventory from Marc et al 2019 so it is reassuring.

References used in this review, not present in the paper:

Andermann, C., Bonnet, S., & Gloaguen, R. (2011). Evaluation of precipitation data sets along the Himalayan front. *Geochemistry, Geophysics, Geosystems*, *12*(7), Q07023. https://doi.org/10.1029/2011GC003513

Avouac, J.-P.: From Geodetic Imaging of Seismic and Aseismic Fault Slip to Dynamic Modeling of the Seismic Cycle, Annu. Rev. Earth Pl. Sc., 43, 233–271, https://doi.org/10.1146/annurev-earth-060614-105302, 2015.

Beck, H. E., Vergopolan, N., Pan, M., Levizzani, V., van Dijk, A. I. J. M., Weedon, G., et al. (2017). Global-scale evaluation of 23 precipitation datasets using gauge observations and hydrological modeling. *Hydrol. Earth Syst. Sci. Discuss.*, *2017*, 1–23. https://doi.org/10.5194/hess-2017-508

Dahlquist, M. P., & West, A. J. (2019). Initiation and Runout of Post-Seismic Debris Flows: Insights From the 2015 Gorkha Earthquake. *Geophysical Research Letters*, *46*(16), 9658–9668. https://doi.org/10.1029/2019GL083548

Fan, X., Scaringi, G., Korup, O., West, A. J., Westen, C. J. van, Tanyas, H., et al. (2019). Earthquake-Induced Chains of Geologic Hazards: Patterns, Mechanisms, and Impacts. *Reviews of Geophysics*, *57*(2), 421–503. https://doi.org/10.1029/2018RG000626

Gabet, E. J., Burbank, D. W., Putkonen, J. K., Pratt-Sitaula, B. A., & Ojha, T. (2004). Rainfall thresholds for landsliding in the Himalayas of Nepal. *Geomorphology*, *63*(3–4), 131–143. https://doi.org/10.1016/j.geomorph.2004.03.011

Keefer, D. K. (1994). The importance of earthquake-induced landslides to long-term slope erosion and slope-failure hazards in seismically active regions. *Geomorphology*, *10*(1–4), 265–284. https://doi.org/10.1016/0169-555X(94)90021-3

Marc, O., Gosset, M., Saito, H., Uchida, T., & Malet, J.-P. (2019). Spatial Patterns of Storm-Induced Landslides and Their Relation to Rainfall Anomaly Maps. *Geophysical Research Letters*, *46*(20), 11167–11177. https://doi.org/10.1029/2019GL083173

Meunier, P., Hovius, N., & Haines, A. J. (2007). Regional patterns of earthquake-triggered landslides and their relation to ground motion. *Geophysical Research Letters*, *34*(20), L20408. https://doi.org/10.1029/2007GL031337

Overeem, A., Buishand, T. A., & Holleman, I. (2009). Extreme rainfall analysis and estimation of depth-duration-frequency curves using weather radar. *Water Resources Research*, *45*(10). https://doi.org/10.1029/2009WR007869

Saito, H., & Matsuyama, H. (2015). Probable Hourly Precipitation and Soil Water Index for 50-yr Recurrence Interval over the Japanese Archipelago. *SOLA*, *11*(0), 118–123. https://doi.org/10.2151/sola.2015-028

Schwanghart, W., Bernhardt, A., Stolle, A., Hoelzmann, P., Adhikari, B. R., Andermann, C., Tofelde, S., Merchel, S., Rugel, G., Fort, M., and Korup, O.: Repeated catastrophic valley infill following medieval earthquakes in the Nepal Himalaya, Science, 351, 147–150, https://doi.org/10.1126/science.aac9865, 2016.

Yatagai, A., Kamiguchi, K., Arakawa, O., Hamada, A., Yasutomi, N., & Kitoh, A. (2012). APHRODITE: Constructing a Long-Term Daily Gridded Precipitation Dataset for Asia Based on a Dense Network of Rain Gauges. *Bulletin of the American Meteorological Society*, *93*(9), 1401–1415. https://doi.org/10.1175/BAMS-D-11-00122.1

Yuan, R.-M., Deng, Q.-H., Cunningham, D., Xu, C., Xu, X.-W., & Chang, C.-P. (2013). Density Distribution of Landslides Triggered by the 2008 Wenchuan Earthquake and their Relationships to Peak Ground Acceleration. *Bulletin of the Seismological Society of America*, *103*(4), 2344–2355. https://doi.org/10.1785/0120110233

Zorzetto, E., Botter, G., & Marani, M. (2016). On the emergence of rainfall extremes from ordinary events. *Geophysical Research Letters*, *43*(15), 8076–8082. https://doi.org/10.1002/2016GL069445

Responses to Reviewers

We would first like to thank both reviewers for their detailed and comprehensive comments which have improved the quality of this manuscript. The below paragraphs outline our responses to their suggestions. Throughout the responses and in the comments on the track changes comment, we use the abbreviations R4 and R3 to refer to each reviewer.

Reviewer 4.

Major Comments

1: Comparisons of MIL and ERIL.

1.1) Comment: A summary of this comment is: To use the APHRODITE data product as this provides a long-term daily record of precipitation that is considered to be the most accurate over the Himalayas. This would allow a comparison between MIL and ERIL without worrying about bias/differences between rainfall products. Another product could be calibrated and added to the end of APHRODITE to get the post-2015 period.

Response: As suggested by the R4, we have now repeated our normalisation analysis using the APHRODITE product from 1988 – 2015. However, we do not attempt to combine another rainfall product with APHRODITE for the post-2015 period. If the reason for using APHRODITE is to avoid bias/uncertainty when comparing MIL to ERIL, then combining another dataset with APHRODITE for the post-2015 period seems equally uncertain. In our opinion, it would be difficult to have any certainty in the timescales and magnitudes of post-earthquake changes (which is a key element of this project) if another data set was used within the APHRODITE series. As such, we opt to keep our PERSIANN based normalisation to show give a more time-stable assessment of post-2015, and use the APHRODITE data to corroborate the results of PERSIANN for pre-2015, and allow a non-biased comparison between MIL and ERIL for the pre-2015 period.

Action: Normalisation analysis repeated using APHRODITE (see section titled, Empirical relationships between the ASM and mass-wasting, lines 101 – 148). Furthermore, full justification of our use of rainfall data is given in the methods section, on lines 526 – 579.

1.2) Comment: It is not clearly explained how the authors jump from a whole area/ whole monsoon landslide mean to an analysis that relies on localized grids (for the ERIL and also EQ long-term preconditioning analysis). If they compare the landslide density in one given gridcell in one year to the 29 long term mean, or even the density predicted in average for this year it seems inadequate !! Because it is clear that the density varies a lot spatially during their 29 period, so they would need to account for that. Could not the author show that the deviation from Monsoon forcing (their Fig 2, My Figure above) relate to how strong and widespread was extreme rainfall in some years ?

Response: As suggested by R4, we now approach ERIL on a regional scale. Specifically, using Z-score anomalies and the APHRODITE dataset, we count how many days within each monsoon-season experienced extreme rainfall comparable to that observed in 1993 and 2002, and correlate this with the normalisation results from Fig. 3.

Action: Lines 201 – 249 in the section "Impacts of extreme rainfall" outline how we use a regional approach to ERIL. Fig 5 is a new figure that shows the number of days within each monsoon season that experienced extreme rainfall correlated with the APHRODITE normalisation in Fig. 3b. This clearly shows that there is a correlation between the perturbations from the normal and extreme rainfall.

1.3) Comment: Are the author sure that the May-Sep total rainfall is the best predictors ? Previous work (eg Gabet et al 2004) has suggested that it was only after the first weeks of the monsoons that landslide started. And likely that only intense rain burst after the hillslopes had received sustain rainfall for some weeks (and reached some state of partial saturation) were effective in triggering…I think the author should double check if other basic indicator are not better at explaining their total landsliding (See Suppl Fig 4 of Marc et al for a selection of meaningful index). Some (brief) discussion/ evaluation of this would be useful.

Response: Based on Suppl Fig 4 of Marc et al. and a recent paper by Munoz-Torrero Manchado et al., (2021), we now consider four indicators of rainfall (including only rainfall > 25 mm, and only rainfall after 25th June to consider the issues of saturation raised by the reviewer.

Action: This is now evaluated and discussed on lines 115 - 125, Fig 2, supplementary materials Fig S1 – 3).

2: ERIL quantification and their long-term budget

2) Comment: The definition of extreme rainfall and their recurrence time is fairly naive and with some inconsistencies. In part because they use short time series (Aphrodite would help), but not only. For example, why do the author keep a mixed threshold of 200 mm and 80 % increase (L212) ? Also a % increase relative to the mean is not telling you how much "extreme" is an event without knowing its standard deviation… An Z-score or anomaly (i.e. [X -mean(X)]/Sigma(X)) would be the qualitative minimum... But more generally the standard way to look at extreme value is through the (Generalized) Extreme Value theory (e.g., Overeem et al 2009, Saito et al 2012, Marc et al 2019b), or through the recent framework of the Metastatistical Extreme Value distribution (see Zorzetto et al 2015). In both framework in each grid cell the author could have fit a GEV or MEV distribution to the daily rainfall data of Aphrodite over the last 30 years… With this the author could define quantitative recurrence interval for diverse daily intensity across their study area and thus "how extreme" were the event in 1993 and 2002. I think these quantification are needed to be consistent with the literature on extreme rainfall and to adequately estimate their budget relative to background MIL (especially through their recurrence time that may not be the same for 1993 and 2002).

Response: As mentioned above, we now change our approach to quantifying ERIL based on the R4s suggestions. Using the full APHRODITE series, we calculate Z-score anomalies for all monsoon days across every gridcell of our study region. This is then used to define the extremity in 1993 / 2002, identify other similarly extreme rainfall occurrences and correlate these with our normalisation results. We opted not to undertake more advanced extreme values analysis, as we consider this beyond the scope of the paper, given its larger focus on earthquake preconditioning, and consider Z-scores sufficient for the conclusions that we are making. However, we agree that future work considering extreme value theory in more detail would be interesting.

Actions: Z-score anomaly analysis undertaken (see lines 201 - 249 in the section "Impacts of extreme rainfall", and Fig 5).

3: EQ contribution to long-term budget

Response: As suggested by the reviewer and editor, we have dropped this part of the analysis from the manuscript.

4: EQ long term preconditioning

4.1) Comment: Here my main interrogation is about the method :L331 : We then plot these summed PGAs against each grid cell's 2015 monsoon season percentage change in mass-wasting volume density (m3/km2) (Fig. 7a – b)." I am not sure what the authors did, as for the approach to spatialize ERIL (see comment 1.2). L216 they say they compare in each cell the total landsliding normalized by mean landsliding. Does it mean they do not account for a meteorological forcing, and the fact that strong monsoon should cause above average landsliding ? Or they also find a scaling at the 0.25° scale ? This should be clarified !

Response: We have clarified our methods for this section to explain that we do not explicitly account for meteorological forcing on a grid by grid scale. However, the % change in 2015 mass-wasting is based on each grids mean calculated from non-perturbed years only. I.e. any % increases are relative to the mean from typical ASM seasons only. As we know from our rainfall analysis that 2015 had no extreme rainfall, we thus have some confidence that 1) any significant % increase in 2015 mass-wasting is unusual relative to a normal monsoon, and 2) that any significant % increases are not attributable to either the monsoon (which we know was average to weak in 2015) or extreme rainfall.

Action: Lines 310 – 397 in the main text are completely re-written for clarity. We also add a new section in the methods titled "Percentage change in mass-wasting" (Lines 666 – 677), which provides further details on our methodology, highlighting that whilst this grid-scale approach does not consider meteorological forcing as robustly as on the regional scale we are confident that it shows a true increase not attributable to rainfall.

4.2) Comment: Also I think the author approach of coupling PGA and Excess topography (which essentially gives how frequent (and steep) are slopes in each gridcells) may be quite promising but I still think several clarification are needed: Fig 7 (and S1) should plot the Excess mass wasting VS Excess Topography (NOT the opposite as it is now), so that knowing PGA and Topography, mass wasting can be predicted. This will change the R2 and I recommend the author to check if the best relationship is linear or not (log log plot) and recommend that they check if summing PGA above a threshold (as often PGA<= 0.1-0.2 g do not cause landslide and may not cause preconditionning, see Meunier et al 2007, Yuan et al 2013) improve their scaling.

Response/action: Fig. 7 and supplementary Figs. S4- 8 are changed as recommended to show excess mass-wasting vs PGA*ET. We can confirm that the best-fit was linear, not log-log (we had tried this previously). As shown in the Supplementary Figs. S6 - 7, and outlined on lines 336 – 340, summing PGA only above 0.1 and 0.2 g does not improve the scaling.

4.3) Comment: Also it is not clear what they mean by weighted (From the values, it seems they simply did the product of PGA and topography). If so they should rather say that. Last the author should comment on 1) potential cross-correlation between PGA and Topo from diverse EQ, 2) The influence of various threshold angle (the one they used is not even given in the methods !).

Response/actions: The weighting was the product of PGA and excess topography, this is now explicitly stated (lines 329 - 330). The analysis is also now repeated with several excess topography thresholds (see lines 327 – 328 and lines 334 – 336 in the main text,_lines 683 – 685 in the methods, and Supplementary Figs. S4-5). We were unsure exactly what the reviewer meant by "cross-correlation between PGA and topo from diverse EQ", but we do now consider earlier earthquakes in 1934, 1988 and 2011 and their relation to excess topography in this analysis (lines 360 – 391 in the main text, "Impacts of earthquakes" section).

Line by line comments

Comment 1) L96 visual inspection ?

Response: Changed, line 91.

Comment 2) L128: "22 years display moderate to strong exponential relationships between mass-wasting volume per unit area and total grid-averaged precipitation, with R2 values for these years of 0.61 – 0.66 for" I think the wording is slightly ambiguous: what about something like : we found that mass wasting volume per unit area is increasing total grid-averaged precipitation

Response: Changed, lines 127 – 128.

Comment 3: L142: "and post 2015". I would write out and add: "Specifically, if coseismic reactivation are considered, the monsoon 2015-2017 are above the expected monsoon scaling, while considering only new failures, only the monsoon 2015 is anomalous, as reported by Marc et al 2019."

Response: Changed, lines 143 – 146.

Comment 4: L298: In 2017 you state landslide was perturbed by 1.5-1.7 but with their uncertainties (on the annual volume) they are largely overlapping with the monsoon scaling 1 sigma…

Response: Changed, lines 277 – 278.

Comment 5: L304 -L308: I agree with the author that both reactivation and new landslides are important for he postseismic evolution of hazard but do not think they should discuss the "definition" of preconditionning. They preconditionning (as it name suggest) means the "conditions" of the landscape as changed so that future landslides may be more of less likely in (part of) the landscape. If it is due to strong shaking induced damage, there should be more NEW landslides. The part due to coseismic landslide reactivation (need to be more clear about what you did in the methods see my

other comment below) is not necessarily landslide, but may simply be loose sediment transport during the monsoon. Several issue should be clarified here : it may not have the landslide A-V scaling so depending what you mapped your reactivation V my be a bit overestimated. Then it is relevant for hazard and sediment evacuation timescales, but probably not erosion as it does not create new loose sediments (just represent how much coseismic landslide deposit reach channel and in how much time).

Response: Reference to preconditioning definition removed. Lines 252 - 256 now clearly state the difference between preconditioning and remobilisation. Issues relating to A-V scaling and remobilisation are also now mentioned explicitly in the methods on lines 519 – 523.

Comment 6: L308: I disagree, here you mix too different type of measurement : ChiChi measurement in Marc et al 2015 is >90% new landslides, therefore it represents the recovery of the new landslide susceptibility, not the end of the abnormal remobilization of coseismic slides. So the ChiChi time should be compared to the ~6-14 months of Gorkha (the time from the EQ to the end of the first monsoon or the start of the next). If you want to compare your 2 years recovery (including remobilization), it may be better compared to the time of abnormal fluvial sediment export of 6-8 years from Hovius et al 2011 (which will include new and remobilized landslide as long as their sum is substantial relative to background landsliding). I would try to state clearly the difference in this two concept (increased landslide susceptibility and increased landslide remobilization). And relate them to the relevant literature discussion. On this you should also cite Dahlquist and West which showed the extra rainfall induced new debris flow was anomalous in 2015 only, whereas coseismic remobiliztions of landslides into debris flow was still substantial in 2016.

Response: As outlined in the previous response, lines 252 – 256 state clearly the difference between preconditioning of new landslides and increased remobilisation. Text also changed as suggested, including reference to Dahlquist and West, on lines 280 – 301.

Comment 7: L320: "less well understood" … Not sure the short term preconditioning I better understood, but it is more frequently observed. So I would rather say that.

Response: Changed, line 354.

Comment 8: L321-322 : You suggest only large EQ can mobilize deep landslides but this is not so simple when you look at data either in Nepal or elsewhere, where very deep landslides can also be mobilized during rainfall (eg Morakot or Talas typhoon in Taiwan/Japan, see Marc et al 2018).

Response: Changed to state that rainfall can exploit deep damage, lines 355 – 357.

Comment 9: L325-327 : Why do you consider all these EQ for their "preconditioning impact" and not for their coseismic impact and thus for the long-term budget of your conclusions ? Also you are contradicting yourself : you said long preconditioning may affect EQIL so you should look at Gorkha coseismic landslides (NOT the MIL) and see if they are better predicted when also considering past EQ shaking, like what they did in Parker et al., 2015.

Response: As recommended by the reviewer and editor, we have now dropped the long-term budget part of the paper. Lines 357 – 359 demonstrate how EQIL can be impacted by preconditioning. However, lines 360 – 361 make it clear that in this case we are interested in only whether preconditioning affected MIL (as this is what the rest of the analysis looks at).

Comment 10: L345 : you report R2 = 0.34 in text but R2= 0.377 in Figure S1. Also this figure should plot Excess mass wasting VS Excess Topography (NOT the opposite as it is now) and then show the R2 …

Response: Figure changed as suggested. The R2 values have thus changed, and are now the same in both the figure and text – lines 333 – 334 and line 374.

Comment 11: L419 : I am surprised by this recurrence time. In Marc et al 2019 (see details there) we concluded to a 300 yrs return for Mw 7.9. Based on Bollinger et al 2014 but also the observed Gutenberg Richter from Avouac, 2015. Anyway, see my major comments but the EQ budget need to be substantially updated or abandoned.

Response: As suggested by the reviewer and editor, the long-term budget content has been removed from the paper.

Comment 12: L465 : it can be pansharpened with pan chromatic imagery at 15m resolution (This is not a technically a resampling as it is an extra layer of different data.)

Response: Changed, line 452.

Comment 13: "All types of rainfall triggered mass wasting" Unclear and the reference is not helping. Do you mean not only landslides but also say, rilling, debris flow ? Or do you mean mapped landslides were not differentiated by types ? Or something else ?

Response: We meant, that landslides were not differentiated by types – this is now stated explicitly on lines 461 – 462.

Comment 14: L481: "fully within existing mass-wasting" is a bit confusing; Do you mean that you mapped textural changes within active landslides, for which the responsible process is likely unclear and A-V scaling unconstrained. Or do you refer to the scarring of a revegetated previously mapped landslide ? In this second case it has more chance to be a proper landslide, and a reactivation too, but I would phrase: "scarring/vegetation disturbance within the boundary of previously mapped landslides were considered reactivation." something like that. See Major comment fro the required clarification between Reactivation and Remobilization

Response: It was meant that scarring of a revegetated, previously mapped landslide, though in some cases this may have included textural changes within a landslide. This is now made clearer in the methods on lines 468 - 470, and potential issues arising from A-V scaling for any remobilisations on lines 519 – 523.

Comment 15: L499 : I am not sure this prove that the difference in mapping duration is not affecting your result, given obviously the primary control will be ASM rainfall. I appreciate your effort but if you want to support this claim I think the figure should show that there is no correlation between the non monsoonal number of months (ideally the non monsoonal amount of rainfall in case some year had extra monsoonal substantial precipitation) and the deviation from your landslide volume / Monsoon rainfall relationship.

Response: We now also correlate non-monsoonal APHRODITE rainfall amount with deviation from the APHRODITE-based normalisation in Fig. 3b, showing that there is no correlation between the two. Supp materials Fig. S10, line 484 – 491.

Comment 16: L507 : or roads ? From the rebuttal I had the impression roads related slides where mapped separately and then not included in this version, it is not reflected in this sentence.

Response: Changed to reflect that road-related slides were not included – lines 495 – 496.

Comment 17: L537 : "it is the only accessible precipitation product with a spatial resolution of at least 0.25o by 0.25o that spans our required time period of 1988 – 2018" This is simply not true. At least the two following product MSWEP (Beck et al 2017) and Aphrodite (Yatagai 2012) cover the period and have the resolution you want. I do not think you have any reason to use Persiann, which by only using infrared measurement is severely limited (see conclusions in Beck et al 2017). L559: Ok but others product are also made for that, possibly with less issues (for daily rainfall) see Yatagai 2012, Beck et al 2017.

Response: Changed to reflect that there are other products with similar time-span, see lines 534 – 536 in the methods. As outlined in the response to the first major comment (and on lines 526 – 579), we now use APHRODITE for the majority of the analysis, with PERSIANN only used to consider the post-2015 period.

Comment 18: L619-621 : You argue you can remove the Jure landslide because of progressive failure. I agree but it may not be the only large landslides that may be decoupled from the monsoon. In Marc et al 2019, several other landslides were shown to be anomalous relative to the monsoon in which they occurred, and this was for only a few years…. So clearly your analysis may have several years disturbed by exceptional landslides, and removing only Jur may not be adequate…. In the figure above I show the simplest correction that is to remove the largest scar volume in each year… In Marc et al 2019 a threshold for the ratio between the largest and second largest scar area seemed to be able to discriminate suspicious slides, and could be reused here.

Response. Following the reviewer's suggestion, we now remove all largest landslides in a given season that had scar volumes greater than 2* the second largest. See lines 156 – 160 in the main text, and lines 607 – 615 in the methods.

Comment 19: Figure 2: Why not putting all years, including Post EQ ? This should allow to show which years are within the PreEQ fit and which are not. The 1Sigma uncertainties should appear in this figure too I would say, and if possible in the fit equation.

Response: Earthquake years added to the plot, as are standard errors in the fit equation, see fig. 2.

Comment 20: Corrected and Uncorrected terms are not ideal terms are it sounds like one is true and the other false whereas they represent different things. I would favor something like: New and New + Reactivation/Remobilization.

Response: We have changed these terms to "New + RR" (where RR is reactivation/remobilised) and "New Only" throughout the manuscript, e.g. lines 102 – 104.

Comment 21: Figure 4 : I would recommend to show PDF curves rather than CDF. Because they can be better compared to previous work, especially in Marc et al 2019. Though the fact you find a power-law decay of ~2.5 is consistent with the higher resolution inventory from Marc et al 2019 so it is reassuring.

Response: We have changed to using PDF curves and the three-parameter inverse gamma fit as is comparable to the literature. See fig 4 and lines 160 – 177 in the main text, and lines 618 – 663.

Reviewer 3.

Comment 1: I found the terminology used to describe the various mass wasting volumes (Total mass wasting volume, Corrected mass wasting volume, and Scar volume) used in this paper confusing. In the methodology section (lines 513 – 514) Scar area (or volume) is described as the result of a correction of the total landslide polygons to remove the influence of long runout landslides. This leads to confusion when discussing the Corrected mass wasting volume, which is just the total mass wasting volume minus the mass wasting associated with reactivating previous deposits. I suggest renaming the "Corrected mass wasting" to a term which closer describes the process by which it is produced, perhaps "New mass wasting" or "non-reactivating mass wasting".

Response: We have now changed our terminology throughout the manuscript and figures to: "New Only" for just new mass-wasting, and "New + RR" for new and reactivated/remobilised mass-wasting. E.g. lines 102 – 104 / 273 – 278,

Comment 2: I also would like to see some uncertainty analysis undertaken on the definition of an extreme rainfall event. Currently the authors use a threshold of 1.8x the mean annual 24-hour total of precipitation of a cell to define an extreme event based upon the 2002 rainfall event. This threshold could be too high for other unrecognised events which trigger abnormal volumes of landsliding. A possible method of quantifying the uncertainty in the precipitation threshold used could be via analysing the mass wasting inventory. A threshold for an extreme mass wasting event could be defined and compared to the precipitation threshold required to produce it, any unpredicted

extreme mass wasting events can then be quantified and used as uncertainty for the precipitation threshold.

Response: As requested by reviewer 4, we have now quantified and defined extreme rainfall more carefully using Z-score anomalies. Hopefully this new analysis and treatment of extreme rainfall satisfies this comment also. See lines 201 – 249 in the main text for this new analysis.

Comment 3: Line 112: Does the correct volume only apply to mass wasting produced by mass wasting?

Response: It is not entirely clear what the reviewer means by this comment – however, this section has now been re-written to clarify the meaning of preconditioning and how this relates to our two measures of volume (lines 101 – 112) so hopefully this has answered the reviewers query.

Comment 4: Line 123: What are the units of the Monsoon Strength?

Response. Units are in mm/grid (i.e. the total precipitation across all grid cells divided by the number of grids to get a grid-average across the study region). This is now stated explicitly on lines 125 – 127.

Comment 5: Line 136: Which mass wasting volume is used to define the ASM strength normalised mass wasting?

Response: All measures of mass-wasting volume were used – this has now been re-phrased for clarity on lines 133 – 140.

Comment 6: Line 175: How well do the locations of the rainfall event and the 2002 mass wasting correlate? (reference figure 5).

Response: Figure 6 (which was figure 5), now shows the cells impacted by the 2002 rainfall plus the 2002 mass-wasting events, showing the clear spatial coincidence of the two. That the 2002 rainfall event was coincident with the 2002 mass-wasting perturbation is stated on lines 187 – 189.

Comment 7: Line 181: Make it clearer that you believe the extra mass wasting is due to case 2 (170) i.e. A localised rainstorm not recorded in the ASM strength

Response: Sentence has been re-written to make this clearer, see lines 194 – 196.

Comments 8/9: Line 197: It is worth highlighting the 1993 event is not recorded in the RX1 data, so the precipitation threshold is based exclusively on 2002. Line 203: How are the cells related to the rainfall event identified?

Response: As stated previously, we now use a new method to assess extreme rainfall (based on a longer times series that now included 1993) and make it clearer how extreme cells are identified

(based on the Z-score associated with the mean/standard deviation of a cell across a 64-year time series). See lines 201 – 249.

Comments 10/11: Line 229: Perhaps fits would be a better way to describe the equations. Line 235: Across the entire mapped region and period

Response, this section has now been dropped from the manuscript.

Comment 12: Line 341: It is not clear why ridges are discussed and then the concept of excess topography is brought up.

Response: For clarity, we have removed the word "ridges" and replaced with "topographic excesses". See lines 323 – 324.

Comment 13: Line 342: When are these changes relatively to? Between two inventories?

Response. This section has been re-written for clarity. The change in 2015 monsoon-triggered mass-wasting (or "excess") is based on the increase in mass-wasting observed in the 2015-monsoons season in a given grid cell relative to that grid-cells mean for all unperturbed years. See lines 665 - 677 in the methods section.

Comment 14: Line 372: The 1988 earthquake had a maximum PGA of 0.28g

Response: This section has been largely re-written – lines 346 - 347 now state the maximum PGA for 1988 as 0.28 g.

Comment 15: Line 443: You could mention your finding of the requirement of a recovery time of an area after an extreme mass waste event as this is the opposite of the earthquake preconditioning. i.e. An event reduces future mass wasting

Response: Added reference to landscape recovery, see lines 403 – 405.

Comment 16: Line 456: How were landslides mapped in 2013? Were new landslides assumed to occur in 2013 or discounted?

Response: Landslides in 2013 were mapped as normal, i.e. with Landsat 8 imagery from before the 2013 monsoon season and Landsat 8 imagery from after the 2013 monsoon season. This is now stated explicitly on lines 442 – 444.

Comment 17: Line 537: It would be useful to have a quick definition of how reactivated mass wasting was identified within the methods.

Response: This is now included in the methods section: see lines 468 – 470.

Comments 18/19: Line 584: What are the units for the x-min? Line 592: Quote the X-min of Marc et al and Roback et al.

Response: We have changed our approach slightly to assessing landslide size, now using PDF's and the three-parameter inverse gamma power law distributions (see methods section, lines 617 – 663). We have now given the units of the rollover (m2) (see main text, lines 169 – 177), and have added the threshold areas of both Roback et al., and Marc et al., see line 656.

Comment 20: Line 632: A complete list of the perturbed years rather than a hyphen would be clearer (2000, 2001, 2002, 2003, 2004)

Response: This section has now been dropped from the manuscript.

Comment 21: Figure 1: Does this figure include the Coseismic mass wasting?

Response: No. Have added "monsoon-triggered" to the figure 1 caption to clarify this, line 708.

Comment 22: Figure 3: The left Y axis (ASM Normalised Mass wasting rate) could start at 0 and the Earthquake Magnitudes could be displayed on the left for clarity

Response: Have shifted the axis and moved the EQ magnitudes to the left as suggested (see Fig 3a – b).

Comment 23: Figure 4: The symbols in the legend should be larger to see the colours easier

Response: This figure has now been dropped and replaced with figs showing PDFs and fitted inverse gamma distributions – see Fig 4.

REVIEWER COMMENTS

Reviewer #3 (Remarks to the Author):

Review of 30-year record of Himalaya mass-wasting reveals landscape perturbations by extreme events

Oliver Francis

My area of expertise is the remote sensing of landsliding from satellite imagery and the impact of earthquakes on landsliding and erosion rates. Therefore, I feel I have the expertise to review this paper.

The manuscript by Jones et al. attempts to identify a relationship between the strength of annual monsoon rainfall and mass wasting volume in the Himalaya and determine factors which can perturb this relationship. Their analysis has revealed that intense localised rainstorms can produce above average mass wasting for a given monsoon strength. These events should be considered separately from the overall trend of mass wasting and monsoon strength to ensure the best fit. These events could reduce the mass wasting of the next large event as the landscape needs to recharge before its slopes can fail en masse again. By contrast earthquakes can precondition areas to fail at higher rates than expected, though this effect seems to be short lived. Areas of high excess topography showed the greatest amount of preconditioning following the 2015 Gorkha Earthquake, however it is unclear whether this pattern holds for other earthquakes.

In my review of a previous version of this manuscript I raised some minor concerns concerning the use of some terminology and how extreme rainfall events were determined in the study. I believe my previous comments have largely been addressed in this version of the manuscript.

I believe this is good well written manuscript and I found it to be interesting and thought provoking. I believe it is worthy of publication in Nature Communications. However, I have a few comments which I feel need to be addressed before it is ready for publication. I have summarised my thoughts on the manuscript below with specific line numbers highlighted where appropriate.

General comments

The idea that a landscape recovery time exists for the recurrence of landslides in a particular area is very interesting but feels a little underexplored here. The authors suggest that the ability of a hillslope to respond to a perturbation depends on the history of the area, in this case the time since the last major storm. If their hypothesis is correct you would expect to see a decrease in the mass wasting of the local area for several years regardless of whether another large storm occurs or not. In other words, is there a reduction in the mass wasting volume in the year 2003 or 1994 (or even in 2005 in the area which was affected by the extreme rainfall) in the local area of the previous extreme rainfall events? If there is a reduction this would further support the proposed hypothesis.

It is not completely clear why the authors chose to focus on excess topography as a measure of landslide susceptibility to combine with PGA, particularly as it seems to work as an indicator for only one of the earthquakes studied. Previous studies (Meunier et al. 2008; Marc et al. 2016) have indicated that topographic slope of an area is a reasonable approximation of the susceptibility of slope to coseismic landsliding and therefore seismic damage of an area. A comparison between the impact of excess topography and slope on the prediction of postseismic landslide preconditioning would support the author’s choice of metric.

I feel the manuscript could use some additional figures to help the reader better understand the relationships between PGA, excess topography and mass wasting volumes. I recommend the authors

consider producing a map showing the epicentres of the earthquakes being analysed along with the excess topography and PGA of the earthquakes highlighted. Currently the reader has to solely rely on the author's description of the area which does not feel sufficient as the author proposes several reasons for the null results of several analyses. A map or another visual representation of the area will enable the reader to decide for themselves the likely reasons behind the presented results.

Minor comments

Line 68: Authors state the Gorkha earthquake caused a post seismic perturbation of mass wasting before casting doubt on the idea in line 74.

Line 121 onwards: It is not clear what total precipitation > 25mm means. Is this the sum of the precipitation of days which had total rainfall values above 25mm?

Line 195: Authors could be clearer about whether landslides were reported to be associated with these rainfall events.

Line 211: A simple definition of Z score either here on in the methods would ensure the reader understands the use of this metric.

Line 223: Dismisses the role of rainfall in the 1989 perturbation but in line 355 the authors change their minds to suggest rainfall may have played a part.

Line 226: This section is a little imprecise in the description of the gridded Z scores. Line 226 states that the 2004 monsoon has 11 days of Z scores above 12 but does not state whether this is for particular cells or the entire region. Similarly line 231 states the extreme rainfall occurred after the 15th of June but it is not clear if the authors are discussing all 8 of the days. Line 253 discusses the return period of Z scores but it is not clear whether the return periods relate to the entire area or particular grid cells.

Line 237: The comparison between 2 groups of cells at a single time does not seem strong enough to determine whether the previous 2002 event had a long-lasting effect on the area. Instead, the authors could use their % change based analysis used in the 'Impact of earthquakes' section to compare the cells. This would remove any stochastic differences which maybe present when comparing the groups for a single time slice.

Line 278: Do these remobilisations include remobilisation of coseismic landslides?

Line 319: Are the grid cells used in this analysis the same as the APHRODITE cells used before?

Line 371: This section of analysis would be greatly improved with a map view of the area. Currently the readers have little information of where the epicentres of the earthquakes are in relation to each other until the end of the paragraph.

Line 419: This section does not discuss the other result of the manuscript, that large events can also reduce the likelihood of another extreme mass wasting event occurring.

Line 451: Are landslides from 2011 – 2012 mapped in the pre-monsoon satellite image of 2013 and thus discounted from the inventory?

Line 478: What is the difference between a remobilisation and a reactivation? In the rest of the manuscript these terms seem to be used fairly interchangeably

Line 509: When coseismic landslides are identified and removed are the remobilisations within them

still considered for the next time slice?

Line 581: Words missing or out of order within the brackets at the end of the line.

Line 586: This last section is a little unclear. From what I understand the main product used is PERSIANN-CDR as this covers the entire period. The APHRODITE product is used to validate the PERSIANN-CDR analysis and determine the Z scores of the daily precipitation. This last section suggests that the PERSIANN-CDR product is only used for the post 2015 rainfall events.

Figure 4: If the authors decide to put a map of excess topography or earthquake PGA in the main text, figure 4 seems like a good candidate to be put into the supplemental to make room if the number of figures becomes an issue.

References used in this report:

Marc, O. et al. 2016. A seismologically consistent expression for the total area and volume of earthquake-triggered landsliding. Journal of Geophysical Research: Earth Surface 121(4), pp. 640–663. Available at: http://doi.wiley.com/10.1002/2015JF003732.
Meunier, P. et al. 2008. Topographic site effects and the location of earthquake induced landslides. Earth and Planetary Science Letters 275(3–4), pp. 221–232. Available at: http://dx.doi.org/10.1016/j.epsl.2008.07.020.

Reviewer #4 (Remarks to the Author):

On this second reading, I think that the work by Jones et al., has gained in focus and robustness. So I congratulate the authors for the efforts they put in addressing all my comments and performing substantial amount of new analysis. The monsoon relationship is I believe more clearly exposed and demonstrated, as well as the importance of extreme rainfall triggering. The role of excess topography seems quite supported by the data.
I think overall the dataset and analysis of Jones et al., will be well received by the geomorphological and natural hazard communities and should have broad impact.

I think the work is nearly ready for publication, and only have a number of minor comments for a few places where some clarifications or nuances could be added.
My ultimate main concern is about the extreme rainfall analysis that gives somewhat inconsistent results, probably because of its naive approach (zeta score). I would recommend that the authors acknowledge a bit more the limit of their current approach and dataset or else back it up with some modest refinement of their analysis, and finally update the abstract and conclusions on this question (see my suggestion below).

Below are Line by line minor comments.
Congratulation again to the authors for this hard work and interesting results.

Odin Marc

Line By Line Comment (Number refer to marked change manuscript) :

L244 :the extremity ? Can you say that ? What about writing :
it is necessary to define how extreme the 1993 and 2002 cloud outburst events were ? (the I realize it looks quite a bit like a previous sentence a few line above) .

L262 : “Correlate” is a bit vague and suggest you “correlated” two dataset which I think you did not...

coincide may be a better term here.

l267 : Nb of days above the Z score anomaly Interesting, but beware that 3 consecutive days with high zeta score or 3 distributed days with high zeta score is very different the former being rarer (longer recurrence time) and likely more efficient at triggering landsliding … So in case you see that in some years, sequences of anomalies you should mention it, and re assess the return period of such events (by doing Z score of for the total rainfall over X days (X depending on the sequence they find..) and not daily …)

L268-273 : The structure is a bit heavy given you are gone abandon one option
What about saying : Given the 2004 extreme anomalies occurred on XX(give dates) it is unlikely that the lack of landslide response could be attributed to an incomcpletely saturated hillslopes (ref, ref)

L276 : What about the Gruber et al Permafrost map?? No mention of how it could explain the lack of 2004 sliding ... From the map it looks limited to the northern areas with the highest peak , where anyway mapping must have been limited by the lack of vegetation… Clarify or remove.

L280-282 : Careful here, you do not state it but this mechanism (a sort of negative preconditioning) is typical of soil landslide. So do you think your mapping (and especially volume estimate) is mainly driven by slides that depends on the presence of soil, or more bedrock landslides ? On similar data I usually found the large landslides (presumably bedrock) where

Further it seems to me that this mechanism is directly contradicted by the observations of post seismic landsliding: In the framework where landsliding is limited by the available soil to fail, the coseismic massive amount of failure should exhaust the slopes which we would then expect to have a lesser response to future rainfaill You (and I and others in previous works) have shown the opposite, with more landsliding immediately after the EQ, and then no less in the following years ...

So, to me this explanation seems quite shaky, and inaccuracy or mislocation of the extreme rainfall seems more likely, and should probably be mentionned as an alternative (and if possible evaluated)… Especially you cite a report for 500 mm in a few days over a given area in 1993, how does that compare to Aphrodite 93 event ?

L283-285 : I am skeptical about this statement and also have to say it is not very clear to what recovery timescales the author refer ...10- 100 years ?

L287 : Again I would put coincide instead of Correlate.
Also more fundamentally, you need to be careful because the rainfall Zeta score are quite poorly consistent with the magnitude of the extra landsliding : 93 is the strongest deviation but one of the weakest rainfall anomaly, while 2004 had no landslide anomaly but a strong rainfall anomaly … TO me the easiest explanations (that are not really mentioned for now) are 1) inaccurate/mislocated rainfall measurement or 2) inadequate quantification of the extreme . For 1) you can check if strong anomaly are just outside of your zone … For 2) you can do the Zscore analysis on 2, 3 or 5 days bins that may be more relevant time scale for widespread landsliding (different year may have been triggered by different timescales of intense rainfall)… If it works such index could enhance FIG, if it does not you can just mention that these longer timescales do not help to explain landsliding ...

L293-294 : As of now return time are poorly convincing … The 93 landsliding seems to have been a 1/40 yr event, but with the current methods of the author it is a 4yr return rainfall … Reversely for 2004 ... Maybe accounting for temporal cluster of the extreme daily Zscore is the key to this mismatch … If not the authors should at least highlight this inconsistency, and maybe state that "landslide anomalies have 20 yr return (2 in 40 yrs) and relevant rainfall return may span between 5 and 30 yrs ".

General comment/perspective on Paragraph about ERIL :
Overall, reading this new addition I wondered why the authors did not apply the same approach for ERIL and EQ preconditioning : They have grids with the average monsoon landsliding from all unexceptional years, they can compute the % change or ratio of the landsliding during the extreme rainfall year and correlate it to the rainfall Z score (no need of threshold) or Z score * ET ??
I guess it is ok if the author consider this as too much, but it seems to me that they already have all the cards in hand and could (potentially) transform their qualitative map into a plot similar to Fig 7 but with an index relative extreme rainfall (Landslide anomaly vs Rainfall anomaly in brief !) it would be much more striking and exciting …

L301 : Ref 3 is ok here but Marc et al 2015 is more relevant and should be added here.

L363-365 : “It is generally accepted” : This is maybe exxagerated, considering other works discussing definition and causes of post-seismic landslinding (eg Kincey et al 2020 https://agupubs.onlinelibrary.wiley.com/doi/10.1029/2020JF005803)
Maybe rephrase a bit to something like : “It has been proposed …”
Also you may want to cite here the recent comparison of geophysical data and landslide data (including Gorkha) that brings some extra constraints and discussion to this issue of pos seismic short term damage and preconditioning : Marc, O., Sens-Schönfelder, C., Illien, L., Meunier, P., Hobiger, M., Sawazaki, K., Rault, C., and Hovius, N.: Toward Using Seismic Interferometry to Quantify Landscape Mechanical Variations after Earthquakes, Bulletin of the Seismological Society of America, https://doi.org/10.1785/0120200264, n.d.

L376 : The main aftershock must be shown on Map 1 !!

L445 : I am pretty sure that with your amount of data an increase from R2=0.71 to R2= 0.72 is not significant . Doing a bootstrap resampling of yout XX gridpoints 10,000 times would give you and estimate of the R2 variability and I doubt it will be less than 0.1 ...
The authors do acknowledge later that 2011 may not have any impact. I think given their result in Fig 7 the authors could go straight to the point and say there is no significative improvement when considering any of the past EQ shaking patterns.

L479 : Hmm this result may need to be a nuanced or removed from the conclusions as it is not very convincingly demonstrated… (see my comments above).

L673 : The key study aim

L748 and L 864 : Landsat Software ?? >> LAMPRE software maybe (given the Ref 78).

L767 : You say minimum feature mapped but then give values of 2500 and 1200m2 which are quite large (and sound more like roll over values) Please check and correct.

L870-880 : The caption suggest you summed PGA from various but the label suggest you substract them please clarify the caption or label.

Also here if you “sum” previous EQ PGA it means you ignore the fact they could have caused coseismic

landslide which would have exhausted suitable site for soil landsliding … No problem for me but it relates to my previous comment about you some times arguing for exhaustion of potential landslide sites (for 2002/2004 anomalies).

Fig 1 map : The locations of the relevant EQ is missing, main aftershock, 1989, 1934, 2011 etc The distant ones can be in the inset if needed….

Fig 2 : Double check the labels for outliers, in g there are two dots labeled 2015

Except for that Fig 2, 3 and 4 are good.

Fig 5 is a bit a lonely figure and you may consider merging with Fig 3B ? you would just need to add a Y label … although maybe instead of merging it you may add other proxy for extreme rainfall (like Zeta score of Triad or Pentad instead of daily time series ..) See previous comment.

Fig 6 : Why do you show 2002 and 2004 but not 1993 landsliding (which was a more widespread anomaly) ? And what about the 1993 rainfall anomalies ??
Also looking at my previous review it seemed quite clear that a rainfall anomaly was present in 1993 in the Aphrodite data, is it outside of your study zone ?
If yes it may still be worth to mention/show it in a supplementary figure, as the exact extent of the area affected by intense daily rainfall may be quite inaccurate in the rugged Himalayan terrain Given you have report about intense floods and damages you know for sure there was extreme rainfall in the area.

Also check if Permafrost is useful or not… Now it is unclear what it brings.

Fig 7 : Labels are confusing as of now: I think (PGA2015 PGA2011)*ET45 would be much less ambiguous .

Acknowledgment : Please write Odin Marc (not Marc Odin that suggests my first name is Marc and family name Odin ...)

References : 50 and 67 are the same (Marc et al 2018). Error ? It seems 50 should be the only one.

Responses to Reviewers

We would like to thank both reviewers for their constructive and useful comments. The below paragraphs detail our responses and changes based on their suggestions. Furthermore, the tracked changed document includes comments to explain the key changes we have made on the manuscript itself. These comments use R3 and R4 to refer to each reviewer. The line numbers referenced in the below responses correspond to the line numbers in the tracked-changes document, though a "clean" non-tracked change version of the manuscript is also provided.

Reviewer #3 (Remarks to the Author):

Review of 30-year record of Himalaya mass-wasting reveals landscape perturbations by extreme events
Oliver Francis

General comments

General Comment 1: The idea that a landscape recovery time exists for the recurrence of landslides in a particular area is very interesting but feels a little underexplored here. The authors suggest that the ability of a hillslope to respond to a perturbation depends on the history of the area, in this case the time since the last major storm. If their hypothesis is correct you would expect to see a decrease in the mass wasting of the local area for several years regardless of whether another large storm occurs or not. In other words, is there a reduction in the mass wasting volume in the year 2003 or 1994 (or even in 2005 in the area which was affected by the extreme rainfall) in the local area of the previous extreme rainfall events? If there is a reduction this would further support the proposed hypothesis.

General Response 1: Based on the comments by Reviewer 4, and after further exploring the Z-score / mass-wasting change data, we now conclude that the idea of landscape recovery in this case is less likely than other explanations (see lines 252 - 273). As such, we largely drop this idea from the manuscript and so have not assessed it further by looking at 2003 or 1994 as suggested here.

General Comment 2: It is not completely clear why the authors chose to focus on excess topography as a measure of landslide susceptibility to combine with PGA, particularly as it seems to work as an indicator for only one of the earthquakes studied. Previous studies (Meunier et al. 2008; Marc et al. 2016) have indicated that topographic slope of an area is a reasonable approximation of the susceptibility of slope to coseismic landsliding and therefore seismic damage of an area. A comparison between the impact

of excess topography and slope on the prediction of postseismic landslide preconditioning would support the author's choice of metric.

General Response 2: We had actually previously considered slope as well as excess topography, but had found that it did not improve the relationship between PGA and mass-wasting. However, to ensure the reader knows this null-result, we have now added several sentences on lines 427 - 432 outlining this, as well as a new supplementary figure (Fig S9) showing that slope*PGA does not show a significant relationship with excess mass-wasting in 2015.

General Comment 3: I feel the manuscript could use some additional figures to help the reader better understand the relationships between PGA, excess topography and mass wasting volumes. I recommend the authors consider producing a map showing the epicentres of the earthquakes being analysed along with the excess topography and PGA of the earthquakes highlighted. Currently the reader has to solely rely on the author's description of the area which does not feel sufficient as the author proposes several reasons for the null results of several analyses. A map or another visual representation of the area will enable the reader to decide for themselves the likely reasons behind the presented results.

General Response 3: We have now added a new figure (Figure 7), as requested, showing the earthquake epicentres and PGAs. We decided not to show excess topography on this map as it made the figure very busy and essentially unreadable, even if PGA was just shown as contours.

Minor comments

Comment 1: Line 68: Authors state the Gorkha earthquake caused a post seismic perturbation of mass wasting before casting doubt on the idea in line 74.

Response 1: clarified that we know it caused a post-seismic perturbation in 2015 (line 71), but that for 2016 onwards it is challenging to distinguish (line 76).

Comment 2: Line 121 onwards: It is not clear what total precipitation > 25mm means. Is this the sum of the precipitation of days which had total rainfall values above 25mm?

Response 2: added brackets to clarify that it is the sum of precipitation days above 25 mm, lines 125 – 127.

Comment 3: Line 195: Authors could be clearer about whether landslides were reported to be associated with these rainfall events.

Response 3: We have now added the reported landslide information, lines 201 – 203.

Comment 4: Line 211: A simple definition of Z score either here on in the methods would ensure the reader understands the use of this metric.

Response 4: definition now added on lines 218 – 219.

Comment 5: Line 223: Dismisses the role of rainfall in the 1989 perturbation but in line 355 the authors change their minds to suggest rainfall may have played a part.

Response 5: Slightly re-phrased for clarity. On lines 233 - 234 now say that extreme rainfall alone cannot explain these perturbations. And on lines 445 - 448, add a sentence saying that neither the earthquake nor rainfall alone can explain the 1989 perturbation, suggesting that it is potentially due to a combination of the two.

Comment 6: Line 226: This section is a little imprecise in the description of the gridded Z scores. Line 226 states that the 2004 monsoon has 11 days of Z scores above 12 but does not state whether this is for particular cells or the entire region. Similarly line 231 states the extreme rainfall occurred after the 15th of June but it is not clear if the authors are discussing all 8 of the days. Line 253 discusses the return period of Z scores but it is not clear whether the return periods relate to the entire area or particular grid cells.

Response 6: clarified that the days with Z > 12 were across the entire region (line 237), that all 8 days occurred after June 15th (lines 239 - 240) and that the return periods relate to the entire study region (line 293).

Comment 7: Line 237: The comparison between 2 groups of cells at a single time does not seem strong enough to determine whether the previous 2002 event had a long-lasting effect on the area. Instead, the authors could use their % change based analysis used in the 'Impact of earthquakes' section to compare the cells. This would remove any stochastic differences which maybe present when comparing the groups for a single time slice.

Response 7: Lines 252 - 273 now more robustly consider this idea of landscape recovery, including the use of the % change based analysis as suggested.

Comment 8: Line 278: Do these remobilisations include remobilisation of coseismic landslides?

Response 8: clarified to say that yes, this comprises remobilisations of coseismic landslides, line 103.

Comment 9: Line 319: Are the grid cells used in this analysis the same as the APHRODITE cells used before?

Response 9: On line 401 now clarify that yes, they are the same grid cells.

Comment 10: Line 371: This section of analysis would be greatly improved with a map view of the area. Currently the readers have little information of where the epicentres of the earthquakes are in relation to each other until the end of the paragraph.

Response 10: We have now added a new figure (Figure 7) showing the epicentres and PGAs of the main earthquakes.

Comment 11: Line 419: This section does not discuss the other result of the manuscript, that large events can also reduce the likelihood of another extreme mass wasting event occurring.

Response 11: Based on the comments of the other reviewer and the other changes made within this revision, we have not added this to the conclusions as it is not a significant result.

Comment 12: Line 451: Are landslides from 2011 – 2012 mapped in the pre-monsoon satellite image of 2013 and thus discounted from the inventory?

Response 12: lines 546 - 547 added to clarify that landslides from 2011 and 2012 were fully discounted from the inventory, with only new landslide occurring post-2012 mapped.

Comment 13: Line 478: What is the difference between a remobilisation and a reactivation? In the rest of the manuscript these terms seem to be used fairly interchangeably

Response 13: lines 572 - 575 now clarify that a reactivation is defined as failures involving the displacement of previously undisturbed material that initiated from or intersected with the boundary of a previous failure. Whilst line N defines remobilisations as movements of previously disturbed material only.

Comment 14: Line 509: When coseismic landslides are identified and removed are the remobilisations within them still considered for the next time slice?

Response 14: Lines 602 – 603 now clarify that reactivations and remobilisations of coseismic mass-wasting are included. This is also clarified on lines 356 – 357.

Comment 15: Line 581: Words missing or out of order within the brackets at the end of the line.

Response 15: sentence corrected, lines 678 – 680.

Comment 16: Line 586: This last section is a little unclear. From what I understand the main product used is PERSIANN-CDR as this covers the entire period. The APHRODITE product is used to validate the PERSIANN-CDR analysis and determine the Z scores of the daily precipitation. This last section suggests that the PERSIANN-CDR product is only used for the post 2015 rainfall events.

Response 16: Line 685 has been re-phrased to clarify that PERSIANN-CDR is used to obtain a time-stable assessment of the entire time series, including the post-2015 period which APHRODITE doesn't cover, and that APHRODITE is used to corroborate the PERSIANN-CDR data and allow assessment of extreme daily rainfall.

Comment 17: Figure 4: If the authors decide to put a map of excess topography or earthquake PGA in the main text, figure 4 seems like a good candidate to be put into the supplemental to make room if the number of figures becomes an issue.

Response 17: For now we have left figure 4 in the main manuscript, but will bear this in mind if the editor decides there are too many figures.

Reviewer #4 (Remarks to the Author):

I think the work is nearly ready for publication, and only have a number of minor comments for a few places where some clarifications or nuances could be added.
My ultimate main concern is about the extreme rainfall analysis that gives somewhat inconsistent results, probably because of its naive approach (zeta score). I would recommend that the authors acknowledge a bit more the limit of their current approach and dataset or else back it up with some modest refinement of their analysis, and finally update the abstract and conclusions on this question (see my suggestion below).

Odin Marc

Line By Line Comment (Number refer to marked change manuscript):

Comment 1: L244 :the extremity ? Can you say that ? What about writing :
it is necessary to define how extreme the 1993 and 2002 cloud outburst events were ?
(the I realize it looks quite a bit like a previous sentence a few line above) .

Response 1: Phrasing changed as suggested on line 212.

Comment 2: L262 : "Correlate" is a bit vague and suggest you "correlated" two dataset which I think you did not... coincide may be a better term here.

Response 2: Changed to coincide as suggested, line 232.

Comment 3: l267 : Nb of days above the Z score anomaly Interesting, but beware that 3 consecutive days with high zeta score or 3 distributed days with high zeta score is very different the former being rarer (longer recurrence time) and likely more efficient at triggering landsliding ... So in case you see that in some years, sequences of anomalies you should mention it, and re assess the return period of such events (by doing Z score of for the total rainfall over X days (X depending on the sequence they find..) and not daily ...)

Response 3: We have now added a new paragraph that considers consecutive vs distributed high rainfall days on lines 244 – 250. In summary, this paragraph highlights that most of the other perturbations did not coincide with consecutive

high rainfall days, indeed, only 2002 experienced just two consecutive high rainfall days. As such, the lack of high rainfall consecutive days cannot explain why 2004 doesn't observe a perturbation, and as we don't see consecutive high rainfall days, we do not re-asses the return periods. However, on lines 306 - 308 we highlight that considering consecutive vs distributed rainfall should be considered an important area of future work.

Comment 4: L268-273 : The structure is a bit heavy given you are gone abandon one option
What about saying : Given the 2004 extreme anomalies occurred on XX(give dates) it is unlikely that the lack of landslide response could be attributed to an incompletely saturated hillslopes (ref, ref)

Response 4: The structure of this section has been changed as suggested on lines 239 – 242.

Comment 5: L276 : What about the Gruber et al Permafrost map?? No mention of how it could explain the lack of 2004 sliding … From the map it looks limited to the northern areas with the highest peak , where anyway mapping must have been limited by the lack of vegetation… Clarify or remove.

Response 5: We have decided to remove the Gruber permafrost data as it does not contribute to any of the results / discussion points.

Comment 6: L280-282 : Careful here, you do not state it but this mechanism (a sort of negative preconditioning) is typical of soil landslide. So do you think your mapping (and especially volume estimate) is mainly driven by slides that depends on the presence of soil, or more bedrock landslides ? On similar data I usually found the large landslides (presumably bedrock) where

Further it seems to me that this mechanism is directly contradicted by the observations of post seismic landsliding: In the framework where landsliding is limited by the available soil to fail, the coseismic massive amount of failure should exhaust the slopes which we would then expect to have a lesser response to future rainfaill You (and I and others in previous works) have shown the opposite, with more landsliding immediately after the EQ, and then no less in the following years …

So, to me this explanation seems quite shaky, and inaccuracy or mislocation of the extreme rainfall seems more likely, and should probably be mentionned as an alternative (and if possible evaluated)…

Especially you cite a report for 500 mm in a few days over a given area in 1993, how does that compare to Aphrodite 93 event ?

Response 6: We have now fully re-written this section, lines 244 - 308 to highlight the points raised by the reviewer, in particular to make the point that the idea of landscape recovery (negative preconditioning) is contradicted by other observations and to discuss issues relating to inaccuracy and missallocation of rainfall. Furthermore, on lines 300 - 303 we compare the 1993 Aphrodite rainfall total to that reported in the literature to illustrate that there are inaccuracies in the extreme rainfall data that could explain why not all perturbations fully correlate with extreme rainfall.

Comment 7: L283-285 : I am skeptical about this statement and also have to say it is not very clear to what recovery timescales the author refer ...10- 100 years ?

Response 7: This line has now been removed.

Comment 8: L287 : Again I would put coincide instead of Correlate.
Also more fundamentally, you need to be careful because the rainfall Zeta score are quite poorly consistent with the magnitude of the extra landsliding : 93 is the strongest deviation but one of the weakest rainfall anomaly, while 2004 had no landslide anomaly but a strong rainfall anomaly ... TO me the easiest explanations (that are not really mentioned for now) are 1) inaccurate/mislocated rainfall measurement or 2) inadequate quantification of the extreme . For 1) you can check if strong anomaly are just outside of your zone ... For 2) you can do the Z-score analysis on 2, 3 or 5 days bins that may be more relevant time scale for widespread landsliding (different year may have been triggered by different timescales of intense rainfall)... If it works such index could enhance FIG, if it does not you can just mention that these longer timescales do not help to explain landsliding ...

Response 8: Correlate changed to coincide. Furthermore, lines 244 – 250, 275 -283 and 285 - 308 have been added to mention some of the issues mentioned here including misallocation of rainfall and the potential of using consecutive days to more robustly quantify the extreme. Again, it should be noted that with the exception of 1 grid cell in 2002, we did not observe any consecutive days with high Z-scores for any of the perturbations so do not further consider this issue here, though we do mention it as a potentially interesting area of future study (lines 306 - 308).

Comment 9: L293-294 : As of now return time are poorly convincing ... The 93 landsliding

seems to have been a 1/40 yr event, but with the current methods of the author it is a 4yr return rainfall … Reversely for 2004 … Maybe accounting for temporal cluster of the extreme daily Zscore is the key to this mismatch … If not the authors should at least highlight this inconsistency, and maybe state that "landslide anomalies have 20 yr return (2 in 40 yrs) and relevant rainfall return may span between 5 and 30 yrs ".

Response 9: Lines 289 - 294 have been re-written as suggested to say that landslide anomalies have return periods of ~15 years (2 in 30 years) and that the return periods of rainfall events capable of causing these anomalies are 5 – 30 years.

Comment 10: General comment/perspective on Paragraph about ERIL :
Overall, reading this new addition I wondered why the authors did not apply the same approach for ERIL and EQ preconditioning : They have grids with the average monsoon landsliding from all unexceptional years, they can compute the % change or ratio of the landsliding during the extreme rainfall year and correlate it to the rainfall Z score (no need of threshold) or Z score * ET ??
I guess it is ok if the author consider this as too much, but it seems to me that they already have all the cards in hand and could (potentially) transform their qualitative map into a plot similar to Fig 7 but with an index relative extreme rainfall (Landslide anomaly vs Rainfall anomaly in brief !) it would be much more striking and exciting …

Response 10: We thank the reviewer for this idea, and whilst we consider this extra work to be beyond the scope of this paper, it is undoubtedly something we will look into for future publications.

Comment 11: L301 : Ref 3 is ok here but Marc et al 2015 is more relevant and should be added here.

Response 11: Reference added, line 341.

Comment 12: L363-365 : "It is generally accepted" : This is maybe exxagerated, considering other works discussing definition and causes of post-seismic landslinding (eg Kincey et al
2020 https://agupubs.onlinelibrary.wiley.com/doi/10.1029/2020JF005803)
Maybe rephrase a bit to something like : "It has been proposed …"
Also you may want to cite here the recent comparison of geophysical data and landslide data (including Gorkha) that brings some extra constraints and discussion to this issue of pos seismic short term damage and preconditioning : Marc, O., Sens-Schönfelder, C., Illien, L., Meunier, P., Hobiger, M., Sawazaki, K., Rault, C., and Hovius, N.: Toward Using Seismic Interferometry to Quantify Landscape Mechanical Variations after Earthquakes,

Bulletin of the Seismological Society of America, https://doi.org/10.1785/0120200264, n.d.

Response 12: Changed to "it has been proposed" on line 390, and have added the suggested reference on lines 392 – 394.

Comment 13: L376 : The main aftershock must be shown on Map 1 !!

Response 13: We have now included a new figure (Figure 7) that shows the location of the main aftershock.

Comment 14: L445 : I am pretty sure that with your amount of data an increase from R2=0.71 to R2= 0.72 is not significant . Doing a bootstrap resampling of yout XX gridpoints 10,000 times would give you and estimate of the R2 variability and I doubt it will be less than 0.1 ...
The authors do acknowledge later that 2011 may not have any impact. I think given their result in Fig 7 the authors could go straight to the point and say there is no significative improvement when considering any of the past EQ shaking patterns.

Response 14: Lines 467 - 472 changed as suggested to say that the R2 change is not significant and that there is no evidence that past earthquake shaking patterns had any impact on the distribution of elevated monsoon-triggered mass-wasting in 2015.

Comment 15: L479 : Hmm this result may need to be a nuanced or removed from the conclusions as it is not very convincingly demonstrated... (see my comments above).

Response 15: Sentence now removed as suggested, lines 503 – 504.

Comment 16: L673 : The key study aim

Response 16: Corrected, line 679 – 680.

Comment 17: L748 and L 864 : Landsat Software ?? >> LAMPRE software maybe (given the Ref 78).

Response 17: Corrected to LAMPRE, line 746.

Comment 18: L767 : You say minimum feature mapped but then give values of 2500 and 1200m2 which are quite large (and sound more like roll over values) Please check and correct.

Response 18: Section has been corrected, lines 762 – 766.

Comment 19: L870-880 : The caption suggest you summed PGA from various but the label suggest you substract them please clarify the caption or label.

Also here if you "sum" previous EQ PGA it means you ignore the fact they could have caused coseismic landslide which would have exhausted suitable site for soil landsliding … No problem for me but it relates to my previous comment about you some times arguing for exhaustion of potential landslide sites (for 2002/2004 anomalies).

Response 19: Labels have been clarified so it no longer looks like a subtraction.

Comment 20: Fig 1 map : The locations of the relevant EQ is missing, main aftershock, 1989, 1934, 2011 etc The distant ones can be in the inset if needed….

Response 20: As mentioned above, based on the comments from the other reviewer we have added a new figure (Figure 7) showing all of the EQ epicentres as well as some of the EQ PGAs.

Comment 21: Fig 2 : Double check the labels for outliers, in g there are two dots labeled 2015. Except for that Fig 2, 3 and 4 are good.

Response 21. Outliers have been checked, and the erroneous 2015 label removed.

Comment 22: Fig 5 is a bit a lonely figure and you may consider merging with Fig 3B ? you would just need to add a Y label … although maybe instead of merging it you may add other proxy for extreme rainfall (like Zeta score of Triad or Pentad instead of daily time series ..) See previous comment.

Response 22: We found that merging the figures made them quite messy and hard to read. Further, as outlined above, we did not look at Zeta scores of Triad or Pentads as none of the main anomalies involved consecutive high rainfall days. As such, we leave this figure as it is.

Comment 23: Fig 6 : Why do you show 2002 and 2004 but not 1993 landsliding (which

was a more widespread anomaly) ? And what about the 1993 rainfall anomalies ??
Also looking at my previous review it seemed quite clear that a rainfall anomaly was present in 1993 in the Aphrodite data, is it outside of your study zone ?
If yes it may still be worth to mention/show it in a supplementary figure, as the exact extent of the area affected by intense daily rainfall may be quite inaccurate in the rugged Himalayan terrain Given you have report about intense floods and damages you know for sure there was extreme rainfall in the area. Also check if Permafrost is useful or not… Now it is unclear what it brings.

Response 23: We show 2002 and 2004 to highlights the partial overlap between those two events. We now add the 1993 landslide mass-wasting data and Z-score anomalies to a new supplementary figure as suggested (Fig. S4), with reference to this on line 216. Permafrost data now removed from figure 6, as is the in-text reference to it.

Comment 24: Fig 7 : Labels are confusing as of now: I think (PGA2015 PGA2011)*ET45 would be much less ambiguous .

Response 24: Labels changed as suggested.

Comment 25: Acknowledgment : Please write Odin Marc (not Marc Odin that suggests my first name is Marc and family name Odin …)

Response 25: Corrected, line 801.

Comment 26: References : 50 and 67 are the same (Marc et al 2018). Error ? It seems 50 should be the only one.

Response 26: Corrected.

REVIEWERS' COMMENTS

Reviewer #4 (Remarks to the Author):

I have read the revised manuscript of Jones et al., and find it has answered well the previous review comments.
I think the study should be accepted now, and after all the various revisions think it is full of fascinating results that will be of high interest for the community.

I congratulate the authors once again, and I am looking forward to see the paper published.

I just include below a few corrections about some unclear or not so accurate wording. The author could just correct them before proceeding to final publication steps.

Best regards
Odin Marc

Last line by line comments (referring to tracked changes manuscript):

L219 : “removed” feels awkward to me in this sentenec though I see what you mean.
What about : “how many standard deviations separate a given observation from the mean of the population (containing this observation) / (it is drawn of).”

L250: I think there is no need to try harder in the context of this paper, but in case the authors plan future work on this topics, I do not think that looking at successive daily anomalies above a threshold (derived from daily anomalies) is a statistically correct way. What I was suggesting is to compute anomalies at different scale, and it would give a different results. Because if you have 3 successive daily anomalies of 8-12-9 you cannot know what anomaly it would represent in triad Z score : because the mean and standard deviation for triplets of days would be different. And because the probability of successive, localized intense rainfall events is low, the final results could change quite a bit. Feel free to contact me if you want to discuss and work along these lines in the future.

L306 : “Does not consider anomalous rainfall over shorter or longer scales”
and L308: “ across multiple days of few hours using rainfall products with higher temporal resolution ”
I think this helps to stay general as clearly some landslide clusters could be triggered by <24h intense events which would be distorted (tune down most likely) in daily records.

Figure 6: the hashed lines seems wrongly labelled ? Isn’t it rather the extent of extreme rainfall of 2002 ? given 2002 mass wasting is already in pink

Figure 8, S5,S6,S9 : In all figures with PGA / ET could the authors specify in label or caption the units (g for PGA, but what for ET for example ? Is it a surface ? A volume ?

L427-432 : Related to that it is quite clear that the difference in R2 between PGA*ET vs PGA or PGA *slope is strongly increased by the “outlier” with a 2015 increase of 2500. I think readers may want to know 1) where is this grid point (Show it in Fig 7 for ex) 2) if the authors could comment in one sentence about the 2015 landsliding in this grid point of why the ET was so much larger than elsewhere… (like is it a glacial valley ? A canyon ? Specific lithology ? Peculiar landslides ?)
I am not asking the authors for a new paragraph, but a sign in Fig 7 and a sentence of caveat about the influence of this single grid point which may be special for X, Y reason.

L448 : Optimistic. I strongly suggest instead: “and that it could be caused by a combination of the earthquake and rainfall, or be an artifact arising from the imperfect rainfall data and normalization (cf Fig 3a and 3b).”

This is important given that the 1989 perturbation is not very large and does not even exist in the normalization based Persiann CDR.

L509: “did not compound”

L521: I guess it should be 5-30yr to be consistent.

Reviewer Comments and Responses

Below are our responses to the final few reviewer comments. The line numbers in our responses correspond to the tracked changes manuscript. Note, the other changes in the track-changes manuscript are minor phrasing edits made to keep the word count < 5,000.

L219 : "removed" feels awkward to me in this sentenec though I see what you mean.
What about : "how many standard deviations separate a given observation from the mean of the population (containing this observation) / (it is drawn of)."

Response: changed as suggested, lines 201 – 202.

L250: I think there is no need to try harder in the context of this paper, but in case the authors plan future work on this topics, I do not think that looking at successive daily anomalies above a threshold (derived from daily anomalies) is a statistically correct way. What I was suggesting is to compute anomalies at different scale, and it would give a different results. Because if you have 3 successive daily anomalies of 8-12-9 you cannot know what anomaly it would represent in triad Z score : because the mean and standard deviation for triplets of days would be different. And because the probability of successive, localized intense rainfall events is low, the final results could change quite a bit. Feel free to contact me if you want to discuss and work along these lines in the future.

Response: No change required in the manuscript.

L306 : "Does not consider anomalous rainfall over shorter or longer scales"
and L308: " across multiple days of few hours using rainfall products with higher temporal resolution "
I think this helps to stay general as clearly some landslide clusters could be triggered by <24h intense events which would be distorted (tune down most likely) in daily records.

Response: Changed both lines as suggested, lines 282 – 286.

Figure 6: the hashed lines seems wrongly labelled ? Isn't it rather the extent of extreme rainfall of 2002 ? given 2002 mass wasting is already in pink

Response: The reviewer is correct, and this has now been changed on the figure.

Figure 8, S5,S6,S9 : In all figures with PGA / ET could the authors specify in label or caption the units (g for PGA, but what for ET for example ? Is it a surface ? A volume ?

Response: added units as requested (g for PGA, and g.m^3 for PGA*ET).

L427-432 : Related to that it is quite clear that the difference in R2 between PGA*ET vs PGA or PGA *slope is strongly increased by the "outlier" with a 2015 increase of 2500. I think readers may want to know 1) where is this grid point (Show it in Fig 7 for ex) 2) if the authors could comment in one sentence about the 2015 landsliding in this grid point of why the ET was so much larger than elsewhere… (like is it a glacial valley ? A canyon ? Specific lithology ? Peculiar landslides ?)
I am not asking the authors for a new paragraph, but a sign in Fig 7 and a sentence of caveat about the influence of this single grid point which may be special for X, Y reason.

Response: We have added a couple of sentences caveating the influence of this outlier point (grid cell 76), and outlining that this grid cell had the highest PGA-excess topography values as this was one of the few cells of its latitude (where this region typically has the highest average excess topographers) to overlap with the greatest Gorkha earthquake PGAs. And that this cell also had the highest 2015 mass-wasting increase, as it experienced 3.6x106 m3 of landsliding (including one landslide > 220,000 m2), whereas in the preceding 25 years this cell only experienced > 1.0x106 m3 of landsliding in three other years (lines 377 – 384). We also show the location of this grid cell in Figure 7 as suggested.

L448 : Optimistic. I strongly suggest instead: "and that it could be caused by a combination of the earthquake and rainfall, or be an artifact arising from the imperfect rainfall data and normalization (cf Fig

3a and 3b).”
This is important given that the 1989 perturbation is not very large and does not even exist in the normalization based Persiann CDR.

Response: Phrasing changed as suggested – lines 398 – 400.

L509: “did not compound”

Response: Corrected, line 448.

L521: I guess it should be 5-30yr to be consistent.

Response: Changed, line 457.
